# Educational Expansions and Fertility: Evidence from Norwegian College Reforms

**DOI:** 10.1007/s10680-025-09737-7

**Published:** 2025-06-02

**Authors:** Adrian Farner Rogne, Agnes Fauske, Rannveig Kaldager Hart

**Affiliations:** 1 Institute for Social Research, Oslo, Norway; 2 Department of Sociology and Human Geography, University of Oslo, Oslo, Norway; 3 Institute for Church, Religion, and Worldview Research, Oslo, Norway; 4 Institute of Health and Society, University of Oslo, Oslo, Norway; 5 Centre for Fertility and Health, Norwegian Institute of Public Health, Oslo, Norway

**Keywords:** Fertility, Family formation, College reform, Norway, Difference-in-differences

## Abstract

Previous research has pointed to educational expansions and increased educational attainment among women as an important factor contributing to reductions in fertility levels and changes in family formation patterns in the second half of the twentieth century. We investigate one potential mechanism that may have contributed to these developments—the establishment of local colleges providing access to higher education for broader segments of the population. We study the establishment of regional colleges in Norway 1973–83. Taking advantage of population-wide administrative register data, historical data sources and recent developments in difference-in-differences methodology, we employ an event study design to assess the impact of local colleges on cohort fertility and age-specific fertility outcomes, as well as age at marriage and regional mobility as potential mechanisms. Our findings suggest that access to local colleges did not impact fertility, family formation or mobility to any substantial degree.

**Supplementary Information:**

The online version contains supplementary material available at 10.1007/s10680-025-09737-7.

## Introduction

Over the last half of the twentieth century, educational expansions transformed Western societies. The same period also saw substantive reductions in total fertility rates (TFRs) in many such national contexts (OECD, 2023b). This study investigates whether structural changes in the local availability of higher educational institutions in this period were causally linked to local reductions in fertility and postponement of childbearing in Norway.

Higher education is linked to delayed childbearing, largely due to very low fertility rates among women enrolled in education (Lappegård & Rønsen, 2005; Ní Bhrolcháin & Beaujouan, 2012). Evidence on the relationship between educational attainment and completed family size is more mixed, and the relationship arguably depends on institutional context (Blossfeld & Kiernan, 2019; Oppenheimer, 1997; Rendall et al., 2010). Women who pursue higher education may have different preferences and values from the outset, so that fertility differences emerge due to selection effects (Kreyenfeld, 2002). Thus, a number of studies have used educational reforms as natural experiments to identify the causal effects of education on fertility (Cygan-Rehm & Maeder, 2013; Fort et al., 2016; Kountouris, 2020) Such identification hinges on the assumption that reforms affect fertility through changes in women’s educational attainment only. Bharati et al. (2023) challenge this assumption, showing that an educational reform in Taiwan also impacted the fertility of women whose educational outcomes were not influenced by the reform.

As emphasized by Bharati et al. (2023), local educational expansions have the potential to change the demography and labour market structures of regions in several ways, of which many have the potential to impact fertility—beyond any effects running through educational attainment. Most importantly, local college expansions may selectively impact decisions of internal migration, allowing regions to retain, in particular, young adults with higher educational aspirations. Young adults induced to stay will be exposed to a different partner market than if they had moved, with potential implications for their own prospects of finding a partner and subsequently having children. Similarly, an influx of college students from other regions may affect the partner market also for those whose educational decisions are unaffected; the new regional inhabitants may provide potential partners for some, and competitors in the partner market for others. If preferences for educational field are gender segregated, specialized regional colleges may lead to imbalances in the local partner market, making it more difficult to find a mate for those with a preference for the under-represented sex (Eckhard & Stauder, 2018). In short, local college expansions may affect fertility through demographic processes related to migration and partnership formation.

Our paper tangents one of the key debates in the fertility literature by studying the causal link between local educational supply and childbearing. We study the impact of Norwegian educational expansion reforms on fertility by exploiting geographic and temporal variation in the supply of higher education at the regional level, generated by a set of major educational reforms. Focusing on the opening of local colleges^1^ in the period 1973–1983, we employ a difference-in-differences/event study design that allows us to assess if cohorts exposed to the opening of local colleges experienced changes in fertility and family formation patterns. Our comparison group is men and women of the same age, residing in regions where no educational institutions were opened, or in regions where a college opened later. Specifically, we assess the impact of the opening of local educational institutions on the number of children born before age 40, and on the probability of having at least one child, and the probability of being married, as measured every fifth year from ages 20–40. Additionally, we assess whether local colleges may have affected individuals’ propensity to remain resident in their region.

Rogne et al. (2024) found that the establishment and institutional upgrading of such institutions had no impact on the educational attainment of males and at most had a modest impact on the educational attainment of females. In a similar analysis, Knutsen, Modalsli, and Rønning (2022) found some evidence that the field of education was impacted, but also no impact on educational attainment. Data on student admission indicate that local students were heavily overrepresented among those enrolled at local colleges (Brattsvåg and Carlsson, 1981). Taken together, this suggests that local colleges affected where, but not whether, young adults got a higher education. Thus, any reform effects on fertility must be mediated by other demographic or value changes, induced by the educational expansion.

Our study makes two main contributions. First, we use detailed data and state-of-the-art methods to assess how local educational expansions influence multiple dimensions of demographic processes: fertility, marriage rates, internal migration and population size. Second, we make a methodological contribution to a small but growing literature using local reforms as an instrumental variable (IV) for (local) educational attainment. Educational attainment is not impacted in our sample, allowing us to make an indirect test of the crucial assumption of no indirect effects in IV estimation.

## The Norwegian Context

Higher education was, and is still today, free in Norway. As early as in 1947, the Norwegian State Educational Loan Fund (‘Lånekassen’) was established, supplying stipends and universal student loans with low interest rates to cover living expenses while studying. This was established as a means to make higher education available to all, independent on their social origins (Lånekassen.no, 2023). The second half of the twentieth century was marked by substantial increases in educational attainment—especially among women (Birkelund & Petersen, 2016).

During the period 1973–1983, when the colleges we study were opened, the total fertility rate (TFR) dropped from 2.23 to 1.66. Since then, the TFR has fluctuated below 2, before the fertility fall following 2010 (Statistics Norway, 2023; World Bank, 2023). Several other relevant changes happened in the same period. For instance, although effective contraceptives (contraceptive pills and copper IUDs) were introduced in the late 1960s, and thus available in 1973, their uptake gradually increased throughout the 1970s (Nesheim, 2017, 2023; Walløe & Bauck, 1978). However, since effective birth control was made available to all before our study period starts, there is little reason to believe that this would confound our results.

During the 1970s and 1980s, the support for students and working parents with young children was scarce (Ellingsæter & Leira, 2006), thus more comparable to other welfare regime contexts of today (e.g. the Anglo-Saxon context characterized by a low level of support to families (Bergsvik et al., 2021). Until 1977, only mothers were eligible for 12 weeks of paid maternal leave. In 1977, the leave was extended by 6 weeks (Lillebø et al., 2024). Mothers and fathers were given equal rights to take up leave, but mothers continued to use most of the leave. The 1977 extension of the leave did not impact completed fertility and is thus unlikely to influence our results (Carneiro et al., 2015). No further extensions of the leave were made during the period in which the local colleges in this study were established (1973–1983) (Ellingsæter & Leira, 2006).

Childcare was acknowledged as a public responsibility in 1975 when the Act on Day Care Institutions was enacted. Since then, there has been a steady increase in the availability of childcare services, but the public day care supply was still scarce at the end of our reform period (Ellingsæter & Leira, 2006).

## Theoretical Framework and Empirical Studies 

### The Effects of Educational Enrolment and Attainment on Fertility

While the reforms we study did not impact educational attainment, our study is situated in a literature of higher education expansions that are expected to impact fertility mainly through this precise channel. Thus, a brief description of the theoretical foundation for this expectation is worthwhile. The general theoretical expectation is that higher education increases earnings potential, which means that more earnings are lost when parents (initially mothers) take time off work to care for young children (Becker, 1960, 1991). When institutional support to parents reduces this opportunity cost, a positive relationship between women’s education/earnings potential and fertility may emerge (Doepke et al., 2023).

In the cohorts we study, the relationship between education and fertility is negative for women (Jalovaara et al., 2019; Kravdal & Rindfuss, 2008). However, individuals who pursue higher education may have different childbearing preferences from the outset. To understand the causal relationship between education and fertility, reform studies that use arguably quasi-random or exogenous variation in educational attainment are required. Monstad et al., (2008) investigated the effect of an educational reform in Norway that increased the number of years of compulsory schooling from seven to nine years that was implemented over a 12-year period from 1960 to 1972, exploiting variation in the timing of the reform across municipalities. They showed that the reform led to the postponement of childbearing but had no effect on completed fertility. Research on similar compulsory schooling expansions has showed mixed results, indicating that contexts matter. Evidence from Germany and England has suggested negative effects of compulsory schooling reforms on completed fertility, which the authors attributed to the particularly high costs of childrearing in Germany and inflexible labour market systems (Cygan-Rehm & Maeder, 2013; Fort et al., 2016). In other European contexts, no evidence of effects on completed fertility was found (Fort et al., 2016; Kan & Lee, 2018). Koebe and Marcus (2022) show that a more compressed education leads to earlier births among young adults on an academic track. However, unlike the reform in our study, which mainly affected where it was possible to attain higher education, these studies investigated reforms that affected only the length of education.

Few studies have investigated the consequences of reforms in higher education. Greek reforms in 2000 expanded higher educational opportunities by (1) increases in supply through the creation of many new higher education institutions across the country and increasing the number of places in already existing programmes and (2) changes in the university entry system which allowed access to students with lower grades. These reforms reduced the probability of giving birth before the age of 30 by 20 percentage points for those women who attained higher education due to the reform. The decrease in fertility seemed to be mediated through improved career opportunities and increased opportunity costs (Kountouris, 2020). A small number of studies have investigated the impact of secondary and higher education on fertility outcomes and show a causal effect of education on fertility timing among women (Grönqvist & Hall, 2013; Humlum et al., 2017; James & Vujić, 2019). The overall impression from these studies is that educational reforms impact timing, but rarely completed family size. However, these reforms involved changes in the supply of higher education through changes in college admission requirements, number of students enrolled and the transformation of polytechnic institutions to universities, plus changes in the high school exam system, curricula and prolongment of vocational programmes. An exception to this pattern is Virtainen et al. (2024), who utilize discontinuities in admission into secondary and tertiary education in Finland. They find that women with higher education are more likely to find a partner and have children, while there is no effect for men.

Previous studies of these reforms in the Norwegian context have also found them to not impact the social origins gradient in recruitment to higher education (Rogne & Frisli, 2024) but have found them to impact skilled wages (Carneiro et al., 2023).

### Educational Expansions and the Local Partner Market

A local educational expansion can impact the composition of the local partner market, by attracting young people from other regions, and retaining young adults who would otherwise have moved out (Fletcher & Noghanibehambari, 2021). These changes could impact the partner market for local residents, albeit in different ways depending on their sex and socioeconomic status (Eckhard & Stauder, 2018).

More than 90% of Norwegian children are born to a mother in a coresident relationship (NIPH, 2023). In Norway as in other contexts, there is clear evidence of assortative mating—parental couples are similar in terms of their education and income decile (Bratsberg et al., 2023). Earnings hypergamy, where women match with a partner with higher earnings, remains common also in Norway (Kotsadam & Moen, 2023). This suggests a pattern where men with lower earnings potential may face more difficulty in finding a partner and thus remain childless when female educational attainment increases (Bratsberg et al., 2021). When a local college is established, an influx of men with higher earnings potential could crowd out men with lower earnings potential in the local partner market. Thus, the establishment of local colleges could increase childlessness among local men with lower earnings potential (Lappegård et al., 2011).

### Contextual Effects

Theories of value-based change (e.g. Lesthaeghe, 2010, 2014) suggest that pursuing higher education changes values, leading individuals to emphasize freedom and independence over family formation and commitment. The establishment of a college could affect the value placed on education and work careers among women, fostering more independence and a stronger commitment to life goals outside the family sphere, also among women who were not moved to complete higher education. This could in turn affect their fertility plans and family formation patterns. To the extent that improved local educational opportunities lead to such value changes, they can spread throughout local communities through ‘social contagion’ (see, for example, Zaidi & Morgan, 2017; Balbo & Mills, 2011). Through value contagion, the establishment of a local college could impact marriage and fertility rates also among those whose educational outcomes are unmoved.

The establishment of local colleges may also affect fertility and family formation patterns through other contextual channels, such as by altering the local labour market and causing shifts in the availability of skill-intensive jobs and the demand for educated workers (Carneiro et al., 2023). Such effects may be very different for those who respond to college openings by attaining higher education and those who do not, but our study design does not allow us to separate these mechanisms empirically.

### Regional Retention

While we maintain a focus on fertility and partnering, we also assess whether local colleges retained individuals in their respective regions. Access to education locally may induce individuals to remain resident in their local region, rather than moving to study elsewhere, which may impact both partnership formation, childbearing and population development.

### Expectations

We assess whether local colleges affected fertility patterns and suggest three main mechanisms through which such effects could run, based on the theories and empirical studies outlined above.

1. *Changes in the local partner market:* Local educational expansions may affect some young adults to stay in their region of origin and encourage others to move in. Depending on the nature of the shifts, this could make it easier or more difficult for local young adults to find a partner.
2. *Value changes:* When a local college is established, values regarding women’s role in society and preferences regarding family, education and work careers may change locally, also for women and men who do not attend college. Value-based theories of demographic changes suggest that this could lead to a fertility decline.
3. Local colleges may also affect fertility patterns locally though other, complex and heterogeneous pathways, such as by impacting *local labour markets*.

## Reform Details

Until the late 1960s, higher educational institutions in Norway—universities and major colleges—were almost exclusively located in a handful of major cities. These offered educations in a limited range of subjects, and urban males from upper-class backgrounds were overrepresented among the students (Helland & Wiborg, 2014; Kyvik, 2014). The expansions of the higher educational system were motivated by several factors. Expansions of the high school system meant that the number of graduates eligible for higher education, especially women, grew rapidly in the 1950s and 1960s. This resulted in a growing demand for higher education that put pressure on the student capacity at existing institutions. Alongside this was an increasing demand for skilled workers in emerging industries and the expanding public welfare sector. These developments coincided with strong political currents that favoured making higher education more accessible to all, and a wish among policymakers to diversify the educational system in terms of fields of study and the length of educations. The notion that there was an untapped ‘talent reserve’ in rural areas, combined with a wish to promote local economic, cultural and social development were therefore also central to the motivation for a set of sweeping reforms of the higher educational system. The reform period started with the establishment of the first ‘district colleges’ in 1969 and lasted until a major reform resulted in the merger of a large number of institutions in 1994 (Kirke- og undervisningsdepartementet, 1969b; Kyvik, 2014).

Our data cover universities, colleges and post-secondary, non-tertiary institutions that at some point in time became recognized as tertiary educational institutions in Norway between 1965 and 1993.^2^ The total number of such institutions grew from 109 to 202 between 1965 and 1992, while the number of universities and colleges grew from 17 to 201.^3^ In other words, around half of all new universities and colleges were upgraded from previously existing institutions. These reforms entailed a massive decentralization of the higher educational system. These developments were the result of several processes. One was the opening of new institutions, and in particular 15 ‘district colleges’ (Kirke- og undervisningsdepartementet, 1966, 1967, 1968, 1969a, 1969b, 1972, 1973). Each of these provided education in specific vocally oriented fields (IT, business management, media and communication, etc.), but they additionally offered educations in other fields. Students from the local region were heavily overrepresented at such institutions (Brattsvåg, 1975a, 1975b, 1976, 1977, 1978; Brattsvåg and Carlsson, 1981). In addition to these 15 major colleges, several other new college-level institutions were also opened in this period (Frisli & Rogne, 2023a, 2023b).

Another important process was the institutional upgrading of many existing vocational post-secondary, non-tertiary institutions. This occurred through processes involving a standardization of curriculums and lengths of educations, the introduction of high school graduation as a standard admission requirement,^4^ and institutional mergers, and resulted in many such institutions becoming part of the higher educational system in Norway (Frisli & Rogne, 2023a, 2023b; Smeby & Terum, 2023; Terum & Smeby, 2014). While such institutional upgrading implied important changes to the institutions, we do not have strong reasons to expect that the reclassification changed the effects of educations taken at such colleges on fertility, except, perhaps a short postponement effect due to the lengthening of educations and thus enrolment time. As they generally did not lead to sharp increases in student capacity, they likely did not entail sharp changes in the local partner market. We also do not have strong reasons to expect that the labour market prospects of the graduates changed substantially after institutional upgrading. Most importantly, the impacts of institutional upgrading would likely have been smaller than the impacts of establishing new institutions. This is because such educations provided qualifications for the same jobs as previously and were also ‘reclassified’ as higher education administratively when the institutions were reclassified.^5^ Thus, we focus on the establishment of new post-secondary, non-tertiary or college institutions in regions that previously had none, as these entail a substantive change in the local educational opportunities and potentially also in the local partner market. In our design, we compare cohorts in the 7 regions that were treated by the establishment of one or more institutions at some point in the period 1973–1983, to the 49 regions that consistently did not have an institution, and thus remained in the control group (see definition of regions below), and to untreated cohorts in the treated regions.

## Empirical Strategy

### Identification Strategy

**Fig. 1:**
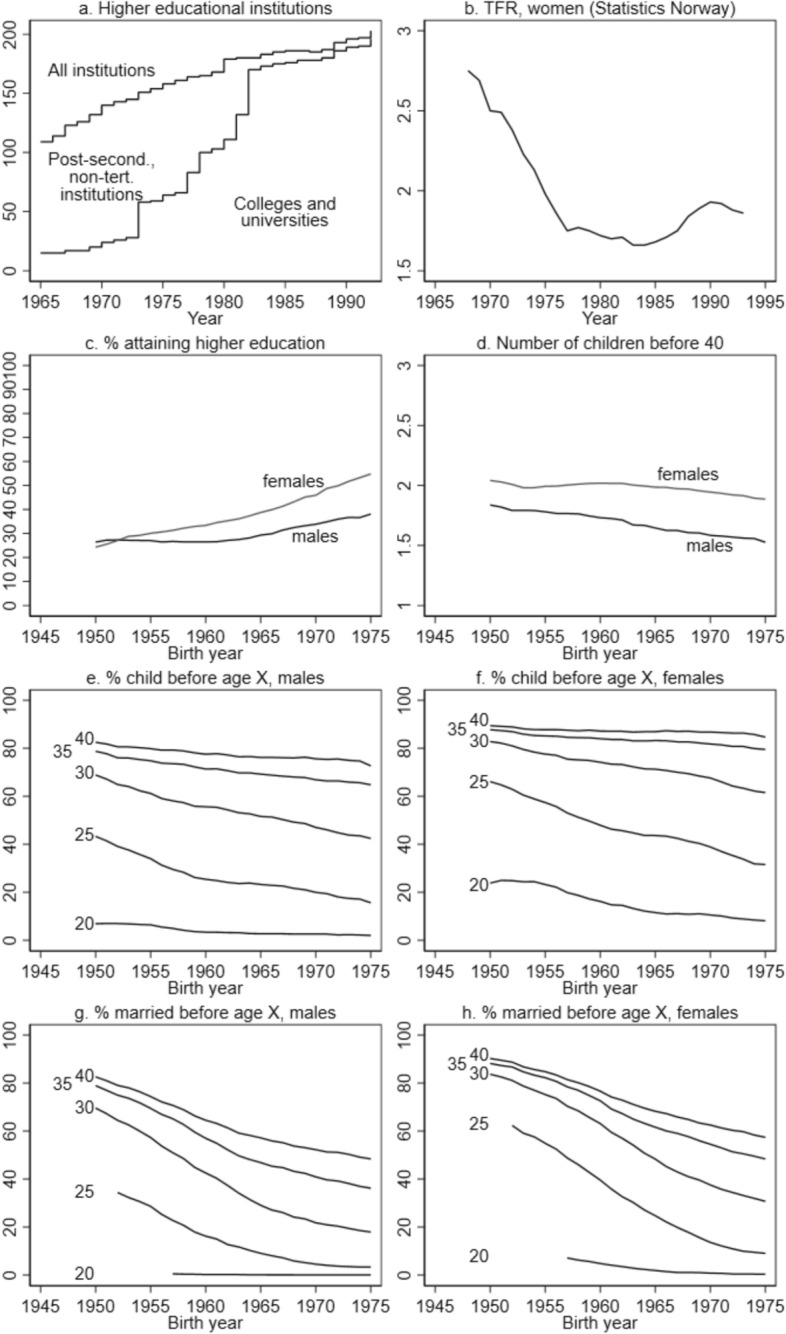
Trends. *Note*: Panel a. is based on Frisli and Rogne (2023a, 2023b). Panel b. is based on Statistics Norway’s calculations. Panels c.-h. are based on administrative registers, conditioned on residency at age 17

Figure 1 provides a description of the overall trends under study, based on a total population sample covering the entire reform period. Panel a. shows the number of educational institutions included in our data, where we separate between those classified as universities or colleges, and those classified as post-secondary, non-tertiary institutions, illustrating the expansion of higher education and the large-scale institutional upgrading in this period. Panel b. illustrates the decline in period fertility (TFR), using Statistics Norway’s calculations (Statistics Norway, 2023). Panel c. shows the proportion attaining a higher education in the cohorts 1950–1974, separately by sex, illustrating the strong growth in female higher educational attainment and the reversal of the gender gap. Panel d. illustrates the gradual decline in total fertility by age 40 among both males and females over the cohorts under study. Panels e. and f. show the proportion having one or more children before different ages among males and females, respectively, visualizing the trends towards postponement of childbearing over the period. Panels g. and h. show trends in the direction of both postponement of marriage, and an increase in the share not married by age 40, among both males and females. In total, this figure illustrates trends in educational expansions, educational attainment, fertility and family formation patterns. While we note that period fertility declined most in the early period, cohort fertility was most reduced for later cohorts, while all cohorts were part of the postponement of childbearing and marriage.

Our aim is to test whether the trends observed in Fig. 1 are causally related at the local level. To this end, we analyse the effect of the establishment of eight local educational institutions in seven regions on fertility and family formation in a difference-in-differences/event study design. The basic idea of this design is best illustrated by the special case of comparing one region where a regional college is established (‘treated’) to another region where no college was established (‘control’). In the treated region, we would observe the fertility outcomes in one cohort unaffected by college establishment because they were past the common college enrolment age, and one that was affected, because they reached college enrolment age after the college opened. In the control region, no cohorts would be treated. One can calculate a simple difference-in-differences (DiD) estimate as the difference between the change in the treated region and the change in the comparison region (Angrist & Pischke, 2009):

$$\text{DiD}=\left({\text{FERT}}_{\text{treat},\text{post}}-{\text{FERT}}_{\text{treat},\text{pre}}\right)-\left({\text{FERT}}_{\text{contr},\text{post}}-{\text{FERT}}_{\text{contr},\text{pre}}\right)$$

As regional colleges were established at different time points in different regions, our reform does not fit with this basic approach. A common extension of the DiD model for such cases has been the two-way fixed effects (TWFE) design (Angrist & Piscke, 2009), where a dummy variable for being treated (in our case, having access to a local college) is combined with time and region fixed effects. Recent methodological advances have shown that such models yield biased results if treatment effects are heterogeneous and/or dynamic, meaning that effects vary over time and across treatment groups (Goodman-Bacon, 2021). This is likely in our case: institutions differed by size, type and field, and the student capacity at local colleges changed considerably over time (Frisli & Rogne, 2023a, 2023b). To avoid such biases, we follow the literature in estimating event study difference-in-differences models (Roth et al., 2023) using a model specification that can handle heterogeneous and dynamic treatment effects. These models allow us to construct a comparison group of never-treated and not-yet treated regions and allow effects to vary over treated cohorts.

The identifying assumption in our design, as in a standard difference-in-differences design, is that the trends in the outcome would be parallel in the treatment and control group, and independent of treatment timing, absent the reform. This assumption cannot be tested directly, as we cannot observe the outcomes in the treatment group in the absence of treatment. We perform two indirect tests of this identifying assumption. First, we assess whether the development in the treatment and comparison group is comparable prior to treatment. Note that in our application, cohorts included in the pre-period can also be defined as partially treated, so that some ‘anticipatory effects’ (or in our case, effects of partial treatment) could be expected. A second indirect test of the validity of our design is whether potential confounders are conditionally independent of the establishment of local colleges. To test this, we estimate two-way fixed effects models with the establishment of a local college as the outcome, and region and time fixed effects as well as our control variables as predictors. If coefficients for the control variables are statistically insignificant, it supports the assumption that college establishment is conditionally random in our design.

### Data and Sample

We base our analyses on individual-level data and use geographical regions as our treatment groups. These regions are based on the 2002 standard for economic regions developed by Statistics Norway to correspond to the EU Nomenclature of territorial units for statistics (NUTS-4) standard, meant to capture economic regions with commuting distance to the regional centre and integrated labour markets. We have made some minor adjustments to the standard to allow for changes in the official municipality structure in the years between 1968 and 2002. This produces a total of 89 regions.

Our sample consists of individuals who are Norwegian residents at age 17. Individuals are linked to the region they resided in on January 1 when they were 17 years old (or they year they would turn 18), avoiding issues related to endogenous moves to regions with colleges following high school completion.^6^ The data set consists of one record per individual, so that when fixed effects for cohorts are included, there is no variation by calendar year.

We restrict our sample to individuals born in the years 1950–1974. Our focus is on a subset of regions where institutions were established in the years 1973–1983. In our main specifications, we assess the impact of college access that individuals would face at age 20. We follow cohorts in the treated regions from 5 years before treatment to 10 years after.

We run our models on the entire sample, as the estimation command handles comparisons with always-treated and already-treated units. For models where the outcome is the total number of children born by age 40, and the treatment is the presence of educational institutions, measured at age 20, this sample consists of 169,751 females and 180,738 males in never-treated regions, 17,604 females and 18,624 males in pre-treatment cohorts in treated regions and 32,637 females and 34,506 males in post-treatment cohorts in treated regions. In alternative model specifications with varying treatment ages, which cohorts are considered treated varies accordingly.

Although the reforms primarily targeted high school graduates, we have opted not to restrict the sample to high school graduates for two reasons. First, high school graduation may be endogenous to the local availability of colleges. The establishment of a local college requiring high school completion may have prompted some individuals to attend or complete high school. Second, while most colleges required high school graduation for admission, many vocational post-secondary, non-tertiary schools and some colleges did not, or had a lenient practice for granting admission to students who did not meet this requirement (See for instance Brattsvåg & Carlsson, 1981: 3).

### Definition of Treatment

In our design, individuals are defined as treated if there is a college in their region when they are ‘of college enrolment age’. We code the treatment variable as 1 for individuals who at age 17 live in a region that has a college when they are aged 20, and 0 otherwise. This coding is chosen based on information on the modal ages for college entry. In the Norwegian system, the standard is that individuals graduate from high school the year they turn 19. Modal age of college entry in this period was somewhat higher, at 20–21. It was on average higher for males due to mandatory military or civil service (Nåvik, 1996). However, individuals can (and often do) wait up to several years after high school graduation before entering college (Raabe, 2002).^7^ We test whether our results are sensitive to using the availability of regional colleges when individuals were 18, 22 and 24 years old as the treatment.

In regions where no colleges were opened, all birth cohorts will have zero on the treatment variable. In cohorts where a college was established in the observation period, individuals who were above age 20 when the college was established are defined as untreated, while individuals who were 20 years or younger when the college was established are defined as treated. The central comparison in our models is between 49 regions that remain untreated (with no college) throughout the period, and seven treated regions that received their first institution in the observation period (1973 (2 regions), 1979, 1980 (2 regions) and 1983 (2 regions)). Details on these regions are provided in Table 1. Regions that had a college or post-secondary, non-tertiary institution throughout the period, including regions that upgraded previously existing institutions or established additional institutions, do not contribute to estimating our parameters of interest.

**Table 1:** Treated regions

Region number	Region name	First PSNTI/college established	First institution name	First institution field
1493	Sogndal	1973	Sogndal Lærerskole	Teacher education/theology
2093	Alta	1973	Alta Lærerhøgskole	Teacher education/theology
1494	Førde	1979	Sogn og Fjordane Sjukepleiarhøgskole, Førde	Healthcare/miscellaneous
1193	Haugesund	1980	Haugesund Maritime Høgskole Haugesund Sjukepleiarhøgskole	Engineering/maritime/ tech/STEM Healthcare
1791	Steinkjer	1980	Nord-Trøndelag distriktshøgskole	Sports/military/miscellaneous
192	Moss	1983	Eurytmiskolen i Moss	Art/dance/music/theatre
1991	Harstad	1983	Høgskolen i Harstad	Healthcare

### Outcome Variables

To assess impacts on total fertility, we use a variable measuring the total number of children each individual had before age 40 (TNC40). Age 40 is used because we only have data on childbearing until 2014, meaning that we can follow the latest cohorts affected by the reform period to about this age. While births above age 40 are not captured in our measure, it remains a strong proxy for completed fertility. To assess effects on postponement of childbearing, or age at first birth, we use dummies indicating whether the individual had a child before age 20, 25, 30, 35 and 40 (CB20-CB40). First births after age 40 are relatively rare, so being childless at this age is a strong proxy for permanent childlessness. If the reform effects pertain to delayed births, we will see negative effects at younger ages, followed by no effects at our proxy for completed fertility (age 40). We do not use age at first birth as an outcome, as it is missing for those who remain childless, and thus defined only in an endogenously selected sample.

We also include an indicator of being married as a proxy for the aspects of family formation not captured by childbearing. In the cohorts we study, births to cohabiting couples became increasingly common. While this makes marriage an imperfect proxy of family formation, it remains the best proxy available in administrative registers for the period we study. However, we note that the results from this analysis should be interpreted with some caution, as they capture only part of the family formation process, and because the relationship between marriage and childbearing changes over time as cohabitation becomes more common. Among women born in the 1950s, the oldest in our sample, 51% had cohabitation as their first union, and this had increased to 80% among women born in the 1960s (Noack, 2010:116). Over these cohorts, a declining share of first cohabitations ended in marriage (64 vs. 42 per cent), and an increasing share was dissolved (27 vs. 37 per cent) (ibid). In this sense, marriage becomes a weaker proxy for family formation over the cohorts we study. Nevertheless, these analyses are informative about the impact of local colleges on the postponement and decline in marriage. We construct a set of dummy variables indicating whether the individual was ever married before age 20, 25, 30, 35 and 40 (MB20-MB40). Marriage records are only available from 1975 onwards, meaning that we have to omit some early cohorts when studying marriage before ages 20 (1950–1956) and 25 (1950 and 1951).

Finally, we also assess whether individuals at age 25, 30, 35 and 40 reside in the same region as they did at age 17 (SR25-SR40). For this, we use a dichotomous outcome variable taking the value 1 if they reside in the same region, and 0 otherwise.^8^

### Statistical Method

We estimate event study models, taking the following basic form:

$${y}_{i}=\sum_{k=-4}^{10}{\beta }_{k}{D}_{i}^{k}+{\theta }_{c}+{\theta }_{r}+{\varepsilon }_{i}$$

Here, ${y}_{i}$ is the outcome of interest (e.g. number of children at age 40). Cohort fixed effects ${\theta }_{c}$ net out changes over time that are common across regions, and region fixed effects ${\theta }_{r}$ net out time constant differences between regions.^9^

Our parameters of interest are the coefficients for the dummy variables indicating the duration in years (*k*) since college establishment. For example, *D*^0^ = 1 for individuals in the first treated cohort in a region (aged 20 when the regional college opened), and zero for all other groups. *D*^1^ = 1 for individuals in the second treated cohort (aged 19 when the regional college opened), zero for all other groups. *D*^−1^ = 1 for the last untreated cohort (aged 21 when a local regional college opened), and zero for all other groups. Note that this group can also be considered partially treated, as the college expansion opened educational opportunities for them, too, though college enrolment was less common at older ages. By convention, *D*^−1^ is the omitted reference category, in which individuals who resided in regions where no college was established are also included. Statistically significant coefficients for dummies for the pre-reform period (negative values of *k*) would indicate that the identifying assumption of parallel trends absent college establishment does not hold. The minimum values shown of the duration variable are − 5, and the maximum value is 10.

We estimate our models on individual-level data aggregated to region-by-year cells using the csdid command in Stata (Callaway & Sant’Anna, 2021). We specify the long2 option for a conventional interpretation of the pre-trends. This estimator handles issues related to heterogeneous effects in the case of staggered treatment and identification of long term effects that may bias standard event study models (Borusyak et al., 2022; De Chaisemartin & d’Haultfoeuille, 2022; Roth et al., 2023). We report the average of the pre- and post-treatment estimates, with confidence intervals. We also tested the null hypothesis that all pre-trend estimates are equal to zero using the *estat pretrend* command. As described above, the nature of our design leads us to expect some anticipatory effects: The oldest cohorts in the pre-period in the treated regions are also subject to improved local educational opportunities, albeit at a higher age than the average age of enrolment. The direction of any anticipatory effects is expected to follow the direction of effects, which again may vary between outcomes. In light of this nuance, the test of the pre-period average is chosen as our main guide for interpreting the results.

We include several control variables aggregated to region-by-year cells: the educational level of each of the individual’s parents, the age of the parents, the number of maternal siblings and the total population of the region. Each parent’s education is coded in ten categories—nine categories representing educational levels, and one indicating missing information. These variables are used in balance test to assess whether the timing of college establishments was systematically related to characteristics of potential students in the region. They are also included in event study models in the supplementary material, to net out potential imbalances on observable characteristics, and potentially also improve precision of the estimates.

## Results

### Descriptive Statistics and Balance Tests

Descriptive statistics for never-treated regions and treated regions 5 years prior to treatment and 10 years post-treatment are provided in Table 2. These statistics are calculated at the region-level weighted by the number of observations in each year-by-sex-by-region cell. The overall trends shown previously are also clearly visible in this table, illustrated by the increased proportion attaining higher education and the reduction in fertility and marriage in the post-treatment period. A corresponding table of descriptive statistics for our control variables is provided in the supplementary information (S1). Note that only the treated regions have a pre- and post-treatment period. The never-treated regions are considered untreated throughout the period. Graphical descriptions of the trends in outcome variables and control variables by region are provided in the supplementary information (S2).

**Table 2:** Descriptive statistics

	Males				Females			
	Never	Pre	Post	Diff	Never	Pre	Post	Diff
Attained higher education								
Mean	0.26	0.27	0.30	0.03	0.35	0.30	0.40	0.10
SD	0.06	0.04	0.06		0.10	0.05	0.07	
CB20								
Mean	0.04	0.05	0.03	− 0.02	0.17	0.22	0.16	− 0.06
SD	0.02	0.02	0.01		0.08	0.06	0.07	
CB25								
Mean	0.28	0.32	0.26	− 0.06	0.50	0.58	0.49	− 0.09
SD	0.09	0.07	0.05		0.11	0.08	0.08	
CB30								
Mean	0.55	0.60	0.55	− 0.05	0.75	0.79	0.75	− 0.05
SD	0.08	0.05	0.05		0.07	0.04	0.05	
CB35								
Mean	0.71	0.74	0.71	− 0.03	0.85	0.87	0.85	− 0.02
SD	0.05	0.04	0.04		0.04	0.03	0.03	
CB40								
Mean	0.78	0.80	0.78	− 0.02	0.88	0.90	0.88	− 0.02
SD	0.04	0.03	0.03		0.03	0.02	0.02	
TNC40								
Mean	1.71	1.84	1.76	− 0.08	2.03	2.14	2.11	− 0.03
SD	0.16	0.18	0.16		0.17	0.17	0.18	
MB20								
Mean	0.00	0.00	0.00	0.00	0.02	0.07	0.03	− 0.04
SD	0.00	0.00	0.00		0.03	0.03	0.03	
MB25								
Mean	0.13	0.24	0.13	− 0.12	0.31	0.52	0.30	− 0.22
SD	0.10	0.10	0.08		0.19	0.12	0.14	
MB30								
Mean	0.37	0.52	0.35	− 0.18	0.55	0.73	0.53	− 0.20
SD	0.18	0.11	0.12		0.19	0.09	0.14	
MB35								
Mean	0.53	0.65	0.51	− 0.14	0.67	0.80	0.66	− 0.14
SD	0.16	0.10	0.11		0.15	0.08	0.11	
MB40								
Mean	0.61	0.71	0.60	− 0.11	0.72	0.83	0.72	− 0.12
SD	0.13	0.09	0.09		0.12	0.07	0.09	
SR20								
Mean	0.86	0.93	0.93	0.01	0.75	0.80	0.84	0.04
SD	0.18	0.03	0.02		0.18	0.07	0.06	
SR25								
Mean	0.69	0.74	0.77	0.02	0.54	0.63	0.65	0.02
SD	0.16	0.06	0.05		0.14	0.07	0.07	
SR30								
Mean	0.59	0.67	0.66	− 0.01	0.49	0.60	0.58	− 0.01
SD	0.14	0.07	0.07		0.13	0.08	0.08	
SR35								
Mean	0.57	0.66	0.65	− 0.02	0.49	0.59	0.59	− 0.01
SD	0.14	0.07	0.07		0.14	0.08	0.08	
SR40								
Mean	0.56	0.65	0.64	− 0.02	0.49	0.59	0.58	− 0.01
SD	0.14	0.07	0.07		0.14	0.08	0.08	

TNC, Total Number of Children; CB, Child Before; MB, Married Before; and SR, Same Region

To assess the educational composition of the treated and never-treated regions in our design, we provide a comparison of the proportion of the population aged 16 or above with different educational attainment in treated and never-treated regions in 1970 as supplementary information (S3). 1970 is the closest to a baseline year for which full population local educational attainment data are available. Unsurprisingly, the treated regions in our design had slightly higher population-level baseline educational attainment than the untreated regions. It is important to note that our models do not account for potentially heterogeneous trajectories between regions with higher and lower population-level educational attainments at baseline.

**Fig. 2:**
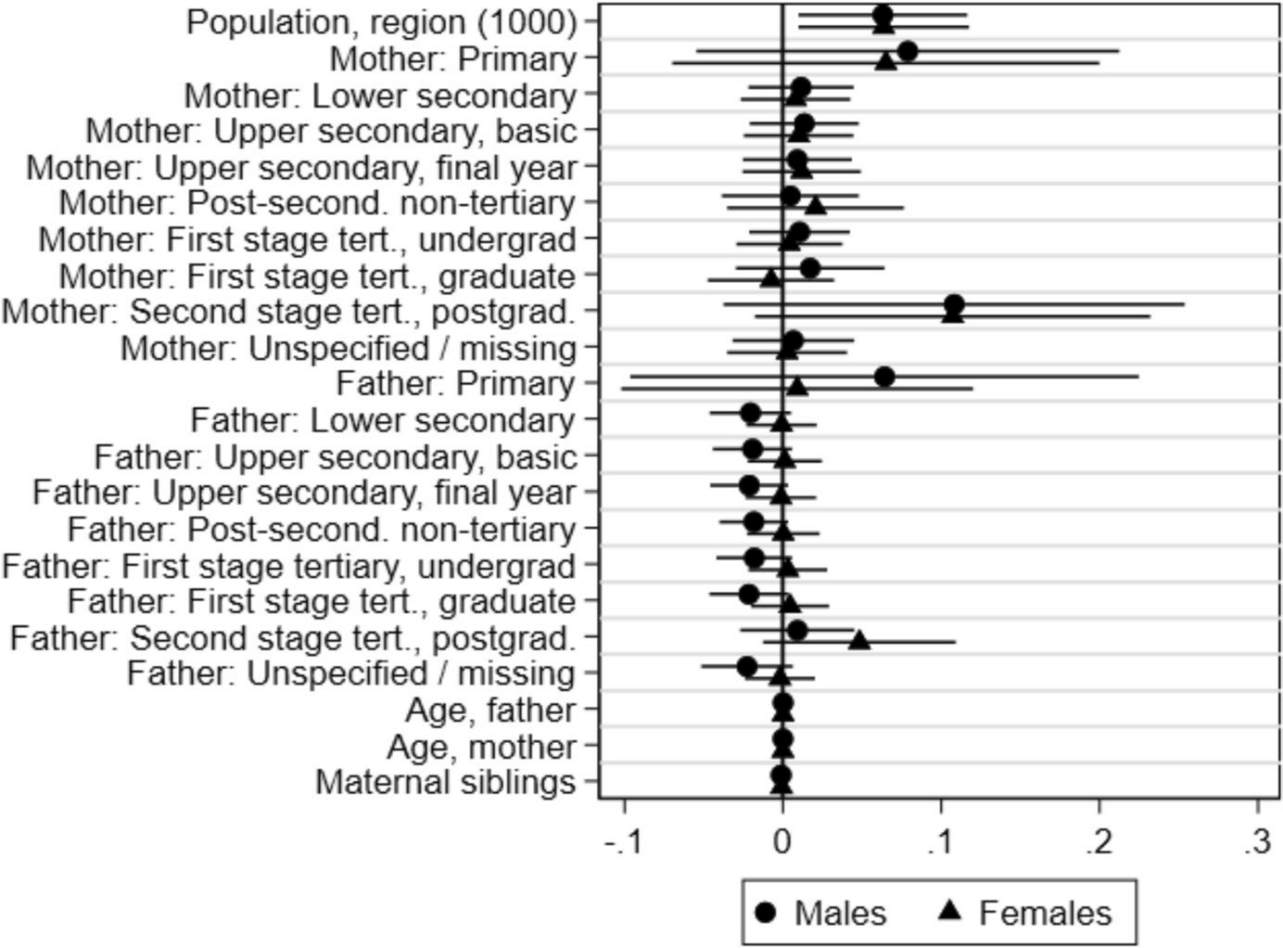
Balance tests. *Note*: Coefficients from two two-way fixed effects models (one for males and one for females) with 95% confidence intervals

As noted above, our identifying assumption only requires that the sample is conditionally balanced. Results of conditional balance tests, where our control variables are used to predict college establishment in a design with cohort and region fixed effects, are shown in Fig. 2. Reassuringly, only the population size in the region and no characteristics of the individuals were significant predictors of college establishments in this design.

### Effects on Fertility

#### Completed Family Size

**Fig. 3:**
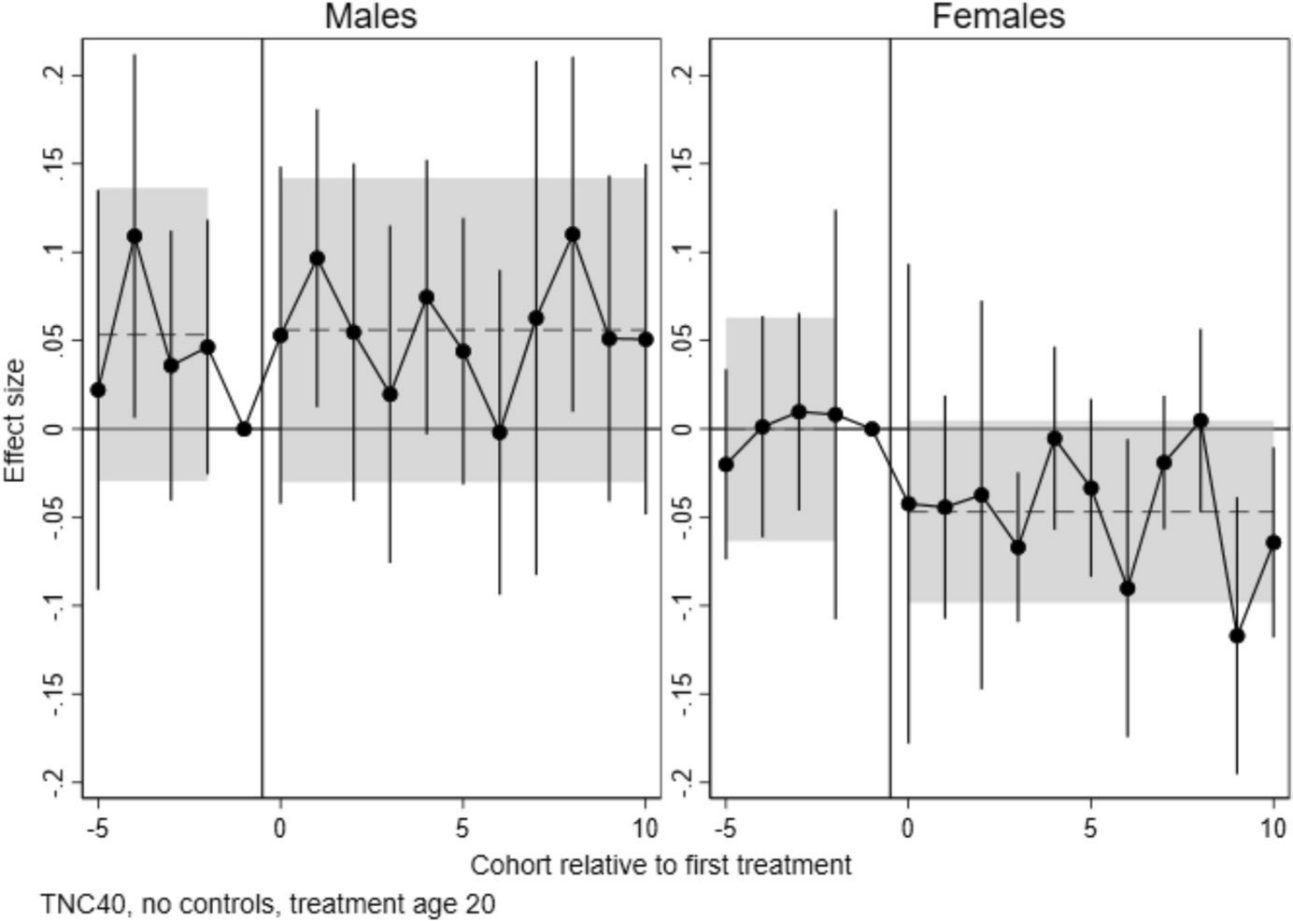
Event study estimates of the effects of local college establishments at age 20 on the total number of children born before age 40, by sex. *Note*: Points are coefficients for time dummies from difference-in-differences/event study models estimated with csdid, with 95% confidence intervals. Dashed lines represent the pre- and post-treatment average effects, with 95% confidence intervals shown as shaded areas

**Fig. 4:**
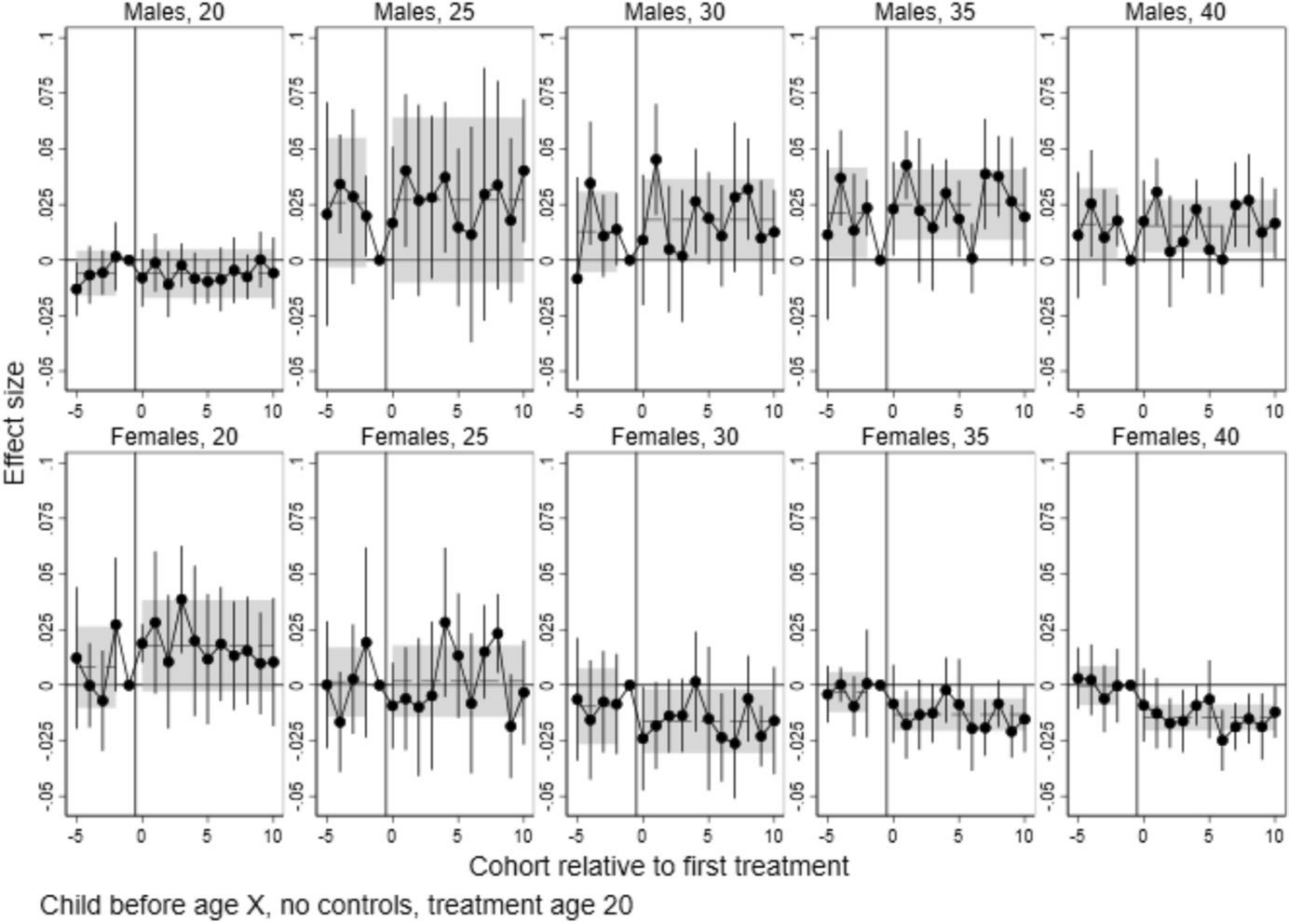
Event study estimates of the effects of local college establishments at age 20 on having children before age 20–40, by sex. *Note*: Points are coefficients for time dummies from difference-in-differences/event study models estimated with csdid, with 95% confidence intervals. Dashed lines represent the pre- and post-treatment average effects, with 95% confidence intervals shown as shaded areas

**Fig. 5:**
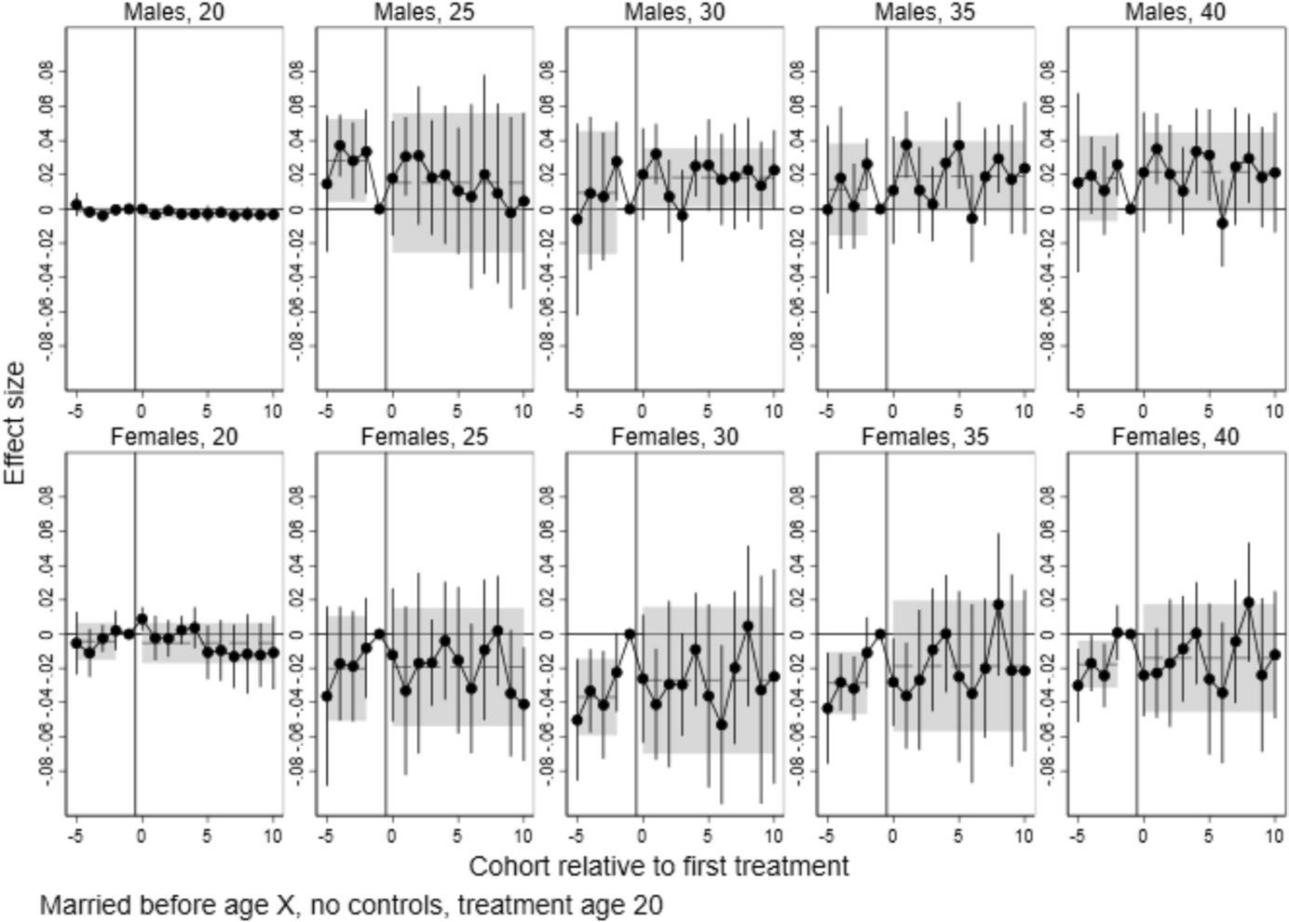
Event study estimates of the effects of local college establishments at age 20 on being married before age 20–40, by sex. *Note*: Points are coefficients for time dummies from difference-in-differences/event study models estimated with csdid, with 95% confidence intervals. Dashed lines represent the pre- and post-treatment average effects, with 95% confidence intervals shown as shaded areas

Event study estimates for the effect on number of children by age 40 are displayed in Fig. 3. Numerical results (coefficients and confidence intervals) for Figs. 3, 4, and 5 are provided as supplementary information (S4). In this main specification, college establishments are measured at age 20. First, it is worth noting that there is no firm evidence against the identifying assumption, as the pre-trend coefficients are not statistically significant. This holds both when tested separately for each pre-period cohort (dots show point estimates and spikes 95% CI), and jointly for all pre-period cohorts. (Dashed lines show point estimates and shaded areas show 95% CI.) However, we note that pre-treatment trends for men are somewhat erratic.

If college establishments affected fertility, we would see statistically significant estimates for the treated cohorts (zero or positive values of the duration variable). Our results show that coefficients are small (indicating an effect in the magnitude of less than 0.1 children) and mostly not statistically different from zero. For men, both pre-trends and effects tend to be above zero. For women, pre-trends are close to zero, and post-estimates are on average negative—albeit not statistically different from zero.

#### Effects on the Transition to Parenthood

Figure 4 shows the effects on the probability of having at least one child, estimated separately for ages 20, 25, 30, 35 and 40. If college establishments shifted births to an older age, we will see negative effect estimates at lower ages (20–30), with the effect waning towards age 40. It is worth noting that the pre-treatment trends for separate cohorts appear somewhat noisy, though they are rarely statistically significant. For men, joint pre-trends tend to be positive, albeit not statistically different from zero. For women, the point estimates are closer to zero both for the separate and joint pre-trends.

Looking at post-treatment estimates, different patterns emerge for men and women. For men, estimates are consistently positive, albeit noisy, and individual estimates are rarely statistically significant from zero. For ages 35 and 40, the joint post-estimate is statistically significant for men. However, we note that also the pre-trend hovers above zero, with a point estimate just marginally lower than the post-estimate. Thus, we are reluctant to give these results a causal interpretation.

For women, a negative and statistically significant effect emerges from age 30 and consolidates towards age 40. In other words, there is a slight increase in childlessness at age 40 among women for cohorts affected by college openings. This effect is in the magnitude of 1.5 percentage points. This equates to a 15 per cent increase in childlessness among women, relative to a baseline of 10 per cent childless women in the pre-period (Table 2). We return to the robustness of this finding to alternate specifications below.

### Effects on Marriage

Also in the absence of effects on fertility, it is possible that college expansions impacted partner market dynamics and thus the probability of marriage. Figure 5 shows results for the effects on the probability of being married before ages 20, 25, 30, 25 and 40. The results reveal several statistically significant estimates for the pre-treatment cohorts, indicating that the assumption of parallel trends does not hold. Four of the ten tests of joint pre-trends are statistically significant. Thus, these results should be interpreted with caution.

We also find no consistent effects on the probability of being married before different ages. For men, both pre-trends and post-estimates tend to hover above zero, as they do for the fertility outcomes. Except for age 30, the joint estimates are not statistically different from zero. As above, the similarity of the pre-trends and effects make us reluctant to give a causal interpretation to these patterns. For women, there is an increasing pre-trend for all age groups, significant from age 30 and onwards. In the post-period, the joint estimate is not statistically significant. Note that due to data limitations mentioned above, some pre-treatment cohorts are omitted from analyses of marriage before ages 20 and 25, making these less comparable.

### Effects on Retention and Population Size

**Fig. 6:**
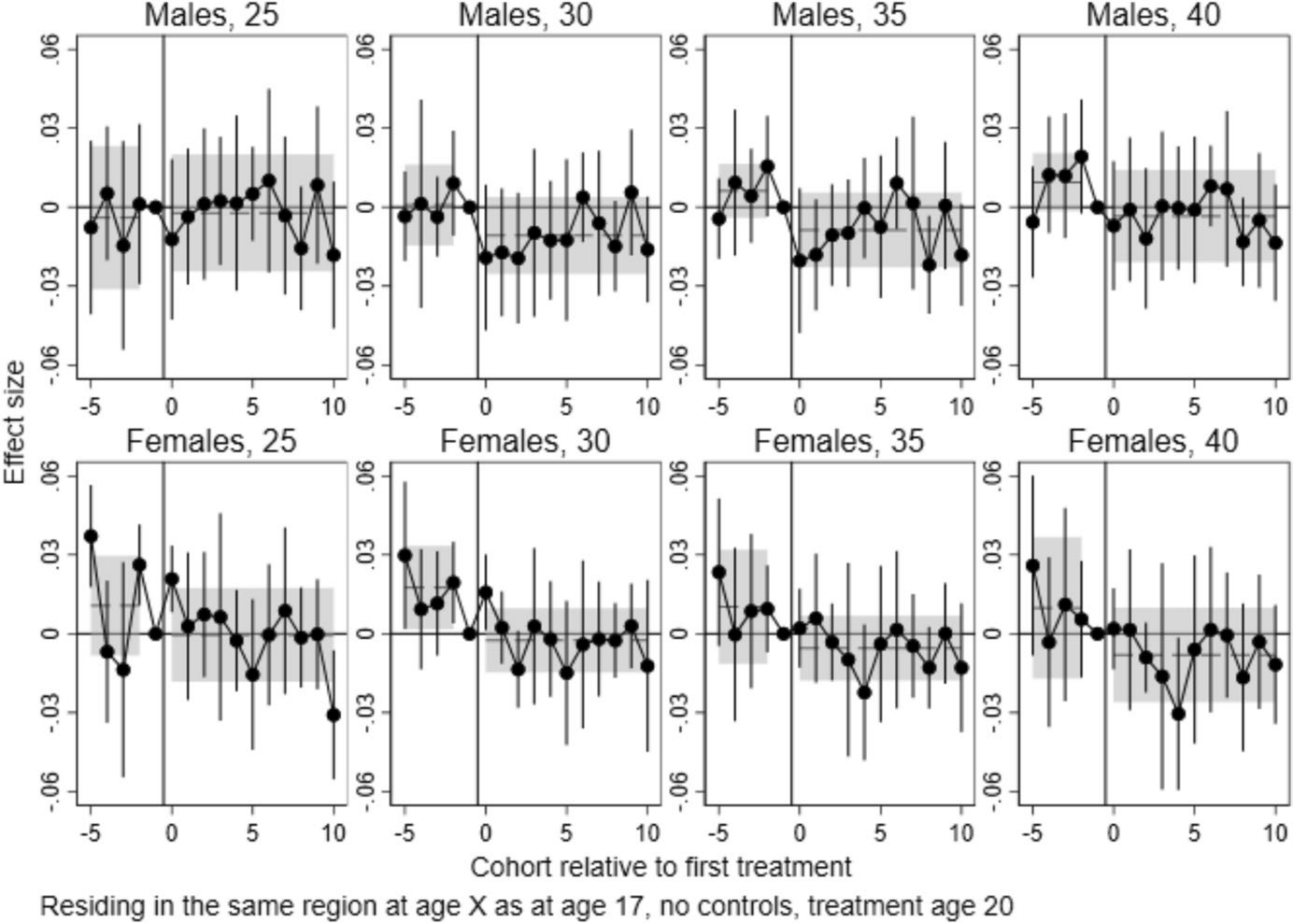
Event study estimates of the effects of local college establishments at age 20 on residing in the same region at ages 25–40, by sex. *Note*: Points are coefficients for time dummies from difference-in-differences/event study models estimated with csdid, with 95% confidence intervals. Dashed lines represent the pre- and post-treatment average effects, with 95% confidence intervals shown as shaded areas

Figure 6 shows the effects on retention in the region (whether individuals reside in the same region as they did at age 17). The pre-trends are generally statistically insignificant, and all joint pre-trends but one (women, age 30) are statistically similar to zero. The joint post-estimates suggest that local colleges do not substantively impact retention, neither for men nor women.

We have also estimated effects on population size in the regions (see below). However, as indicated by the balance tests, the pre-treatment trends in these analyses were not parallel, making it difficult to give these estimates a substantive interpretation.

### Robustness Tests

In addition to the balance tests described above, we performed several robustness tests.

First, we note that our sample is divided into quite fine-grained groups with one-year cohorts and small regions. Since the main results lack precision, this may raise a concern that we may be making an error of Type 2, i.e. that we are unable to detect effects of substantially meaningful sizes. We thus tested whether moving from one-year to two-year cohorts would improve precision (not shown). As these results were substantively similar to our main results, we retain the one-year cohorts that provide a more detailed and intuitive presentation of our results.

Second, we have tested whether effects are robust to changing the age at which we measure college availability at ages 18, 22 and 24, instead of age 20, which we use in our main specifications. Effectively, this robustness test shifts the reference category and treatment window: If treatment is measured at age 18, the previous first treated cohort is now in the pre-treatment group, defined as measured at *t*-2. Our overall finding of small and non-significant effects is generally robust to varying the treatment age (supplementary information S5, S6, S8). The results for childlessness at age 40 for women (S7) deserve a more detailed comment. Here, the main model showed a significant and quite substantial effect. When treatment is measured at age 18, so that the first treated cohorts from the main results are shifted into the pre-period, we find a positive pre-trend and no significant effects. This is as expected, given that the reference group is indeed treated. When treatment is shifted to age 22 and 24, the youngest cohorts in the treatment group are arguably only partly treated, as they were too old to fully benefit from the college reform. Thus, a zero effect on this group is unsurprising. In sum, these results do depend on the age at which treatment is measured. This does not in itself invalidate the main findings.

Third, we have estimated models where we include the control variables discussed above (S9-S12). Due to collinearity issues, these estimations could not produce pre-and post-treatment averages for all models. However, we still believe these estimates provide a valuable robustness check. Importantly, the effect of college expansions on women’s propensity to have a child before a given age (S11) is also netted out once these controls are included. This suggests that there are differences between the women in the reform and control region—rather than the reform itself—that drive the estimated effect in the main models. Otherwise, the robustness test yields results that are substantively similar to our main results. Fourth, we have tested using municipalities as the geographical unit, producing similar results (S13-S17). The negative effect on women’s fertility is robust to this specification (S15). Fifth, we have studied heterogeneity by running the main analyses only for individuals who have one or more parents with higher education. Again, the results were similar to our main results (not shown). Sixth, we have included controls for baseline educational levels, measured in 1970, in our main models (not shown). These models produced null results for almost all outcomes, but with some significant post-treatment estimates for age at marriage (small and positive for males above age 30; small and negative for females at age 20).

Seventh, we have compared our results to those obtained with other estimators, specifically did_multiplegt (De Chaisemartin et al., 2019) and a simpler, standard event study design, estimated as a difference-in-differences model with complete leads and lags. These approaches yield results that are substantively very similar to our main results (not shown). Eight, we have performed the strict test that all pre-treatment coefficients are zero (test statistics are provided in S18). This was rejected in all models, indicating that under this strict requirement, the pre-treatment trends in our models are not parallel. We note that this pattern emerges also in models where all individual pre-trend coefficients are statistically insignificant, and that this must be deemed a conservative test. As discussed in Sect. 4, there are some reasons to expect anticipatory effects in our design, and such effects would lead us to reject the null in this test. For these reasons, we are guided by the joint test of pre-trend coefficients, discussed in the presentation of results above, as our main empirical test of pre-trends. Deviations of pre-trends do not follow a systematic pattern across outcomes, or by pre-period.

Finally, while previous studies have shown that this reform did not impact educational attainment, nuances in modelling strategy may matter for results. Therefore, we show event study estimates for the effect of college establishments at age 20 on higher educational attainment in the supplementary material, S19 (see also S4, S5, S9 and S13). None of the post-treatment estimates are statistically significant among women, and they are substantially small among both men and women. The estimates are mostly positive among women and negative among men, echoing previous findings.

### Limitations

While our research design is intended to reduce the potential for biases by exploiting plausibly exogenous variation in college proximity, it still has important shortcomings. First, the nature of the reforms makes it difficult to exploit information on all higher educational institutions. Since many new colleges were simply upgraded from vocational schools, and the educations taken at such institutions provided similar opportunities and were registered as higher education both before and after the reforms, we have opted to treat all such institutions as colleges, rendering us with fewer ‘new’ institutions, more ‘always-treated’ regions and thus limited variation in college availability. It may be possible to improve on our research design by including more institutions, or exploiting the information gathered through this project on student capacity, also in always-treated regions, if an appropriate estimator is developed. As such, our local effects arise from medium sized cities in Norway (see Table 1), and the effect of establishing educational institutions in larger cities may or may not be the same. However, the external validity to other regional expansions, aiming to strengthen educational opportunities in regional centres and cities, is good. Relatedly, treated regions had a more highly educated population at baseline compared to never-treated regions. This may entail that their trajectories would have been different also in the absence of the reforms. However, as we do not observe treated regions post-reform in the absence of college establishments, this is not directly testable in our data.

Second, although local students were overrepresented at regional colleges, there was substantial spatial spillover. Many moved to other regions to obtain a college education, meaning that the reforms do not allow for a sharp identification of the effects of college access. Rather, our results reflect the effects of changes in college access within commuting distance, relative to regions further away. This also entails that the Stable Unit Treatment Value Assumption (SUTVA) (Angrist et al., 1996; Imbens & Rubin, 2015) is not met in our design, a common problem in studies relying on geographic variation for identification, that would bias our estimates towards zero. As in most designs relying on regional variation, it is also a concern that the college establishments may not have been strictly exogenous. It is reassuring that college establishments were not systematically related to characteristics of potential students in the regions aside from population size, but the possibility that they were related to unobserved characteristics can never be ruled out.

Third, this article is limited in scope and does not cover several relevant outcomes that may be affected by these reforms, such as choice of field, the quality of higher education, the characteristics of students or labour market outcomes. We encourage future research on these topics.

Finally, we cannot separate out the effects of college establishments per se, and the effects of more stringent admission requirements that accompanied these reforms, as we do not have full information on the admission requirements at individual institutions.

## Concluding Discussion

The expansion of the higher educational system, and improvements in educational opportunities, was one of the major societal changes that co-occurred with the reduction in fertility levels in the period studied here. The establishment of new educational institutions outside of the major cities was a central aspect of these reforms, especially in Norway. By studying the local impact of such establishments, we have attempted to provide evidence on how improvements in educational opportunities at the local level may have affected fertility patterns locally.

Our results generally indicate that the establishment of local colleges had little to no impact on the postponement of childbearing, total fertility, nor the propensity or timing of marriage for neither men nor women at the local level. In line with previous studies of the same reforms (Rogne et al., 2024, Knutsen et al., 2022), we found that the reforms had no effects on local educational attainment. Potentially, the localization of institutions may have affected *where* people studied rather than *whether* they studied and *who* studied. The increase in total student capacity generated by these reforms may have enabled more people to attain a higher education in general, but had little impact on the local population, relative to those in other regions.

Initially, we formed three main expectations of effects based on different mechanisms. Effects running through increases in educational attainment was not one of these, due to the known absence of effects from previous studies. Our first expectation was linked to changes in the local partner market. We could in part assess this mechanism empirically, through studying local migration retention, on which we found no effect. Our second expectation regarded local value changes as a channel for effects; these are notoriously hard to measure. Measuring effects through the labour market channel, our third expectation, fell outside the scope of this paper. A previous study suggested positive effects on skilled wages, which may increase opportunity costs to childbearing. It may of course be that these mechanisms were operative, and cancelled each other out, or that other counteracting mechanisms came into play. In total, however, our findings show that these college reforms did not impact on fertility, marriage or regional retention.

The finding that fertility is unaffected by local educational expansions in lieu of effects on educational attainment is of methodological importance. As discussed in Sect. 3.1, a number of other studies use local educational expansions college expansions as an instrumental variable (IV) for educational attainment, aiming to capture the causal effect of educational attainment on fertility. Our design can be considered an out-of-sample-test of indirect effects, where we test for effects on the outcome of interest in a sample where the there is no ‘first stage’ (cf. Cools and Hart, 2017). When our study finds no effects on fertility and family formation, this supports the notion that college expansions did not affect fertility and family formation through alternate channels. This, in turn, suggests that local college expansions that do affect educational attainment are a valid IV when applied to fertility and family formation as the outcome.

Using a rich register data set allowed us to make multiple robustness tests along several dimensions. We found that for population size, there were a significantly increasing trend in the reform regions (S20), starting before the reform and continuing afterwards. This suggests that the reform regions were on slightly different trajectories prior to the reform, continuing after the reform. These trends were not apparent in the joint pre-trends for the main outcomes. We handle the pre-trend by controlling for population size in the robustness tests. The effects on women’s fertility in the main models were explained by the inclusion of covariates. Without inclusion of these detailed controls, we may have erroneously concluded that local educational reforms reduced women’s fertility, through channels other than educational attainment. These findings underline that rich data and adequate methods have profound impacts on our understanding of demographic processes. If we were to use more basic or standard modelling strategies, such as including reform status as a covariate in a standard multivariate regression, the conclusion could again have been different. However, such strategies would be more prone to bias from unobservables (see, for example, Angrist & Pischke, 2009) and could thus yield misleading conclusions.

In conclusion, while our research design has limitations, we believe that our study makes a valuable contribution to understanding the impact of educational expansions on fertility and family formation in a critical period, as well as to the literature on educational reforms in general. The period we study is one of substantial societal change, where expanding educational opportunities and a marked decline in fertility to sub-replacement levels are important components. Our design allows us to test whether local changes in educational opportunities impacted fertility and family formation. Our results suggest that, at the local level, they did not.

## Supplementary Information

Below is the link to the electronic supplementary material.

Supplementary file1 (DOCX 3363 kb)

## Data Availability

The administrative register data used in this project were made available through the SEGREGATION project funded by the Research Council of Norway (RCN project 236793). These data are available from Statistics Norway to researchers with projects that satisfy the data owners’ requirements. For information on how to gain access to Norwegian microdata and formal requirements, see https://www.ssb.no/en/data-til-forskning/utlan-av-data-til-forskere. Data on educational institutions have been made available online, at https://osf.io/k8t6n/.

## Footnotes

1 Specifically, some institutions we study received college status some time after opening and can more precisely be characterized as ‘post-secondary, non-tertiary educational institutions’, but we refer to these colloquially as ‘colleges’.

2 Data quality is poor for 1993, so we focus on the period until 1992.

3 6 institutions that have received college status are classified as post-secondary, non-tertiary institutions throughout the period due to missing information on the timing of institutional upgrading. One institution received college status later.

4 We do not have full information on how admission requirements were practiced at different institutions in this period. But our overall impression from historical sources is that the introduction of high school diplomas as a prerequisite was gradual, and that some institutions were much more lenient than others. According to Brattvåg and Carlsson (1981: 3), 6.4% of new students at ‘district colleges’ did not have a 3-year high school education. But this share varied substantially between schools. (It was, for instance, 34.6% at Nordland Distriktshøgskole.)

5 Empirically assessing the impacts of such reclassifications on labour market outcomes is a relevant research topic, but it falls beyond the scope of the current paper.

6 Looking at moves between ages 15 and 17, fewer moved in the never-treated regions, but we find insubstantial differences in treated regions before and after the college establishments.

7 It is common in Norway to take one or more ‘break years’ after high school graduation, which may also include attendance at informal ‘folk high schools’.

8 The value 0 includes people who have either moved, died or emigrated, as separating between these outcomes would require us to condition on a post-treatment variable.

9 In supplementary analyses, we also include a set of control variables (**X**), described below, all measured prior to the treatment. These models can be expressed as:

$${y}_{i}=\sum_{k=-4}^{10}{\beta }_{k}{D}_{i}^{k}+{\theta }_{c}+{\theta }_{r}+{\varvec{\beta}}\mathbf{X}+ {\varepsilon }_{i}$$
