# Supplementary material for: Educational Expansions and Fertility: Evidence from Norwegian College Reforms
Source: Eur J Popul. 2025 Jun 2;41(1):14. doi: 10.1007/s10680-025-09737-7 (PMC12130375; doi:10.1007/s10680-025-09737-7)
Supplement: Supplementary file 1 — Supplementary file1 (DOCX 3363 kb) [file 10680_2025_9737_MOESM1_ESM.docx]

***Supplementary information***

**Educational expansions and fertility:**

**Evidence from Norwegian college reforms**

**S1: Descriptive statistics for control variables**

|  | Males |  |  |  | Females |  |  |  |
| --- | --- | --- | --- | --- | --- | --- | --- | --- |
|  | Never | Pre | Post | Diff | Never | Pre | Post | Diff |
| Population size |  |  |  |  |  |  |  |  |
| Mean | 26419.70 | 49519.67 | 50606.86 | 1087.19 | 26540.97 | 49850.79 | 50475.60 | 624.81 |
| SD | 16849.12 | 23241.87 | 25505.49 |  | 17016.59 | 23539.06 | 25374.32 |  |
| Mother, no/pre-school ed. |  |  |  |  |  |  |  |  |
| Mean | 0.00 | 0.00 | 0.00 | 0.00 | 0.00 | 0.00 | 0.00 | 0.00 |
| SD | 0.00 | 0.00 | 0.00 |  | 0.00 | 0.00 | 0.00 |  |
| Mother, primary ed. |  |  |  |  |  |  |  |  |
| Mean | 0.00 | 0.00 | 0.00 | 0.00 | 0.00 | 0.00 | 0.00 | 0.00 |
| SD | 0.00 | 0.00 | 0.00 |  | 0.00 | 0.00 | 0.00 |  |
| Mother, lower second. ed. |  |  |  |  |  |  |  |  |
| Mean | 0.52 | 0.57 | 0.47 | -0.11 | 0.51 | 0.57 | 0.47 | -0.10 |
| SD | 0.14 | 0.06 | 0.09 |  | 0.13 | 0.06 | 0.08 |  |
| Mother, upper second. ed., basic |  |  |  |  |  |  |  |  |
| Mean | 0.37 | 0.33 | 0.41 | 0.08 | 0.37 | 0.33 | 0.41 | 0.08 |
| SD | 0.11 | 0.06 | 0.07 |  | 0.10 | 0.06 | 0.06 |  |
| Mother, upper second., final year |  |  |  |  |  |  |  |  |
| Mean | 0.03 | 0.03 | 0.03 | 0.01 | 0.03 | 0.02 | 0.03 | 0.01 |
| SD | 0.02 | 0.01 | 0.02 |  | 0.02 | 0.01 | 0.02 |  |
| Mother, post-second. non-tert. ed. |  |  |  |  |  |  |  |  |
| Mean | 0.00 | 0.00 | 0.00 | 0.00 | 0.00 | 0.00 | 0.01 | 0.00 |
| SD | 0.01 | 0.00 | 0.00 |  | 0.01 | 0.00 | 0.01 |  |
| Mother, first stage tert. ed., undergrad. |  |  |  |  |  |  |  |  |
| Mean | 0.06 | 0.04 | 0.07 | 0.03 | 0.06 | 0.04 | 0.07 | 0.03 |
| SD | 0.04 | 0.02 | 0.03 |  | 0.04 | 0.01 | 0.02 |  |
| Mother, first stage tert. ed., grad. |  |  |  |  |  |  |  |  |
| Mean | 0.00 | 0.00 | 0.00 | 0.00 | 0.00 | 0.00 | 0.00 | 0.00 |
| SD | 0.00 | 0.00 | 0.00 |  | 0.00 | 0.00 | 0.00 |  |
| Mother, second stage tert. ed., postgrad. |  |  |  |  |  |  |  |  |
| Mean | 0.00 | 0.00 | 0.00 | 0.00 | 0.00 | 0.00 | 0.00 | 0.00 |
| SD | 0.00 | 0.00 | 0.00 |  | 0.00 | 0.00 | 0.00 |  |
| Mother, unspecified/missing ed. |  |  |  |  |  |  |  |  |
| Mean | 0.02 | 0.02 | 0.01 | -0.01 | 0.03 | 0.03 | 0.01 | -0.02 |
| SD | 0.02 | 0.02 | 0.01 |  | 0.05 | 0.05 | 0.01 |  |
| Father, no/pre-school ed. |  |  |  |  |  |  |  |  |
| Mean | 0.00 | 0.00 | 0.00 | 0.00 | 0.00 | 0.00 | 0.00 | 0.00 |
| SD | 0.00 | 0.00 | 0.00 |  | 0.00 | 0.00 | 0.00 |  |
| Father, primary ed. |  |  |  |  |  |  |  |  |
| Mean | 0.00 | 0.00 | 0.00 | 0.00 | 0.00 | 0.00 | 0.00 | 0.00 |
| SD | 0.00 | 0.00 | 0.00 |  | 0.00 | 0.00 | 0.00 |  |
| Father, lower second. ed. |  |  |  |  |  |  |  |  |
| Mean | 0.45 | 0.45 | 0.38 | -0.07 | 0.44 | 0.44 | 0.38 | -0.06 |
| SD | 0.11 | 0.05 | 0.08 |  | 0.10 | 0.05 | 0.08 |  |
| Father, upper second. ed., basic |  |  |  |  |  |  |  |  |
| Mean | 0.31 | 0.30 | 0.34 | 0.04 | 0.30 | 0.30 | 0.33 | 0.04 |
| SD | 0.07 | 0.05 | 0.06 |  | 0.07 | 0.06 | 0.06 |  |
| Father, upper second., final year |  |  |  |  |  |  |  |  |
| Mean | 0.10 | 0.10 | 0.11 | 0.02 | 0.09 | 0.10 | 0.11 | 0.02 |
| SD | 0.05 | 0.04 | 0.04 |  | 0.05 | 0.04 | 0.05 |  |
| Father, post-second. non-tert. ed. |  |  |  |  |  |  |  |  |
| Mean | 0.02 | 0.02 | 0.02 | 0.01 | 0.02 | 0.02 | 0.02 | 0.01 |
| SD | 0.02 | 0.01 | 0.02 |  | 0.02 | 0.02 | 0.02 |  |
| Father, first stage tert. ed., undergrad. |  |  |  |  |  |  |  |  |
| Mean | 0.06 | 0.05 | 0.08 | 0.03 | 0.06 | 0.05 | 0.08 | 0.03 |
| SD | 0.04 | 0.02 | 0.03 |  | 0.04 | 0.02 | 0.03 |  |
| Father, first stage tert. ed., grad. |  |  |  |  |  |  |  |  |
| Mean | 0.02 | 0.03 | 0.03 | 0.00 | 0.02 | 0.02 | 0.03 | 0.01 |
| SD | 0.02 | 0.01 | 0.01 |  | 0.02 | 0.01 | 0.02 |  |
| Father, second stage tert. ed., postgrad. |  |  |  |  |  |  |  |  |
| Mean | 0.00 | 0.00 | 0.00 | 0.00 | 0.00 | 0.00 | 0.00 | 0.00 |
| SD | 0.00 | 0.00 | 0.00 |  | 0.00 | 0.00 | 0.00 |  |
| Father, unspecified/missing ed. |  |  |  |  |  |  |  |  |
| Mean | 0.04 | 0.06 | 0.03 | -0.02 | 0.06 | 0.07 | 0.03 | -0.04 |
| SD | 0.04 | 0.03 | 0.02 |  | 0.07 | 0.06 | 0.02 |  |
| Age, father |  |  |  |  |  |  |  |  |
| Mean | 48.69 | 49.73 | 48.20 | -1.53 | 48.69 | 49.70 | 48.16 | -1.54 |
| SD | 1.93 | 1.23 | 1.67 |  | 1.93 | 1.32 | 1.62 |  |
| Age, mother |  |  |  |  |  |  |  |  |
| Mean | 44.84 | 45.91 | 44.51 | -1.40 | 44.86 | 45.92 | 44.47 | -1.45 |
| SD | 1.57 | 0.98 | 1.31 |  | 1.59 | 0.95 | 1.21 |  |
| Maternal siblings (incl. ego) |  |  |  |  |  |  |  |  |
| Mean | 3.27 | 3.42 | 3.43 | 0.01 | 3.27 | 3.45 | 3.46 | 0.01 |
| SD | 0.39 | 0.32 | 0.40 |  | 0.39 | 0.33 | 0.43 |  |

**S2: Trends in control variables and outcome variables in treated and never-treated regions**

*
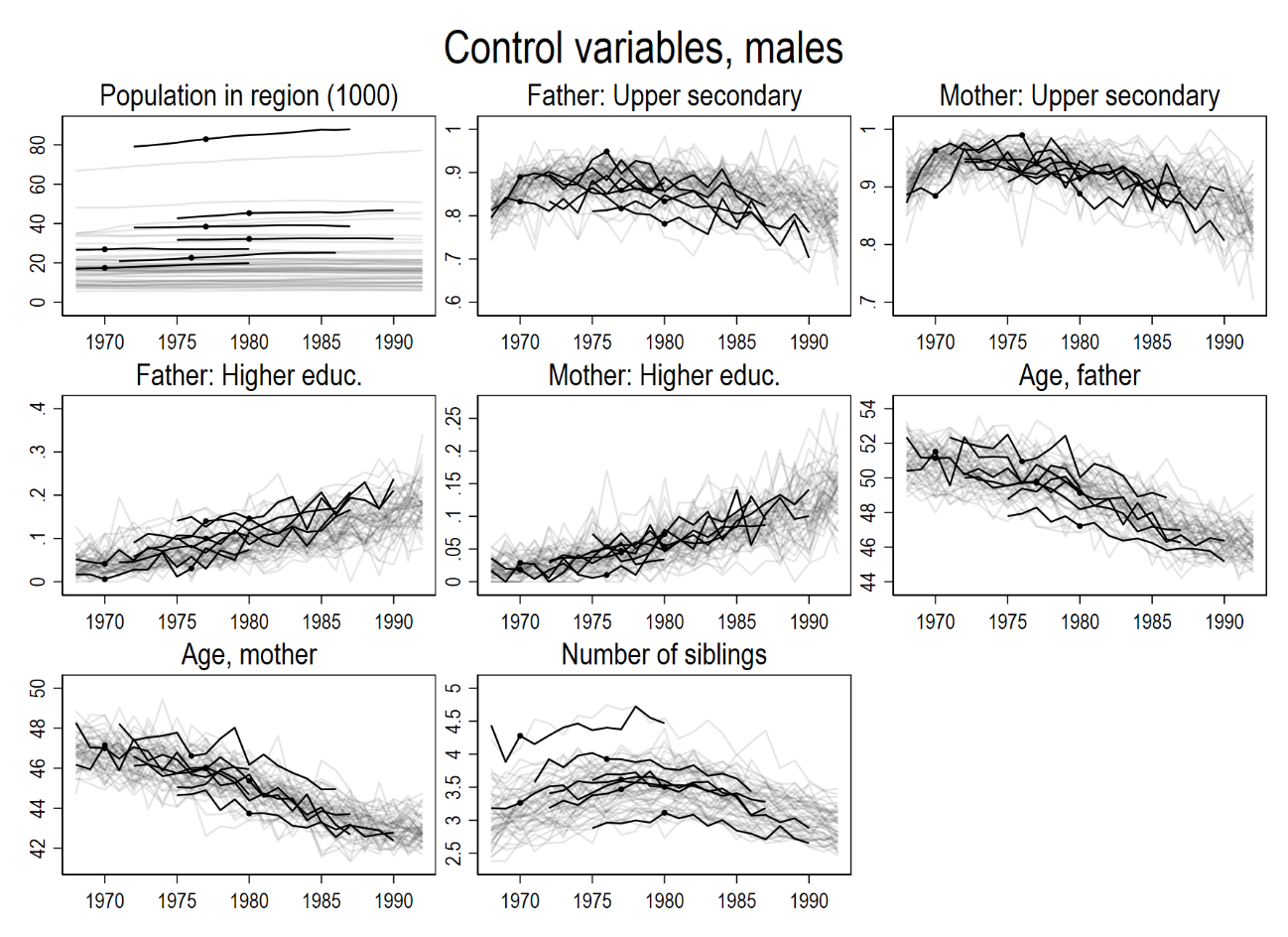
*


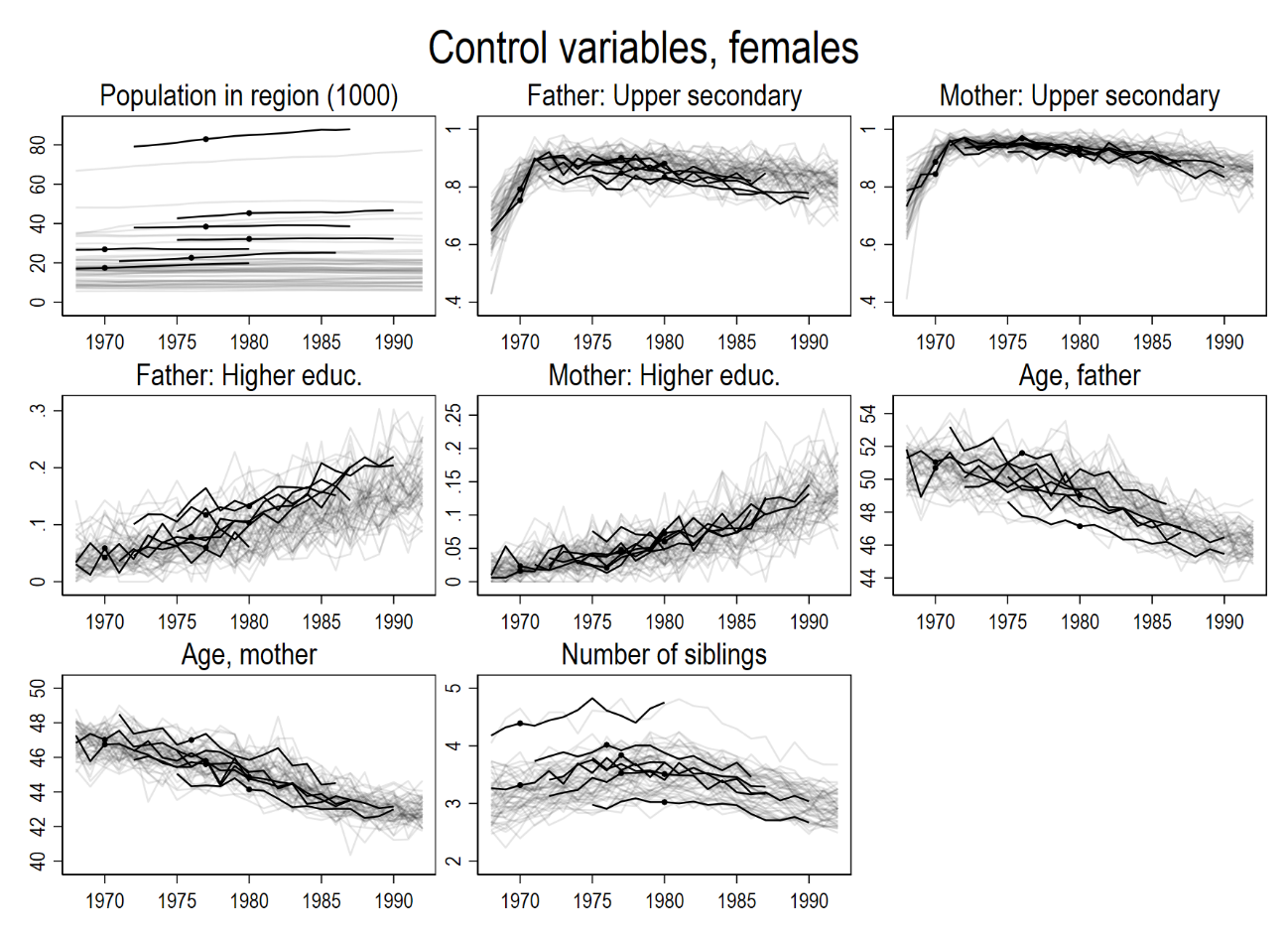


***
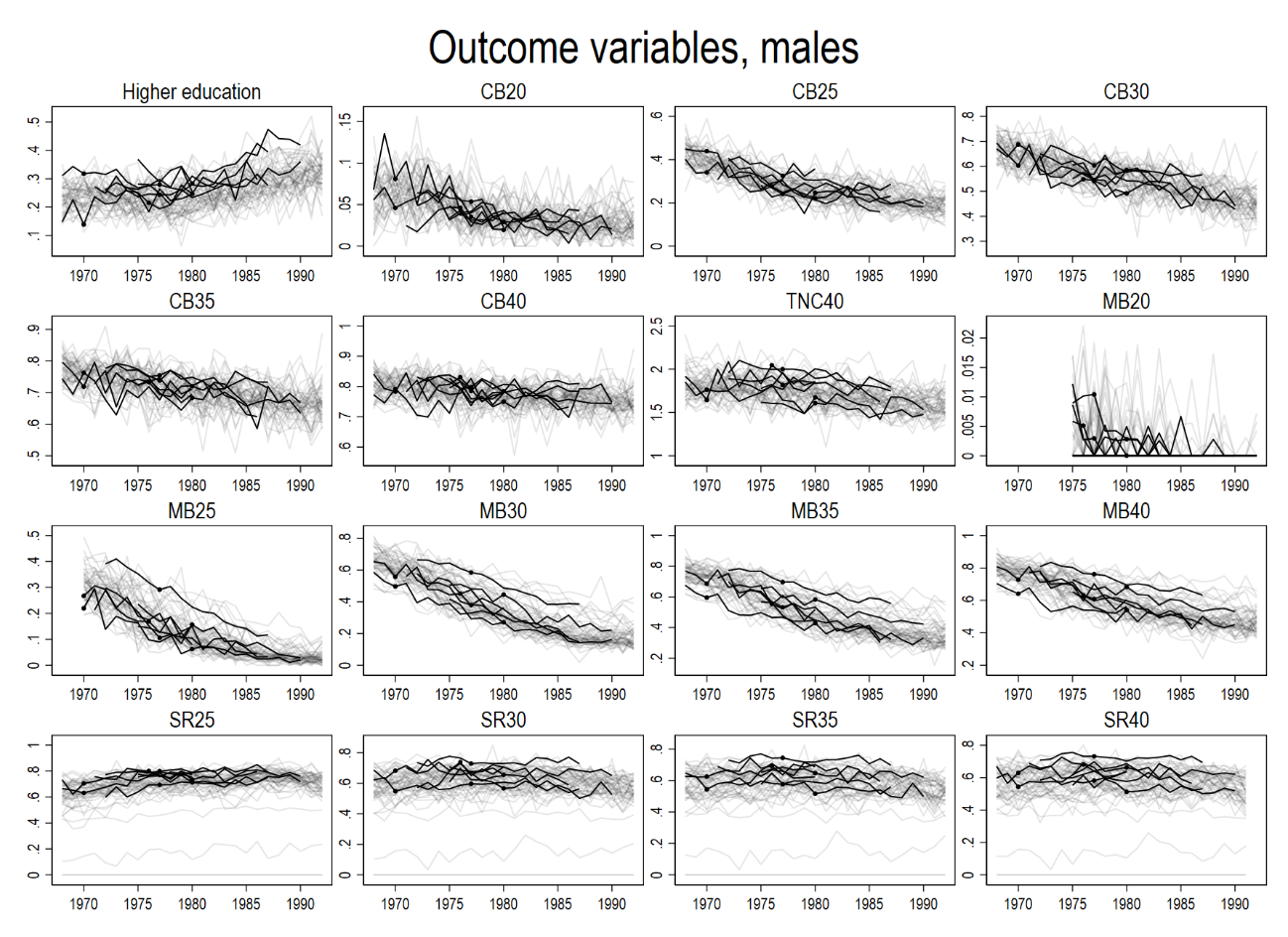
***

***
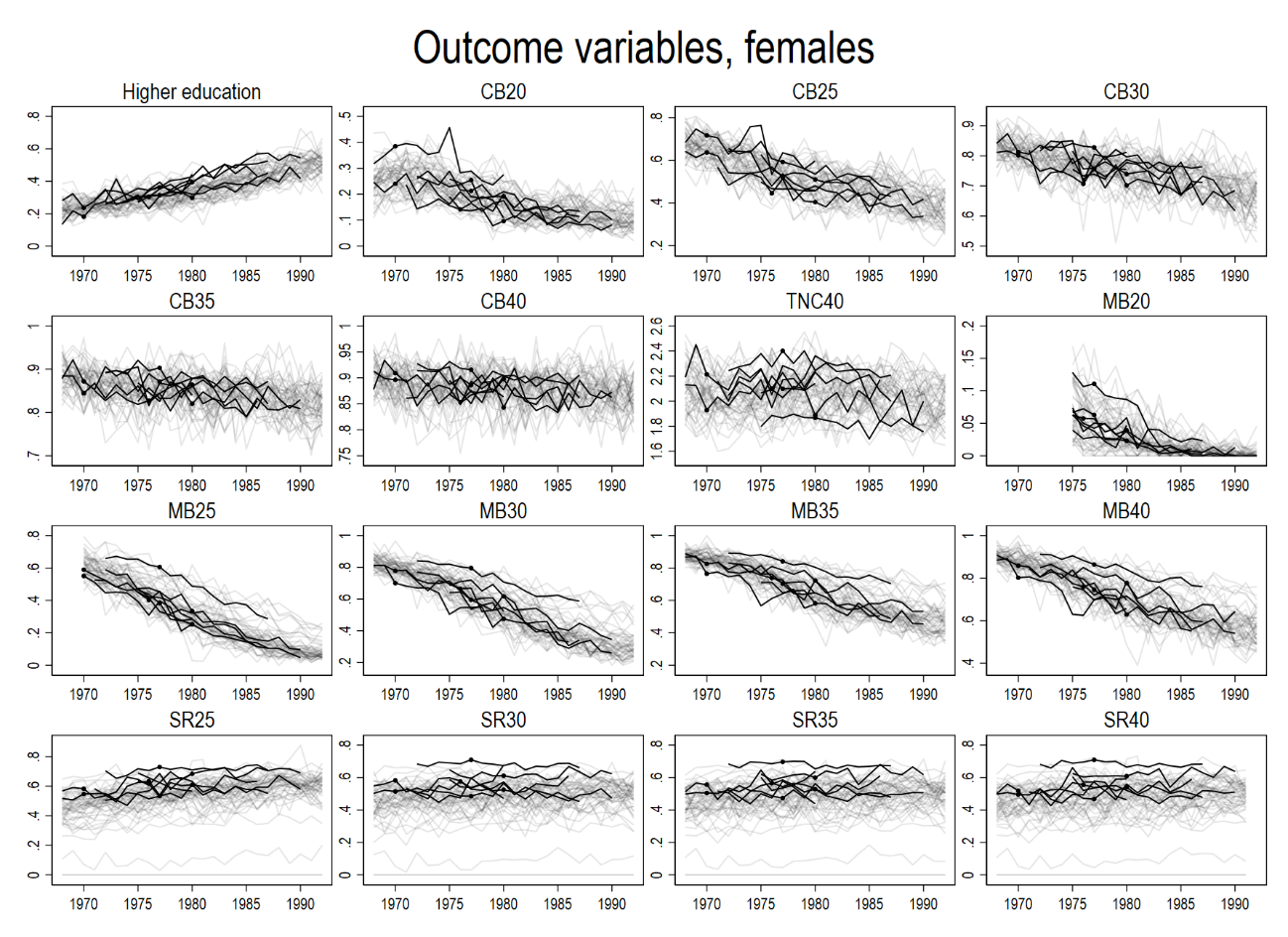
***

*Note: Treated regions are shown as black lines with a dot marking the treatment year. Never-treated regions are shown as grey lines. Years are shifted by 3 due to the measurement of treatment at age 20, for individuals resident at age 17. Educational attainment is included among outcome variables as it is relevant to show descriptively although it is not included in the analyses. Parental education is grouped to simplify the visual presentation. Number of siblings refers to maternal siblings. Abbreviations: CB – Child Before; TNC – Total Number of Children; MB – Married Before; SR – Same Region.*

**S3: Comparison of baseline population-level education in treated and never-treated regions in 1970**

| Treatment group |  | Never-treated, % | Treated, % |
| --- | --- | --- | --- |
| Basic school level |  | 59.23 | 54.15 |
| Upper secondary education |  | 35.61 | 39.60 |
| Higher education, short |  | 3.77 | 4.40 |
| Higher education, long |  | 0.78 | 1.07 |
| Unknown or no completed education |  | 0.60 | 0.78 |
| N regions |  | 49 | 7 |
| N individuals |  | 626 207 | 167 576 |

*Note: This table is based on official statistics for the entire population in the region aged 16 and older in 1970, and not subject to the sample restrictions in our analyses. Percentages are weighted by the population aged 16 and older in the region. Always-treated regions are not included. Source: https://www.ssb.no/statbank/table/09429*

**S4: Coefficients and confidence intervals for Figures 3-6**

| Outcome | Sex | Time | Coef. | Lower CI | Upper CI | Avg. coef. | Avg. lower CI | Avg. upper CI | DF | Chi2 | P |
| --- | --- | --- | --- | --- | --- | --- | --- | --- | --- | --- | --- |
| TNC40 | Male | -5 | 0.022 | -0.091 | 0.135 | 0.053 | -0.030 | 0.136 | 13 | 13446.391 | 0.000 |
| TNC40 | Male | -4 | 0.109 | 0.006 | 0.212 | 0.053 | -0.030 | 0.136 | 13 | 13446.391 | 0.000 |
| TNC40 | Male | -3 | 0.036 | -0.040 | 0.112 | 0.053 | -0.030 | 0.136 | 13 | 13446.391 | 0.000 |
| TNC40 | Male | -2 | 0.046 | -0.026 | 0.118 | 0.053 | -0.030 | 0.136 | 13 | 13446.391 | 0.000 |
| TNC40 | Male | -1 | 0.000 | 0.000 | 0.000 |  |  |  | 13 | 13446.391 | 0.000 |
| TNC40 | Male | 0 | 0.053 | -0.042 | 0.148 | 0.056 | -0.030 | 0.142 | 13 | 13446.391 | 0.000 |
| TNC40 | Male | 1 | 0.097 | 0.012 | 0.181 | 0.056 | -0.030 | 0.142 | 13 | 13446.391 | 0.000 |
| TNC40 | Male | 2 | 0.055 | -0.041 | 0.150 | 0.056 | -0.030 | 0.142 | 13 | 13446.391 | 0.000 |
| TNC40 | Male | 3 | 0.020 | -0.076 | 0.115 | 0.056 | -0.030 | 0.142 | 13 | 13446.391 | 0.000 |
| TNC40 | Male | 4 | 0.075 | -0.003 | 0.152 | 0.056 | -0.030 | 0.142 | 13 | 13446.391 | 0.000 |
| TNC40 | Male | 5 | 0.044 | -0.031 | 0.119 | 0.056 | -0.030 | 0.142 | 13 | 13446.391 | 0.000 |
| TNC40 | Male | 6 | -0.002 | -0.094 | 0.090 | 0.056 | -0.030 | 0.142 | 13 | 13446.391 | 0.000 |
| TNC40 | Male | 7 | 0.063 | -0.083 | 0.208 | 0.056 | -0.030 | 0.142 | 13 | 13446.391 | 0.000 |
| TNC40 | Male | 8 | 0.110 | 0.010 | 0.211 | 0.056 | -0.030 | 0.142 | 13 | 13446.391 | 0.000 |
| TNC40 | Male | 9 | 0.051 | -0.041 | 0.143 | 0.056 | -0.030 | 0.142 | 13 | 13446.391 | 0.000 |
| TNC40 | Male | 10 | 0.051 | -0.048 | 0.150 | 0.056 | -0.030 | 0.142 | 13 | 13446.391 | 0.000 |
| TNC40 | Female | -5 | -0.020 | -0.074 | 0.034 | 0.000 | -0.063 | 0.063 | 13 | 1150.662 | 0.000 |
| TNC40 | Female | -4 | 0.001 | -0.061 | 0.064 | 0.000 | -0.063 | 0.063 | 13 | 1150.662 | 0.000 |
| TNC40 | Female | -3 | 0.010 | -0.046 | 0.066 | 0.000 | -0.063 | 0.063 | 13 | 1150.662 | 0.000 |
| TNC40 | Female | -2 | 0.008 | -0.108 | 0.124 | 0.000 | -0.063 | 0.063 | 13 | 1150.662 | 0.000 |
| TNC40 | Female | -1 | 0.000 | 0.000 | 0.000 |  |  |  | 13 | 1150.662 | 0.000 |
| TNC40 | Female | 0 | -0.042 | -0.178 | 0.093 | -0.047 | -0.098 | 0.005 | 13 | 1150.662 | 0.000 |
| TNC40 | Female | 1 | -0.044 | -0.107 | 0.019 | -0.047 | -0.098 | 0.005 | 13 | 1150.662 | 0.000 |
| TNC40 | Female | 2 | -0.037 | -0.147 | 0.073 | -0.047 | -0.098 | 0.005 | 13 | 1150.662 | 0.000 |
| TNC40 | Female | 3 | -0.067 | -0.109 | -0.025 | -0.047 | -0.098 | 0.005 | 13 | 1150.662 | 0.000 |
| TNC40 | Female | 4 | -0.005 | -0.057 | 0.047 | -0.047 | -0.098 | 0.005 | 13 | 1150.662 | 0.000 |
| TNC40 | Female | 5 | -0.033 | -0.084 | 0.017 | -0.047 | -0.098 | 0.005 | 13 | 1150.662 | 0.000 |
| TNC40 | Female | 6 | -0.090 | -0.174 | -0.006 | -0.047 | -0.098 | 0.005 | 13 | 1150.662 | 0.000 |
| TNC40 | Female | 7 | -0.019 | -0.057 | 0.019 | -0.047 | -0.098 | 0.005 | 13 | 1150.662 | 0.000 |
| TNC40 | Female | 8 | 0.005 | -0.047 | 0.057 | -0.047 | -0.098 | 0.005 | 13 | 1150.662 | 0.000 |
| TNC40 | Female | 9 | -0.117 | -0.195 | -0.039 | -0.047 | -0.098 | 0.005 | 13 | 1150.662 | 0.000 |
| TNC40 | Female | 10 | -0.064 | -0.118 | -0.010 | -0.047 | -0.098 | 0.005 | 13 | 1150.662 | 0.000 |
| CB20 | Male | -5 | -0.013 | -0.025 | -0.001 | -0.006 | -0.016 | 0.004 | 13 | 545.201 | 0.000 |
| CB20 | Male | -4 | -0.007 | -0.020 | 0.006 | -0.006 | -0.016 | 0.004 | 13 | 545.201 | 0.000 |
| CB20 | Male | -3 | -0.006 | -0.016 | 0.004 | -0.006 | -0.016 | 0.004 | 13 | 545.201 | 0.000 |
| CB20 | Male | -2 | 0.002 | -0.014 | 0.017 | -0.006 | -0.016 | 0.004 | 13 | 545.201 | 0.000 |
| CB20 | Male | -1 | 0.000 | 0.000 | 0.000 |  |  |  | 13 | 545.201 | 0.000 |
| CB20 | Male | 0 | -0.008 | -0.021 | 0.005 | -0.006 | -0.017 | 0.005 | 13 | 545.201 | 0.000 |
| CB20 | Male | 1 | -0.001 | -0.014 | 0.012 | -0.006 | -0.017 | 0.005 | 13 | 545.201 | 0.000 |
| CB20 | Male | 2 | -0.011 | -0.026 | 0.004 | -0.006 | -0.017 | 0.005 | 13 | 545.201 | 0.000 |
| CB20 | Male | 3 | -0.002 | -0.012 | 0.007 | -0.006 | -0.017 | 0.005 | 13 | 545.201 | 0.000 |
| CB20 | Male | 4 | -0.008 | -0.020 | 0.003 | -0.006 | -0.017 | 0.005 | 13 | 545.201 | 0.000 |
| CB20 | Male | 5 | -0.010 | -0.020 | 0.000 | -0.006 | -0.017 | 0.005 | 13 | 545.201 | 0.000 |
| CB20 | Male | 6 | -0.009 | -0.023 | 0.006 | -0.006 | -0.017 | 0.005 | 13 | 545.201 | 0.000 |
| CB20 | Male | 7 | -0.005 | -0.019 | 0.010 | -0.006 | -0.017 | 0.005 | 13 | 545.201 | 0.000 |
| CB20 | Male | 8 | -0.008 | -0.018 | 0.002 | -0.006 | -0.017 | 0.005 | 13 | 545.201 | 0.000 |
| CB20 | Male | 9 | 0.000 | -0.012 | 0.013 | -0.006 | -0.017 | 0.005 | 13 | 545.201 | 0.000 |
| CB20 | Male | 10 | -0.006 | -0.022 | 0.010 | -0.006 | -0.017 | 0.005 | 13 | 545.201 | 0.000 |
| CB20 | Female | -5 | 0.012 | -0.020 | 0.044 | 0.008 | -0.010 | 0.026 | 13 | 13684.719 | 0.000 |
| CB20 | Female | -4 | 0.000 | -0.019 | 0.019 | 0.008 | -0.010 | 0.026 | 13 | 13684.719 | 0.000 |
| CB20 | Female | -3 | -0.007 | -0.030 | 0.016 | 0.008 | -0.010 | 0.026 | 13 | 13684.719 | 0.000 |
| CB20 | Female | -2 | 0.027 | -0.003 | 0.057 | 0.008 | -0.010 | 0.026 | 13 | 13684.719 | 0.000 |
| CB20 | Female | -1 | 0.000 | 0.000 | 0.000 |  |  |  | 13 | 13684.719 | 0.000 |
| CB20 | Female | 0 | 0.019 | 0.010 | 0.027 | 0.018 | -0.003 | 0.038 | 13 | 13684.719 | 0.000 |
| CB20 | Female | 1 | 0.028 | -0.004 | 0.060 | 0.018 | -0.003 | 0.038 | 13 | 13684.719 | 0.000 |
| CB20 | Female | 2 | 0.010 | -0.020 | 0.041 | 0.018 | -0.003 | 0.038 | 13 | 13684.719 | 0.000 |
| CB20 | Female | 3 | 0.039 | 0.014 | 0.063 | 0.018 | -0.003 | 0.038 | 13 | 13684.719 | 0.000 |
| CB20 | Female | 4 | 0.020 | -0.014 | 0.054 | 0.018 | -0.003 | 0.038 | 13 | 13684.719 | 0.000 |
| CB20 | Female | 5 | 0.012 | -0.018 | 0.041 | 0.018 | -0.003 | 0.038 | 13 | 13684.719 | 0.000 |
| CB20 | Female | 6 | 0.019 | -0.007 | 0.044 | 0.018 | -0.003 | 0.038 | 13 | 13684.719 | 0.000 |
| CB20 | Female | 7 | 0.013 | -0.011 | 0.038 | 0.018 | -0.003 | 0.038 | 13 | 13684.719 | 0.000 |
| CB20 | Female | 8 | 0.016 | -0.009 | 0.040 | 0.018 | -0.003 | 0.038 | 13 | 13684.719 | 0.000 |
| CB20 | Female | 9 | 0.010 | -0.013 | 0.033 | 0.018 | -0.003 | 0.038 | 13 | 13684.719 | 0.000 |
| CB20 | Female | 10 | 0.010 | -0.018 | 0.039 | 0.018 | -0.003 | 0.038 | 13 | 13684.719 | 0.000 |
| CB25 | Male | -5 | 0.021 | -0.030 | 0.071 | 0.026 | -0.003 | 0.055 | 13 | 12272.925 | 0.000 |
| CB25 | Male | -4 | 0.034 | 0.012 | 0.056 | 0.026 | -0.003 | 0.055 | 13 | 12272.925 | 0.000 |
| CB25 | Male | -3 | 0.029 | -0.011 | 0.068 | 0.026 | -0.003 | 0.055 | 13 | 12272.925 | 0.000 |
| CB25 | Male | -2 | 0.020 | 0.002 | 0.038 | 0.026 | -0.003 | 0.055 | 13 | 12272.925 | 0.000 |
| CB25 | Male | -1 | 0.000 | 0.000 | 0.000 |  |  |  | 13 | 12272.925 | 0.000 |
| CB25 | Male | 0 | 0.017 | -0.018 | 0.051 | 0.027 | -0.010 | 0.064 | 13 | 12272.925 | 0.000 |
| CB25 | Male | 1 | 0.040 | 0.006 | 0.075 | 0.027 | -0.010 | 0.064 | 13 | 12272.925 | 0.000 |
| CB25 | Male | 2 | 0.027 | -0.016 | 0.070 | 0.027 | -0.010 | 0.064 | 13 | 12272.925 | 0.000 |
| CB25 | Male | 3 | 0.028 | -0.008 | 0.065 | 0.027 | -0.010 | 0.064 | 13 | 12272.925 | 0.000 |
| CB25 | Male | 4 | 0.037 | 0.003 | 0.071 | 0.027 | -0.010 | 0.064 | 13 | 12272.925 | 0.000 |
| CB25 | Male | 5 | 0.015 | -0.021 | 0.050 | 0.027 | -0.010 | 0.064 | 13 | 12272.925 | 0.000 |
| CB25 | Male | 6 | 0.011 | -0.037 | 0.060 | 0.027 | -0.010 | 0.064 | 13 | 12272.925 | 0.000 |
| CB25 | Male | 7 | 0.030 | -0.027 | 0.086 | 0.027 | -0.010 | 0.064 | 13 | 12272.925 | 0.000 |
| CB25 | Male | 8 | 0.034 | -0.013 | 0.081 | 0.027 | -0.010 | 0.064 | 13 | 12272.925 | 0.000 |
| CB25 | Male | 9 | 0.018 | -0.019 | 0.055 | 0.027 | -0.010 | 0.064 | 13 | 12272.925 | 0.000 |
| CB25 | Male | 10 | 0.040 | 0.008 | 0.072 | 0.027 | -0.010 | 0.064 | 13 | 12272.925 | 0.000 |
| CB25 | Female | -5 | 0.000 | -0.028 | 0.029 | 0.001 | -0.014 | 0.017 | 13 | 5301.372 | 0.000 |
| CB25 | Female | -4 | -0.017 | -0.039 | 0.006 | 0.001 | -0.014 | 0.017 | 13 | 5301.372 | 0.000 |
| CB25 | Female | -3 | 0.003 | -0.022 | 0.027 | 0.001 | -0.014 | 0.017 | 13 | 5301.372 | 0.000 |
| CB25 | Female | -2 | 0.019 | -0.024 | 0.062 | 0.001 | -0.014 | 0.017 | 13 | 5301.372 | 0.000 |
| CB25 | Female | -1 | 0.000 | 0.000 | 0.000 |  |  |  | 13 | 5301.372 | 0.000 |
| CB25 | Female | 0 | -0.009 | -0.029 | 0.010 | 0.002 | -0.014 | 0.018 | 13 | 5301.372 | 0.000 |
| CB25 | Female | 1 | -0.006 | -0.029 | 0.017 | 0.002 | -0.014 | 0.018 | 13 | 5301.372 | 0.000 |
| CB25 | Female | 2 | -0.010 | -0.041 | 0.021 | 0.002 | -0.014 | 0.018 | 13 | 5301.372 | 0.000 |
| CB25 | Female | 3 | -0.005 | -0.038 | 0.029 | 0.002 | -0.014 | 0.018 | 13 | 5301.372 | 0.000 |
| CB25 | Female | 4 | 0.028 | -0.005 | 0.062 | 0.002 | -0.014 | 0.018 | 13 | 5301.372 | 0.000 |
| CB25 | Female | 5 | 0.013 | -0.015 | 0.041 | 0.002 | -0.014 | 0.018 | 13 | 5301.372 | 0.000 |
| CB25 | Female | 6 | -0.008 | -0.040 | 0.023 | 0.002 | -0.014 | 0.018 | 13 | 5301.372 | 0.000 |
| CB25 | Female | 7 | 0.015 | -0.006 | 0.036 | 0.002 | -0.014 | 0.018 | 13 | 5301.372 | 0.000 |
| CB25 | Female | 8 | 0.023 | 0.006 | 0.041 | 0.002 | -0.014 | 0.018 | 13 | 5301.372 | 0.000 |
| CB25 | Female | 9 | -0.018 | -0.042 | 0.005 | 0.002 | -0.014 | 0.018 | 13 | 5301.372 | 0.000 |
| CB25 | Female | 10 | -0.003 | -0.027 | 0.020 | 0.002 | -0.014 | 0.018 | 13 | 5301.372 | 0.000 |
| CB30 | Male | -5 | -0.008 | -0.054 | 0.037 | 0.013 | -0.005 | 0.031 | 13 | 11545.474 | 0.000 |
| CB30 | Male | -4 | 0.035 | 0.007 | 0.062 | 0.013 | -0.005 | 0.031 | 13 | 11545.474 | 0.000 |
| CB30 | Male | -3 | 0.011 | -0.008 | 0.029 | 0.013 | -0.005 | 0.031 | 13 | 11545.474 | 0.000 |
| CB30 | Male | -2 | 0.014 | -0.002 | 0.030 | 0.013 | -0.005 | 0.031 | 13 | 11545.474 | 0.000 |
| CB30 | Male | -1 | 0.000 | 0.000 | 0.000 |  |  |  | 13 | 11545.474 | 0.000 |
| CB30 | Male | 0 | 0.009 | -0.020 | 0.038 | 0.018 | 0.000 | 0.036 | 13 | 11545.474 | 0.000 |
| CB30 | Male | 1 | 0.045 | 0.020 | 0.070 | 0.018 | 0.000 | 0.036 | 13 | 11545.474 | 0.000 |
| CB30 | Male | 2 | 0.005 | -0.024 | 0.033 | 0.018 | 0.000 | 0.036 | 13 | 11545.474 | 0.000 |
| CB30 | Male | 3 | 0.002 | -0.028 | 0.032 | 0.018 | 0.000 | 0.036 | 13 | 11545.474 | 0.000 |
| CB30 | Male | 4 | 0.026 | 0.003 | 0.050 | 0.018 | 0.000 | 0.036 | 13 | 11545.474 | 0.000 |
| CB30 | Male | 5 | 0.019 | -0.002 | 0.040 | 0.018 | 0.000 | 0.036 | 13 | 11545.474 | 0.000 |
| CB30 | Male | 6 | 0.011 | -0.012 | 0.034 | 0.018 | 0.000 | 0.036 | 13 | 11545.474 | 0.000 |
| CB30 | Male | 7 | 0.028 | -0.005 | 0.062 | 0.018 | 0.000 | 0.036 | 13 | 11545.474 | 0.000 |
| CB30 | Male | 8 | 0.032 | 0.009 | 0.055 | 0.018 | 0.000 | 0.036 | 13 | 11545.474 | 0.000 |
| CB30 | Male | 9 | 0.010 | -0.016 | 0.036 | 0.018 | 0.000 | 0.036 | 13 | 11545.474 | 0.000 |
| CB30 | Male | 10 | 0.013 | -0.006 | 0.032 | 0.018 | 0.000 | 0.036 | 13 | 11545.474 | 0.000 |
| CB30 | Female | -5 | -0.006 | -0.034 | 0.021 | -0.010 | -0.027 | 0.008 | 13 | 613.678 | 0.000 |
| CB30 | Female | -4 | -0.016 | -0.043 | 0.011 | -0.010 | -0.027 | 0.008 | 13 | 613.678 | 0.000 |
| CB30 | Female | -3 | -0.007 | -0.030 | 0.015 | -0.010 | -0.027 | 0.008 | 13 | 613.678 | 0.000 |
| CB30 | Female | -2 | -0.009 | -0.031 | 0.014 | -0.010 | -0.027 | 0.008 | 13 | 613.678 | 0.000 |
| CB30 | Female | -1 | 0.000 | 0.000 | 0.000 |  |  |  | 13 | 613.678 | 0.000 |
| CB30 | Female | 0 | -0.024 | -0.047 | -0.001 | -0.016 | -0.031 | -0.002 | 13 | 613.678 | 0.000 |
| CB30 | Female | 1 | -0.018 | -0.038 | 0.001 | -0.016 | -0.031 | -0.002 | 13 | 613.678 | 0.000 |
| CB30 | Female | 2 | -0.014 | -0.030 | 0.003 | -0.016 | -0.031 | -0.002 | 13 | 613.678 | 0.000 |
| CB30 | Female | 3 | -0.014 | -0.030 | 0.003 | -0.016 | -0.031 | -0.002 | 13 | 613.678 | 0.000 |
| CB30 | Female | 4 | 0.002 | -0.021 | 0.024 | -0.016 | -0.031 | -0.002 | 13 | 613.678 | 0.000 |
| CB30 | Female | 5 | -0.015 | -0.047 | 0.017 | -0.016 | -0.031 | -0.002 | 13 | 613.678 | 0.000 |
| CB30 | Female | 6 | -0.024 | -0.043 | -0.004 | -0.016 | -0.031 | -0.002 | 13 | 613.678 | 0.000 |
| CB30 | Female | 7 | -0.026 | -0.051 | -0.001 | -0.016 | -0.031 | -0.002 | 13 | 613.678 | 0.000 |
| CB30 | Female | 8 | -0.006 | -0.025 | 0.013 | -0.016 | -0.031 | -0.002 | 13 | 613.678 | 0.000 |
| CB30 | Female | 9 | -0.023 | -0.037 | -0.009 | -0.016 | -0.031 | -0.002 | 13 | 613.678 | 0.000 |
| CB30 | Female | 10 | -0.016 | -0.040 | 0.008 | -0.016 | -0.031 | -0.002 | 13 | 613.678 | 0.000 |
| CB35 | Male | -5 | 0.011 | -0.027 | 0.050 | 0.021 | 0.001 | 0.042 | 13 | 14699.600 | 0.000 |
| CB35 | Male | -4 | 0.037 | 0.015 | 0.058 | 0.021 | 0.001 | 0.042 | 13 | 14699.600 | 0.000 |
| CB35 | Male | -3 | 0.013 | -0.012 | 0.039 | 0.021 | 0.001 | 0.042 | 13 | 14699.600 | 0.000 |
| CB35 | Male | -2 | 0.023 | 0.011 | 0.036 | 0.021 | 0.001 | 0.042 | 13 | 14699.600 | 0.000 |
| CB35 | Male | -1 | 0.000 | 0.000 | 0.000 |  |  |  | 13 | 14699.600 | 0.000 |
| CB35 | Male | 0 | 0.023 | 0.002 | 0.044 | 0.025 | 0.009 | 0.041 | 13 | 14699.600 | 0.000 |
| CB35 | Male | 1 | 0.043 | 0.027 | 0.058 | 0.025 | 0.009 | 0.041 | 13 | 14699.600 | 0.000 |
| CB35 | Male | 2 | 0.022 | -0.010 | 0.055 | 0.025 | 0.009 | 0.041 | 13 | 14699.600 | 0.000 |
| CB35 | Male | 3 | 0.015 | -0.014 | 0.043 | 0.025 | 0.009 | 0.041 | 13 | 14699.600 | 0.000 |
| CB35 | Male | 4 | 0.030 | 0.015 | 0.045 | 0.025 | 0.009 | 0.041 | 13 | 14699.600 | 0.000 |
| CB35 | Male | 5 | 0.018 | 0.001 | 0.036 | 0.025 | 0.009 | 0.041 | 13 | 14699.600 | 0.000 |
| CB35 | Male | 6 | 0.001 | -0.015 | 0.017 | 0.025 | 0.009 | 0.041 | 13 | 14699.600 | 0.000 |
| CB35 | Male | 7 | 0.039 | 0.014 | 0.064 | 0.025 | 0.009 | 0.041 | 13 | 14699.600 | 0.000 |
| CB35 | Male | 8 | 0.038 | 0.020 | 0.056 | 0.025 | 0.009 | 0.041 | 13 | 14699.600 | 0.000 |
| CB35 | Male | 9 | 0.026 | -0.002 | 0.055 | 0.025 | 0.009 | 0.041 | 13 | 14699.600 | 0.000 |
| CB35 | Male | 10 | 0.019 | -0.003 | 0.042 | 0.025 | 0.009 | 0.041 | 13 | 14699.600 | 0.000 |
| CB35 | Female | -5 | -0.004 | -0.017 | 0.009 | -0.003 | -0.012 | 0.006 | 13 | 549.950 | 0.000 |
| CB35 | Female | -4 | 0.000 | -0.007 | 0.008 | -0.003 | -0.012 | 0.006 | 13 | 549.950 | 0.000 |
| CB35 | Female | -3 | -0.010 | -0.023 | 0.004 | -0.003 | -0.012 | 0.006 | 13 | 549.950 | 0.000 |
| CB35 | Female | -2 | 0.001 | -0.024 | 0.025 | -0.003 | -0.012 | 0.006 | 13 | 549.950 | 0.000 |
| CB35 | Female | -1 | 0.000 | 0.000 | 0.000 |  |  |  | 13 | 549.950 | 0.000 |
| CB35 | Female | 0 | -0.008 | -0.026 | 0.009 | -0.013 | -0.021 | -0.006 | 13 | 549.950 | 0.000 |
| CB35 | Female | 1 | -0.018 | -0.033 | -0.002 | -0.013 | -0.021 | -0.006 | 13 | 549.950 | 0.000 |
| CB35 | Female | 2 | -0.013 | -0.029 | 0.002 | -0.013 | -0.021 | -0.006 | 13 | 549.950 | 0.000 |
| CB35 | Female | 3 | -0.013 | -0.026 | 0.001 | -0.013 | -0.021 | -0.006 | 13 | 549.950 | 0.000 |
| CB35 | Female | 4 | -0.002 | -0.017 | 0.012 | -0.013 | -0.021 | -0.006 | 13 | 549.950 | 0.000 |
| CB35 | Female | 5 | -0.009 | -0.029 | 0.012 | -0.013 | -0.021 | -0.006 | 13 | 549.950 | 0.000 |
| CB35 | Female | 6 | -0.019 | -0.039 | 0.000 | -0.013 | -0.021 | -0.006 | 13 | 549.950 | 0.000 |
| CB35 | Female | 7 | -0.019 | -0.032 | -0.007 | -0.013 | -0.021 | -0.006 | 13 | 549.950 | 0.000 |
| CB35 | Female | 8 | -0.008 | -0.019 | 0.002 | -0.013 | -0.021 | -0.006 | 13 | 549.950 | 0.000 |
| CB35 | Female | 9 | -0.021 | -0.033 | -0.009 | -0.013 | -0.021 | -0.006 | 13 | 549.950 | 0.000 |
| CB35 | Female | 10 | -0.015 | -0.030 | 0.000 | -0.013 | -0.021 | -0.006 | 13 | 549.950 | 0.000 |
| CB40 | Male | -5 | 0.011 | -0.017 | 0.040 | 0.016 | 0.000 | 0.032 | 13 | 2475.891 | 0.000 |
| CB40 | Male | -4 | 0.025 | 0.001 | 0.050 | 0.016 | 0.000 | 0.032 | 13 | 2475.891 | 0.000 |
| CB40 | Male | -3 | 0.010 | -0.011 | 0.032 | 0.016 | 0.000 | 0.032 | 13 | 2475.891 | 0.000 |
| CB40 | Male | -2 | 0.018 | 0.006 | 0.029 | 0.016 | 0.000 | 0.032 | 13 | 2475.891 | 0.000 |
| CB40 | Male | -1 | 0.000 | 0.000 | 0.000 |  |  |  | 13 | 2475.891 | 0.000 |
| CB40 | Male | 0 | 0.017 | -0.001 | 0.036 | 0.015 | 0.003 | 0.027 | 13 | 2475.891 | 0.000 |
| CB40 | Male | 1 | 0.031 | 0.016 | 0.046 | 0.015 | 0.003 | 0.027 | 13 | 2475.891 | 0.000 |
| CB40 | Male | 2 | 0.004 | -0.021 | 0.029 | 0.015 | 0.003 | 0.027 | 13 | 2475.891 | 0.000 |
| CB40 | Male | 3 | 0.008 | -0.008 | 0.025 | 0.015 | 0.003 | 0.027 | 13 | 2475.891 | 0.000 |
| CB40 | Male | 4 | 0.023 | 0.010 | 0.036 | 0.015 | 0.003 | 0.027 | 13 | 2475.891 | 0.000 |
| CB40 | Male | 5 | 0.005 | -0.015 | 0.024 | 0.015 | 0.003 | 0.027 | 13 | 2475.891 | 0.000 |
| CB40 | Male | 6 | 0.000 | -0.015 | 0.016 | 0.015 | 0.003 | 0.027 | 13 | 2475.891 | 0.000 |
| CB40 | Male | 7 | 0.025 | 0.006 | 0.044 | 0.015 | 0.003 | 0.027 | 13 | 2475.891 | 0.000 |
| CB40 | Male | 8 | 0.027 | 0.006 | 0.048 | 0.015 | 0.003 | 0.027 | 13 | 2475.891 | 0.000 |
| CB40 | Male | 9 | 0.012 | -0.012 | 0.037 | 0.015 | 0.003 | 0.027 | 13 | 2475.891 | 0.000 |
| CB40 | Male | 10 | 0.017 | 0.001 | 0.032 | 0.015 | 0.003 | 0.027 | 13 | 2475.891 | 0.000 |
| CB40 | Female | -5 | 0.003 | -0.011 | 0.017 | 0.000 | -0.009 | 0.009 | 13 | 6725.860 | 0.000 |
| CB40 | Female | -4 | 0.002 | -0.014 | 0.018 | 0.000 | -0.009 | 0.009 | 13 | 6725.860 | 0.000 |
| CB40 | Female | -3 | -0.006 | -0.021 | 0.009 | 0.000 | -0.009 | 0.009 | 13 | 6725.860 | 0.000 |
| CB40 | Female | -2 | 0.000 | -0.017 | 0.016 | 0.000 | -0.009 | 0.009 | 13 | 6725.860 | 0.000 |
| CB40 | Female | -1 | 0.000 | 0.000 | 0.000 |  |  |  | 13 | 6725.860 | 0.000 |
| CB40 | Female | 0 | -0.009 | -0.025 | 0.007 | -0.015 | -0.020 | -0.009 | 13 | 6725.860 | 0.000 |
| CB40 | Female | 1 | -0.013 | -0.028 | 0.003 | -0.015 | -0.020 | -0.009 | 13 | 6725.860 | 0.000 |
| CB40 | Female | 2 | -0.017 | -0.028 | -0.006 | -0.015 | -0.020 | -0.009 | 13 | 6725.860 | 0.000 |
| CB40 | Female | 3 | -0.016 | -0.030 | -0.002 | -0.015 | -0.020 | -0.009 | 13 | 6725.860 | 0.000 |
| CB40 | Female | 4 | -0.009 | -0.018 | 0.000 | -0.015 | -0.020 | -0.009 | 13 | 6725.860 | 0.000 |
| CB40 | Female | 5 | -0.006 | -0.024 | 0.011 | -0.015 | -0.020 | -0.009 | 13 | 6725.860 | 0.000 |
| CB40 | Female | 6 | -0.025 | -0.039 | -0.011 | -0.015 | -0.020 | -0.009 | 13 | 6725.860 | 0.000 |
| CB40 | Female | 7 | -0.019 | -0.029 | -0.008 | -0.015 | -0.020 | -0.009 | 13 | 6725.860 | 0.000 |
| CB40 | Female | 8 | -0.015 | -0.026 | -0.004 | -0.015 | -0.020 | -0.009 | 13 | 6725.860 | 0.000 |
| CB40 | Female | 9 | -0.019 | -0.034 | -0.004 | -0.015 | -0.020 | -0.009 | 13 | 6725.860 | 0.000 |
| CB40 | Female | 10 | -0.012 | -0.024 | 0.000 | -0.015 | -0.020 | -0.009 | 13 | 6725.860 | 0.000 |
| MB20 | Male | -5 | 0.003 | -0.004 | 0.009 | -0.001 | -0.003 | 0.001 | 5 | 28.618 | .00002755 |
| MB20 | Male | -4 | -0.002 | -0.003 | 0.000 | -0.001 | -0.003 | 0.001 | 5 | 28.618 | .00002755 |
| MB20 | Male | -3 | -0.004 | -0.006 | -0.002 | -0.001 | -0.003 | 0.001 | 5 | 28.618 | .00002755 |
| MB20 | Male | -2 | 0.000 | -0.003 | 0.003 | -0.001 | -0.003 | 0.001 | 5 | 28.618 | .00002755 |
| MB20 | Male | -1 | 0.000 | 0.000 | 0.000 |  |  |  | 5 | 28.618 | .00002755 |
| MB20 | Male | 0 | 0.000 | -0.001 | 0.001 | -0.003 | -0.006 | 0.000 | 5 | 28.618 | .00002755 |
| MB20 | Male | 1 | -0.003 | -0.006 | -0.001 | -0.003 | -0.006 | 0.000 | 5 | 28.618 | .00002755 |
| MB20 | Male | 2 | -0.001 | -0.004 | 0.002 | -0.003 | -0.006 | 0.000 | 5 | 28.618 | .00002755 |
| MB20 | Male | 3 | -0.003 | -0.006 | 0.000 | -0.003 | -0.006 | 0.000 | 5 | 28.618 | .00002755 |
| MB20 | Male | 4 | -0.003 | -0.006 | 0.000 | -0.003 | -0.006 | 0.000 | 5 | 28.618 | .00002755 |
| MB20 | Male | 5 | -0.003 | -0.008 | 0.002 | -0.003 | -0.006 | 0.000 | 5 | 28.618 | .00002755 |
| MB20 | Male | 6 | -0.002 | -0.004 | 0.000 | -0.003 | -0.006 | 0.000 | 5 | 28.618 | .00002755 |
| MB20 | Male | 7 | -0.004 | -0.008 | 0.000 | -0.003 | -0.006 | 0.000 | 5 | 28.618 | .00002755 |
| MB20 | Male | 8 | -0.003 | -0.007 | 0.001 | -0.003 | -0.006 | 0.000 | 5 | 28.618 | .00002755 |
| MB20 | Male | 9 | -0.003 | -0.007 | 0.000 | -0.003 | -0.006 | 0.000 | 5 | 28.618 | .00002755 |
| MB20 | Male | 10 | -0.003 | -0.007 | 0.000 | -0.003 | -0.006 | 0.000 | 5 | 28.618 | .00002755 |
| MB20 | Female | -5 | -0.005 | -0.024 | 0.013 | -0.004 | -0.015 | 0.007 | 5 | 14.335 | .01361344 |
| MB20 | Female | -4 | -0.011 | -0.025 | 0.003 | -0.004 | -0.015 | 0.007 | 5 | 14.335 | .01361344 |
| MB20 | Female | -3 | -0.003 | -0.011 | 0.005 | -0.004 | -0.015 | 0.007 | 5 | 14.335 | .01361344 |
| MB20 | Female | -2 | 0.002 | -0.009 | 0.014 | -0.004 | -0.015 | 0.007 | 5 | 14.335 | .01361344 |
| MB20 | Female | -1 | 0.000 | 0.000 | 0.000 |  |  |  | 5 | 14.335 | .01361344 |
| MB20 | Female | 0 | 0.009 | 0.002 | 0.016 | -0.005 | -0.017 | 0.006 | 5 | 14.335 | .01361344 |
| MB20 | Female | 1 | -0.002 | -0.015 | 0.011 | -0.005 | -0.017 | 0.006 | 5 | 14.335 | .01361344 |
| MB20 | Female | 2 | -0.003 | -0.013 | 0.008 | -0.005 | -0.017 | 0.006 | 5 | 14.335 | .01361344 |
| MB20 | Female | 3 | 0.002 | -0.006 | 0.011 | -0.005 | -0.017 | 0.006 | 5 | 14.335 | .01361344 |
| MB20 | Female | 4 | 0.004 | -0.008 | 0.016 | -0.005 | -0.017 | 0.006 | 5 | 14.335 | .01361344 |
| MB20 | Female | 5 | -0.011 | -0.026 | 0.005 | -0.005 | -0.017 | 0.006 | 5 | 14.335 | .01361344 |
| MB20 | Female | 6 | -0.010 | -0.027 | 0.008 | -0.005 | -0.017 | 0.006 | 5 | 14.335 | .01361344 |
| MB20 | Female | 7 | -0.013 | -0.032 | 0.005 | -0.005 | -0.017 | 0.006 | 5 | 14.335 | .01361344 |
| MB20 | Female | 8 | -0.012 | -0.035 | 0.012 | -0.005 | -0.017 | 0.006 | 5 | 14.335 | .01361344 |
| MB20 | Female | 9 | -0.012 | -0.031 | 0.006 | -0.005 | -0.017 | 0.006 | 5 | 14.335 | .01361344 |
| MB20 | Female | 10 | -0.011 | -0.032 | 0.011 | -0.005 | -0.017 | 0.006 | 5 | 14.335 | .01361344 |
| MB25 | Male | -5 | 0.015 | -0.025 | 0.055 | 0.028 | 0.004 | 0.053 | 12 | 2614.994 | 0.000 |
| MB25 | Male | -4 | 0.037 | 0.019 | 0.055 | 0.028 | 0.004 | 0.053 | 12 | 2614.994 | 0.000 |
| MB25 | Male | -3 | 0.028 | 0.006 | 0.051 | 0.028 | 0.004 | 0.053 | 12 | 2614.994 | 0.000 |
| MB25 | Male | -2 | 0.033 | 0.009 | 0.058 | 0.028 | 0.004 | 0.053 | 12 | 2614.994 | 0.000 |
| MB25 | Male | -1 | 0.000 | 0.000 | 0.000 |  |  |  | 12 | 2614.994 | 0.000 |
| MB25 | Male | 0 | 0.018 | -0.016 | 0.052 | 0.015 | -0.025 | 0.056 | 12 | 2614.994 | 0.000 |
| MB25 | Male | 1 | 0.031 | 0.008 | 0.054 | 0.015 | -0.025 | 0.056 | 12 | 2614.994 | 0.000 |
| MB25 | Male | 2 | 0.031 | -0.009 | 0.072 | 0.015 | -0.025 | 0.056 | 12 | 2614.994 | 0.000 |
| MB25 | Male | 3 | 0.018 | -0.015 | 0.052 | 0.015 | -0.025 | 0.056 | 12 | 2614.994 | 0.000 |
| MB25 | Male | 4 | 0.020 | -0.020 | 0.060 | 0.015 | -0.025 | 0.056 | 12 | 2614.994 | 0.000 |
| MB25 | Male | 5 | 0.011 | -0.026 | 0.048 | 0.015 | -0.025 | 0.056 | 12 | 2614.994 | 0.000 |
| MB25 | Male | 6 | 0.007 | -0.047 | 0.061 | 0.015 | -0.025 | 0.056 | 12 | 2614.994 | 0.000 |
| MB25 | Male | 7 | 0.020 | -0.038 | 0.078 | 0.015 | -0.025 | 0.056 | 12 | 2614.994 | 0.000 |
| MB25 | Male | 8 | 0.009 | -0.043 | 0.062 | 0.015 | -0.025 | 0.056 | 12 | 2614.994 | 0.000 |
| MB25 | Male | 9 | -0.002 | -0.058 | 0.054 | 0.015 | -0.025 | 0.056 | 12 | 2614.994 | 0.000 |
| MB25 | Male | 10 | 0.005 | -0.047 | 0.056 | 0.015 | -0.025 | 0.056 | 12 | 2614.994 | 0.000 |
| MB25 | Female | -5 | -0.036 | -0.089 | 0.016 | -0.020 | -0.051 | 0.010 | 12 | 1951.347 | 0.000 |
| MB25 | Female | -4 | -0.017 | -0.051 | 0.016 | -0.020 | -0.051 | 0.010 | 12 | 1951.347 | 0.000 |
| MB25 | Female | -3 | -0.019 | -0.051 | 0.014 | -0.020 | -0.051 | 0.010 | 12 | 1951.347 | 0.000 |
| MB25 | Female | -2 | -0.008 | -0.037 | 0.021 | -0.020 | -0.051 | 0.010 | 12 | 1951.347 | 0.000 |
| MB25 | Female | -1 | 0.000 | 0.000 | 0.000 |  |  |  | 12 | 1951.347 | 0.000 |
| MB25 | Female | 0 | -0.012 | -0.051 | 0.027 | -0.019 | -0.054 | 0.015 | 12 | 1951.347 | 0.000 |
| MB25 | Female | 1 | -0.033 | -0.082 | 0.016 | -0.019 | -0.054 | 0.015 | 12 | 1951.347 | 0.000 |
| MB25 | Female | 2 | -0.017 | -0.070 | 0.036 | -0.019 | -0.054 | 0.015 | 12 | 1951.347 | 0.000 |
| MB25 | Female | 3 | -0.017 | -0.042 | 0.008 | -0.019 | -0.054 | 0.015 | 12 | 1951.347 | 0.000 |
| MB25 | Female | 4 | -0.004 | -0.038 | 0.031 | -0.019 | -0.054 | 0.015 | 12 | 1951.347 | 0.000 |
| MB25 | Female | 5 | -0.015 | -0.058 | 0.027 | -0.019 | -0.054 | 0.015 | 12 | 1951.347 | 0.000 |
| MB25 | Female | 6 | -0.032 | -0.070 | 0.006 | -0.019 | -0.054 | 0.015 | 12 | 1951.347 | 0.000 |
| MB25 | Female | 7 | -0.009 | -0.050 | 0.032 | -0.019 | -0.054 | 0.015 | 12 | 1951.347 | 0.000 |
| MB25 | Female | 8 | 0.002 | -0.030 | 0.034 | -0.019 | -0.054 | 0.015 | 12 | 1951.347 | 0.000 |
| MB25 | Female | 9 | -0.035 | -0.072 | 0.002 | -0.019 | -0.054 | 0.015 | 12 | 1951.347 | 0.000 |
| MB25 | Female | 10 | -0.041 | -0.074 | -0.008 | -0.019 | -0.054 | 0.015 | 12 | 1951.347 | 0.000 |
| MB30 | Male | -5 | -0.006 | -0.062 | 0.050 | 0.010 | -0.026 | 0.045 | 13 | 13442.014 | 0.000 |
| MB30 | Male | -4 | 0.009 | -0.035 | 0.054 | 0.010 | -0.026 | 0.045 | 13 | 13442.014 | 0.000 |
| MB30 | Male | -3 | 0.007 | -0.030 | 0.045 | 0.010 | -0.026 | 0.045 | 13 | 13442.014 | 0.000 |
| MB30 | Male | -2 | 0.028 | 0.005 | 0.051 | 0.010 | -0.026 | 0.045 | 13 | 13442.014 | 0.000 |
| MB30 | Male | -1 | 0.000 | 0.000 | 0.000 |  |  |  | 13 | 13442.014 | 0.000 |
| MB30 | Male | 0 | 0.020 | -0.007 | 0.047 | 0.018 | 0.001 | 0.035 | 13 | 13442.014 | 0.000 |
| MB30 | Male | 1 | 0.032 | 0.015 | 0.050 | 0.018 | 0.001 | 0.035 | 13 | 13442.014 | 0.000 |
| MB30 | Male | 2 | 0.007 | -0.014 | 0.029 | 0.018 | 0.001 | 0.035 | 13 | 13442.014 | 0.000 |
| MB30 | Male | 3 | -0.004 | -0.031 | 0.023 | 0.018 | 0.001 | 0.035 | 13 | 13442.014 | 0.000 |
| MB30 | Male | 4 | 0.025 | 0.007 | 0.043 | 0.018 | 0.001 | 0.035 | 13 | 13442.014 | 0.000 |
| MB30 | Male | 5 | 0.026 | -0.001 | 0.052 | 0.018 | 0.001 | 0.035 | 13 | 13442.014 | 0.000 |
| MB30 | Male | 6 | 0.017 | -0.009 | 0.044 | 0.018 | 0.001 | 0.035 | 13 | 13442.014 | 0.000 |
| MB30 | Male | 7 | 0.019 | -0.012 | 0.050 | 0.018 | 0.001 | 0.035 | 13 | 13442.014 | 0.000 |
| MB30 | Male | 8 | 0.023 | -0.007 | 0.053 | 0.018 | 0.001 | 0.035 | 13 | 13442.014 | 0.000 |
| MB30 | Male | 9 | 0.014 | -0.012 | 0.039 | 0.018 | 0.001 | 0.035 | 13 | 13442.014 | 0.000 |
| MB30 | Male | 10 | 0.023 | -0.001 | 0.046 | 0.018 | 0.001 | 0.035 | 13 | 13442.014 | 0.000 |
| MB30 | Female | -5 | -0.050 | -0.086 | -0.015 | -0.037 | -0.059 | -0.015 | 13 | 12630.458 | 0.000 |
| MB30 | Female | -4 | -0.033 | -0.058 | -0.009 | -0.037 | -0.059 | -0.015 | 13 | 12630.458 | 0.000 |
| MB30 | Female | -3 | -0.041 | -0.073 | -0.010 | -0.037 | -0.059 | -0.015 | 13 | 12630.458 | 0.000 |
| MB30 | Female | -2 | -0.022 | -0.045 | 0.001 | -0.037 | -0.059 | -0.015 | 13 | 12630.458 | 0.000 |
| MB30 | Female | -1 | 0.000 | 0.000 | 0.000 |  |  |  | 13 | 12630.458 | 0.000 |
| MB30 | Female | 0 | -0.026 | -0.064 | 0.011 | -0.027 | -0.070 | 0.016 | 13 | 12630.458 | 0.000 |
| MB30 | Female | 1 | -0.041 | -0.074 | -0.009 | -0.027 | -0.070 | 0.016 | 13 | 12630.458 | 0.000 |
| MB30 | Female | 2 | -0.029 | -0.078 | 0.019 | -0.027 | -0.070 | 0.016 | 13 | 12630.458 | 0.000 |
| MB30 | Female | 3 | -0.030 | -0.060 | 0.000 | -0.027 | -0.070 | 0.016 | 13 | 12630.458 | 0.000 |
| MB30 | Female | 4 | -0.009 | -0.042 | 0.024 | -0.027 | -0.070 | 0.016 | 13 | 12630.458 | 0.000 |
| MB30 | Female | 5 | -0.036 | -0.090 | 0.017 | -0.027 | -0.070 | 0.016 | 13 | 12630.458 | 0.000 |
| MB30 | Female | 6 | -0.053 | -0.099 | -0.006 | -0.027 | -0.070 | 0.016 | 13 | 12630.458 | 0.000 |
| MB30 | Female | 7 | -0.020 | -0.064 | 0.025 | -0.027 | -0.070 | 0.016 | 13 | 12630.458 | 0.000 |
| MB30 | Female | 8 | 0.005 | -0.042 | 0.052 | -0.027 | -0.070 | 0.016 | 13 | 12630.458 | 0.000 |
| MB30 | Female | 9 | -0.033 | -0.099 | 0.034 | -0.027 | -0.070 | 0.016 | 13 | 12630.458 | 0.000 |
| MB30 | Female | 10 | -0.025 | -0.087 | 0.038 | -0.027 | -0.070 | 0.016 | 13 | 12630.458 | 0.000 |
| MB35 | Male | -5 | 0.000 | -0.049 | 0.049 | 0.011 | -0.015 | 0.038 | 13 | 3115.132 | 0.000 |
| MB35 | Male | -4 | 0.018 | -0.023 | 0.060 | 0.011 | -0.015 | 0.038 | 13 | 3115.132 | 0.000 |
| MB35 | Male | -3 | 0.002 | -0.023 | 0.027 | 0.011 | -0.015 | 0.038 | 13 | 3115.132 | 0.000 |
| MB35 | Male | -2 | 0.026 | 0.012 | 0.041 | 0.011 | -0.015 | 0.038 | 13 | 3115.132 | 0.000 |
| MB35 | Male | -1 | 0.000 | 0.000 | 0.000 |  |  |  | 13 | 3115.132 | 0.000 |
| MB35 | Male | 0 | 0.011 | -0.020 | 0.042 | 0.019 | -0.001 | 0.039 | 13 | 3115.132 | 0.000 |
| MB35 | Male | 1 | 0.038 | 0.018 | 0.057 | 0.019 | -0.001 | 0.039 | 13 | 3115.132 | 0.000 |
| MB35 | Male | 2 | 0.011 | -0.014 | 0.036 | 0.019 | -0.001 | 0.039 | 13 | 3115.132 | 0.000 |
| MB35 | Male | 3 | 0.003 | -0.019 | 0.025 | 0.019 | -0.001 | 0.039 | 13 | 3115.132 | 0.000 |
| MB35 | Male | 4 | 0.027 | 0.001 | 0.053 | 0.019 | -0.001 | 0.039 | 13 | 3115.132 | 0.000 |
| MB35 | Male | 5 | 0.037 | 0.012 | 0.062 | 0.019 | -0.001 | 0.039 | 13 | 3115.132 | 0.000 |
| MB35 | Male | 6 | -0.005 | -0.031 | 0.020 | 0.019 | -0.001 | 0.039 | 13 | 3115.132 | 0.000 |
| MB35 | Male | 7 | 0.019 | -0.009 | 0.047 | 0.019 | -0.001 | 0.039 | 13 | 3115.132 | 0.000 |
| MB35 | Male | 8 | 0.029 | 0.009 | 0.049 | 0.019 | -0.001 | 0.039 | 13 | 3115.132 | 0.000 |
| MB35 | Male | 9 | 0.017 | -0.014 | 0.049 | 0.019 | -0.001 | 0.039 | 13 | 3115.132 | 0.000 |
| MB35 | Male | 10 | 0.024 | -0.015 | 0.062 | 0.019 | -0.001 | 0.039 | 13 | 3115.132 | 0.000 |
| MB35 | Female | -5 | -0.043 | -0.076 | -0.011 | -0.029 | -0.047 | -0.010 | 13 | 1445.061 | 0.000 |
| MB35 | Female | -4 | -0.028 | -0.045 | -0.012 | -0.029 | -0.047 | -0.010 | 13 | 1445.061 | 0.000 |
| MB35 | Female | -3 | -0.032 | -0.051 | -0.013 | -0.029 | -0.047 | -0.010 | 13 | 1445.061 | 0.000 |
| MB35 | Female | -2 | -0.011 | -0.032 | 0.010 | -0.029 | -0.047 | -0.010 | 13 | 1445.061 | 0.000 |
| MB35 | Female | -1 | 0.000 | 0.000 | 0.000 |  |  |  | 13 | 1445.061 | 0.000 |
| MB35 | Female | 0 | -0.028 | -0.054 | -0.002 | -0.019 | -0.057 | 0.020 | 13 | 1445.061 | 0.000 |
| MB35 | Female | 1 | -0.036 | -0.067 | -0.005 | -0.019 | -0.057 | 0.020 | 13 | 1445.061 | 0.000 |
| MB35 | Female | 2 | -0.027 | -0.068 | 0.014 | -0.019 | -0.057 | 0.020 | 13 | 1445.061 | 0.000 |
| MB35 | Female | 3 | -0.009 | -0.045 | 0.027 | -0.019 | -0.057 | 0.020 | 13 | 1445.061 | 0.000 |
| MB35 | Female | 4 | 0.000 | -0.034 | 0.035 | -0.019 | -0.057 | 0.020 | 13 | 1445.061 | 0.000 |
| MB35 | Female | 5 | -0.025 | -0.075 | 0.025 | -0.019 | -0.057 | 0.020 | 13 | 1445.061 | 0.000 |
| MB35 | Female | 6 | -0.035 | -0.087 | 0.017 | -0.019 | -0.057 | 0.020 | 13 | 1445.061 | 0.000 |
| MB35 | Female | 7 | -0.020 | -0.061 | 0.021 | -0.019 | -0.057 | 0.020 | 13 | 1445.061 | 0.000 |
| MB35 | Female | 8 | 0.017 | -0.024 | 0.059 | -0.019 | -0.057 | 0.020 | 13 | 1445.061 | 0.000 |
| MB35 | Female | 9 | -0.021 | -0.077 | 0.035 | -0.019 | -0.057 | 0.020 | 13 | 1445.061 | 0.000 |
| MB35 | Female | 10 | -0.021 | -0.069 | 0.026 | -0.019 | -0.057 | 0.020 | 13 | 1445.061 | 0.000 |
| MB40 | Male | -5 | 0.015 | -0.037 | 0.068 | 0.018 | -0.007 | 0.043 | 13 | 2596.221 | 0.000 |
| MB40 | Male | -4 | 0.020 | -0.003 | 0.042 | 0.018 | -0.007 | 0.043 | 13 | 2596.221 | 0.000 |
| MB40 | Male | -3 | 0.011 | -0.015 | 0.037 | 0.018 | -0.007 | 0.043 | 13 | 2596.221 | 0.000 |
| MB40 | Male | -2 | 0.026 | 0.008 | 0.044 | 0.018 | -0.007 | 0.043 | 13 | 2596.221 | 0.000 |
| MB40 | Male | -1 | 0.000 | 0.000 | 0.000 |  |  |  | 13 | 2596.221 | 0.000 |
| MB40 | Male | 0 | 0.021 | -0.014 | 0.056 | 0.022 | -0.001 | 0.045 | 13 | 2596.221 | 0.000 |
| MB40 | Male | 1 | 0.035 | 0.015 | 0.056 | 0.022 | -0.001 | 0.045 | 13 | 2596.221 | 0.000 |
| MB40 | Male | 2 | 0.020 | -0.008 | 0.049 | 0.022 | -0.001 | 0.045 | 13 | 2596.221 | 0.000 |
| MB40 | Male | 3 | 0.011 | -0.015 | 0.036 | 0.022 | -0.001 | 0.045 | 13 | 2596.221 | 0.000 |
| MB40 | Male | 4 | 0.034 | 0.009 | 0.059 | 0.022 | -0.001 | 0.045 | 13 | 2596.221 | 0.000 |
| MB40 | Male | 5 | 0.031 | 0.005 | 0.058 | 0.022 | -0.001 | 0.045 | 13 | 2596.221 | 0.000 |
| MB40 | Male | 6 | -0.008 | -0.034 | 0.017 | 0.022 | -0.001 | 0.045 | 13 | 2596.221 | 0.000 |
| MB40 | Male | 7 | 0.025 | -0.009 | 0.059 | 0.022 | -0.001 | 0.045 | 13 | 2596.221 | 0.000 |
| MB40 | Male | 8 | 0.030 | 0.004 | 0.056 | 0.022 | -0.001 | 0.045 | 13 | 2596.221 | 0.000 |
| MB40 | Male | 9 | 0.019 | -0.011 | 0.048 | 0.022 | -0.001 | 0.045 | 13 | 2596.221 | 0.000 |
| MB40 | Male | 10 | 0.021 | -0.014 | 0.056 | 0.022 | -0.001 | 0.045 | 13 | 2596.221 | 0.000 |
| MB40 | Female | -5 | -0.030 | -0.051 | -0.009 | -0.018 | -0.031 | -0.004 | 13 | 1335.586 | 0.000 |
| MB40 | Female | -4 | -0.017 | -0.033 | -0.001 | -0.018 | -0.031 | -0.004 | 13 | 1335.586 | 0.000 |
| MB40 | Female | -3 | -0.024 | -0.043 | -0.006 | -0.018 | -0.031 | -0.004 | 13 | 1335.586 | 0.000 |
| MB40 | Female | -2 | 0.001 | -0.015 | 0.017 | -0.018 | -0.031 | -0.004 | 13 | 1335.586 | 0.000 |
| MB40 | Female | -1 | 0.000 | 0.000 | 0.000 |  |  |  | 13 | 1335.586 | 0.000 |
| MB40 | Female | 0 | -0.024 | -0.048 | 0.000 | -0.014 | -0.045 | 0.017 | 13 | 1335.586 | 0.000 |
| MB40 | Female | 1 | -0.023 | -0.049 | 0.003 | -0.014 | -0.045 | 0.017 | 13 | 1335.586 | 0.000 |
| MB40 | Female | 2 | -0.017 | -0.054 | 0.020 | -0.014 | -0.045 | 0.017 | 13 | 1335.586 | 0.000 |
| MB40 | Female | 3 | -0.009 | -0.040 | 0.022 | -0.014 | -0.045 | 0.017 | 13 | 1335.586 | 0.000 |
| MB40 | Female | 4 | 0.000 | -0.029 | 0.030 | -0.014 | -0.045 | 0.017 | 13 | 1335.586 | 0.000 |
| MB40 | Female | 5 | -0.026 | -0.070 | 0.018 | -0.014 | -0.045 | 0.017 | 13 | 1335.586 | 0.000 |
| MB40 | Female | 6 | -0.034 | -0.075 | 0.007 | -0.014 | -0.045 | 0.017 | 13 | 1335.586 | 0.000 |
| MB40 | Female | 7 | -0.004 | -0.040 | 0.032 | -0.014 | -0.045 | 0.017 | 13 | 1335.586 | 0.000 |
| MB40 | Female | 8 | 0.019 | -0.016 | 0.053 | -0.014 | -0.045 | 0.017 | 13 | 1335.586 | 0.000 |
| MB40 | Female | 9 | -0.024 | -0.069 | 0.021 | -0.014 | -0.045 | 0.017 | 13 | 1335.586 | 0.000 |
| MB40 | Female | 10 | -0.012 | -0.049 | 0.025 | -0.014 | -0.045 | 0.017 | 13 | 1335.586 | 0.000 |
| SR25 | Male | -5 | -0.008 | -0.041 | 0.025 | -0.004 | -0.031 | 0.023 | 13 | 3158.898 | 0.000 |
| SR25 | Male | -4 | 0.005 | -0.020 | 0.031 | -0.004 | -0.031 | 0.023 | 13 | 3158.898 | 0.000 |
| SR25 | Male | -3 | -0.015 | -0.054 | 0.025 | -0.004 | -0.031 | 0.023 | 13 | 3158.898 | 0.000 |
| SR25 | Male | -2 | 0.001 | -0.029 | 0.032 | -0.004 | -0.031 | 0.023 | 13 | 3158.898 | 0.000 |
| SR25 | Male | -1 | 0.000 | 0.000 | 0.000 |  |  |  | 13 | 3158.898 | 0.000 |
| SR25 | Male | 0 | -0.012 | -0.043 | 0.018 | -0.002 | -0.024 | 0.020 | 13 | 3158.898 | 0.000 |
| SR25 | Male | 1 | -0.004 | -0.029 | 0.022 | -0.002 | -0.024 | 0.020 | 13 | 3158.898 | 0.000 |
| SR25 | Male | 2 | 0.001 | -0.028 | 0.030 | -0.002 | -0.024 | 0.020 | 13 | 3158.898 | 0.000 |
| SR25 | Male | 3 | 0.002 | -0.022 | 0.027 | -0.002 | -0.024 | 0.020 | 13 | 3158.898 | 0.000 |
| SR25 | Male | 4 | 0.002 | -0.032 | 0.035 | -0.002 | -0.024 | 0.020 | 13 | 3158.898 | 0.000 |
| SR25 | Male | 5 | 0.005 | -0.013 | 0.023 | -0.002 | -0.024 | 0.020 | 13 | 3158.898 | 0.000 |
| SR25 | Male | 6 | 0.010 | -0.025 | 0.045 | -0.002 | -0.024 | 0.020 | 13 | 3158.898 | 0.000 |
| SR25 | Male | 7 | -0.003 | -0.033 | 0.027 | -0.002 | -0.024 | 0.020 | 13 | 3158.898 | 0.000 |
| SR25 | Male | 8 | -0.016 | -0.039 | 0.008 | -0.002 | -0.024 | 0.020 | 13 | 3158.898 | 0.000 |
| SR25 | Male | 9 | 0.008 | -0.021 | 0.038 | -0.002 | -0.024 | 0.020 | 13 | 3158.898 | 0.000 |
| SR25 | Male | 10 | -0.018 | -0.046 | 0.010 | -0.002 | -0.024 | 0.020 | 13 | 3158.898 | 0.000 |
| SR25 | Female | -5 | 0.037 | 0.018 | 0.056 | 0.011 | -0.008 | 0.030 | 13 | 1928.744 | 0.000 |
| SR25 | Female | -4 | -0.007 | -0.034 | 0.020 | 0.011 | -0.008 | 0.030 | 13 | 1928.744 | 0.000 |
| SR25 | Female | -3 | -0.014 | -0.055 | 0.027 | 0.011 | -0.008 | 0.030 | 13 | 1928.744 | 0.000 |
| SR25 | Female | -2 | 0.026 | 0.011 | 0.041 | 0.011 | -0.008 | 0.030 | 13 | 1928.744 | 0.000 |
| SR25 | Female | -1 | 0.000 | 0.000 | 0.000 |  |  |  | 13 | 1928.744 | 0.000 |
| SR25 | Female | 0 | 0.021 | 0.008 | 0.033 | 0.000 | -0.018 | 0.017 | 13 | 1928.744 | 0.000 |
| SR25 | Female | 1 | 0.003 | -0.025 | 0.031 | 0.000 | -0.018 | 0.017 | 13 | 1928.744 | 0.000 |
| SR25 | Female | 2 | 0.007 | -0.016 | 0.031 | 0.000 | -0.018 | 0.017 | 13 | 1928.744 | 0.000 |
| SR25 | Female | 3 | 0.006 | -0.033 | 0.046 | 0.000 | -0.018 | 0.017 | 13 | 1928.744 | 0.000 |
| SR25 | Female | 4 | -0.003 | -0.022 | 0.017 | 0.000 | -0.018 | 0.017 | 13 | 1928.744 | 0.000 |
| SR25 | Female | 5 | -0.016 | -0.044 | 0.013 | 0.000 | -0.018 | 0.017 | 13 | 1928.744 | 0.000 |
| SR25 | Female | 6 | 0.000 | -0.027 | 0.026 | 0.000 | -0.018 | 0.017 | 13 | 1928.744 | 0.000 |
| SR25 | Female | 7 | 0.009 | -0.023 | 0.040 | 0.000 | -0.018 | 0.017 | 13 | 1928.744 | 0.000 |
| SR25 | Female | 8 | -0.001 | -0.020 | 0.018 | 0.000 | -0.018 | 0.017 | 13 | 1928.744 | 0.000 |
| SR25 | Female | 9 | 0.000 | -0.021 | 0.021 | 0.000 | -0.018 | 0.017 | 13 | 1928.744 | 0.000 |
| SR25 | Female | 10 | -0.031 | -0.055 | -0.006 | 0.000 | -0.018 | 0.017 | 13 | 1928.744 | 0.000 |
| SR30 | Male | -5 | -0.003 | -0.020 | 0.014 | 0.001 | -0.015 | 0.016 | 13 | 4987.145 | 0.000 |
| SR30 | Male | -4 | 0.001 | -0.038 | 0.041 | 0.001 | -0.015 | 0.016 | 13 | 4987.145 | 0.000 |
| SR30 | Male | -3 | -0.004 | -0.019 | 0.012 | 0.001 | -0.015 | 0.016 | 13 | 4987.145 | 0.000 |
| SR30 | Male | -2 | 0.009 | -0.011 | 0.029 | 0.001 | -0.015 | 0.016 | 13 | 4987.145 | 0.000 |
| SR30 | Male | -1 | 0.000 | 0.000 | 0.000 |  |  |  | 13 | 4987.145 | 0.000 |
| SR30 | Male | 0 | -0.019 | -0.047 | 0.008 | -0.011 | -0.025 | 0.004 | 13 | 4987.145 | 0.000 |
| SR30 | Male | 1 | -0.017 | -0.041 | 0.007 | -0.011 | -0.025 | 0.004 | 13 | 4987.145 | 0.000 |
| SR30 | Male | 2 | -0.019 | -0.044 | 0.005 | -0.011 | -0.025 | 0.004 | 13 | 4987.145 | 0.000 |
| SR30 | Male | 3 | -0.010 | -0.042 | 0.022 | -0.011 | -0.025 | 0.004 | 13 | 4987.145 | 0.000 |
| SR30 | Male | 4 | -0.013 | -0.035 | 0.010 | -0.011 | -0.025 | 0.004 | 13 | 4987.145 | 0.000 |
| SR30 | Male | 5 | -0.013 | -0.043 | 0.018 | -0.011 | -0.025 | 0.004 | 13 | 4987.145 | 0.000 |
| SR30 | Male | 6 | 0.004 | -0.013 | 0.021 | -0.011 | -0.025 | 0.004 | 13 | 4987.145 | 0.000 |
| SR30 | Male | 7 | -0.006 | -0.034 | 0.021 | -0.011 | -0.025 | 0.004 | 13 | 4987.145 | 0.000 |
| SR30 | Male | 8 | -0.015 | -0.032 | 0.002 | -0.011 | -0.025 | 0.004 | 13 | 4987.145 | 0.000 |
| SR30 | Male | 9 | 0.006 | -0.018 | 0.030 | -0.011 | -0.025 | 0.004 | 13 | 4987.145 | 0.000 |
| SR30 | Male | 10 | -0.016 | -0.036 | 0.004 | -0.011 | -0.025 | 0.004 | 13 | 4987.145 | 0.000 |
| SR30 | Female | -5 | 0.030 | 0.002 | 0.058 | 0.018 | 0.002 | 0.033 | 13 | 1963.111 | 0.000 |
| SR30 | Female | -4 | 0.009 | -0.013 | 0.032 | 0.018 | 0.002 | 0.033 | 13 | 1963.111 | 0.000 |
| SR30 | Female | -3 | 0.012 | -0.008 | 0.031 | 0.018 | 0.002 | 0.033 | 13 | 1963.111 | 0.000 |
| SR30 | Female | -2 | 0.019 | 0.004 | 0.035 | 0.018 | 0.002 | 0.033 | 13 | 1963.111 | 0.000 |
| SR30 | Female | -1 | 0.000 | 0.000 | 0.000 |  |  |  | 13 | 1963.111 | 0.000 |
| SR30 | Female | 0 | 0.016 | 0.001 | 0.030 | -0.003 | -0.015 | 0.010 | 13 | 1963.111 | 0.000 |
| SR30 | Female | 1 | 0.002 | -0.011 | 0.016 | -0.003 | -0.015 | 0.010 | 13 | 1963.111 | 0.000 |
| SR30 | Female | 2 | -0.014 | -0.028 | 0.001 | -0.003 | -0.015 | 0.010 | 13 | 1963.111 | 0.000 |
| SR30 | Female | 3 | 0.003 | -0.027 | 0.033 | -0.003 | -0.015 | 0.010 | 13 | 1963.111 | 0.000 |
| SR30 | Female | 4 | -0.002 | -0.024 | 0.020 | -0.003 | -0.015 | 0.010 | 13 | 1963.111 | 0.000 |
| SR30 | Female | 5 | -0.015 | -0.042 | 0.013 | -0.003 | -0.015 | 0.010 | 13 | 1963.111 | 0.000 |
| SR30 | Female | 6 | -0.004 | -0.036 | 0.028 | -0.003 | -0.015 | 0.010 | 13 | 1963.111 | 0.000 |
| SR30 | Female | 7 | -0.002 | -0.024 | 0.020 | -0.003 | -0.015 | 0.010 | 13 | 1963.111 | 0.000 |
| SR30 | Female | 8 | -0.003 | -0.017 | 0.012 | -0.003 | -0.015 | 0.010 | 13 | 1963.111 | 0.000 |
| SR30 | Female | 9 | 0.003 | -0.013 | 0.019 | -0.003 | -0.015 | 0.010 | 13 | 1963.111 | 0.000 |
| SR30 | Female | 10 | -0.012 | -0.045 | 0.020 | -0.003 | -0.015 | 0.010 | 13 | 1963.111 | 0.000 |
| SR35 | Male | -5 | -0.004 | -0.020 | 0.011 | 0.006 | -0.004 | 0.016 | 13 | 4011.066 | 0.000 |
| SR35 | Male | -4 | 0.009 | -0.018 | 0.037 | 0.006 | -0.004 | 0.016 | 13 | 4011.066 | 0.000 |
| SR35 | Male | -3 | 0.004 | -0.014 | 0.022 | 0.006 | -0.004 | 0.016 | 13 | 4011.066 | 0.000 |
| SR35 | Male | -2 | 0.016 | -0.004 | 0.035 | 0.006 | -0.004 | 0.016 | 13 | 4011.066 | 0.000 |
| SR35 | Male | -1 | 0.000 | 0.000 | 0.000 |  |  |  | 13 | 4011.066 | 0.000 |
| SR35 | Male | 0 | -0.020 | -0.048 | 0.007 | -0.009 | -0.023 | 0.005 | 13 | 4011.066 | 0.000 |
| SR35 | Male | 1 | -0.018 | -0.039 | 0.003 | -0.009 | -0.023 | 0.005 | 13 | 4011.066 | 0.000 |
| SR35 | Male | 2 | -0.011 | -0.030 | 0.009 | -0.009 | -0.023 | 0.005 | 13 | 4011.066 | 0.000 |
| SR35 | Male | 3 | -0.010 | -0.030 | 0.011 | -0.009 | -0.023 | 0.005 | 13 | 4011.066 | 0.000 |
| SR35 | Male | 4 | 0.000 | -0.019 | 0.019 | -0.009 | -0.023 | 0.005 | 13 | 4011.066 | 0.000 |
| SR35 | Male | 5 | -0.007 | -0.035 | 0.020 | -0.009 | -0.023 | 0.005 | 13 | 4011.066 | 0.000 |
| SR35 | Male | 6 | 0.009 | -0.008 | 0.027 | -0.009 | -0.023 | 0.005 | 13 | 4011.066 | 0.000 |
| SR35 | Male | 7 | 0.002 | -0.031 | 0.034 | -0.009 | -0.023 | 0.005 | 13 | 4011.066 | 0.000 |
| SR35 | Male | 8 | -0.022 | -0.041 | -0.003 | -0.009 | -0.023 | 0.005 | 13 | 4011.066 | 0.000 |
| SR35 | Male | 9 | 0.001 | -0.024 | 0.025 | -0.009 | -0.023 | 0.005 | 13 | 4011.066 | 0.000 |
| SR35 | Male | 10 | -0.018 | -0.038 | 0.001 | -0.009 | -0.023 | 0.005 | 13 | 4011.066 | 0.000 |
| SR35 | Female | -5 | 0.023 | -0.005 | 0.051 | 0.010 | -0.011 | 0.032 | 13 | 446.944 | 0.000 |
| SR35 | Female | -4 | 0.000 | -0.033 | 0.033 | 0.010 | -0.011 | 0.032 | 13 | 446.944 | 0.000 |
| SR35 | Female | -3 | 0.009 | -0.021 | 0.038 | 0.010 | -0.011 | 0.032 | 13 | 446.944 | 0.000 |
| SR35 | Female | -2 | 0.009 | -0.007 | 0.026 | 0.010 | -0.011 | 0.032 | 13 | 446.944 | 0.000 |
| SR35 | Female | -1 | 0.000 | 0.000 | 0.000 |  |  |  | 13 | 446.944 | 0.000 |
| SR35 | Female | 0 | 0.002 | -0.013 | 0.017 | -0.005 | -0.018 | 0.007 | 13 | 446.944 | 0.000 |
| SR35 | Female | 1 | 0.006 | -0.019 | 0.030 | -0.005 | -0.018 | 0.007 | 13 | 446.944 | 0.000 |
| SR35 | Female | 2 | -0.003 | -0.018 | 0.011 | -0.005 | -0.018 | 0.007 | 13 | 446.944 | 0.000 |
| SR35 | Female | 3 | -0.010 | -0.047 | 0.027 | -0.005 | -0.018 | 0.007 | 13 | 446.944 | 0.000 |
| SR35 | Female | 4 | -0.022 | -0.048 | 0.003 | -0.005 | -0.018 | 0.007 | 13 | 446.944 | 0.000 |
| SR35 | Female | 5 | -0.004 | -0.034 | 0.026 | -0.005 | -0.018 | 0.007 | 13 | 446.944 | 0.000 |
| SR35 | Female | 6 | 0.002 | -0.028 | 0.031 | -0.005 | -0.018 | 0.007 | 13 | 446.944 | 0.000 |
| SR35 | Female | 7 | -0.005 | -0.024 | 0.015 | -0.005 | -0.018 | 0.007 | 13 | 446.944 | 0.000 |
| SR35 | Female | 8 | -0.013 | -0.028 | 0.003 | -0.005 | -0.018 | 0.007 | 13 | 446.944 | 0.000 |
| SR35 | Female | 9 | 0.000 | -0.019 | 0.019 | -0.005 | -0.018 | 0.007 | 13 | 446.944 | 0.000 |
| SR35 | Female | 10 | -0.013 | -0.037 | 0.011 | -0.005 | -0.018 | 0.007 | 13 | 446.944 | 0.000 |
| SR40 | Male | -5 | -0.006 | -0.027 | 0.015 | 0.009 | -0.002 | 0.021 | 13 | 1200.247 | 0.000 |
| SR40 | Male | -4 | 0.012 | -0.010 | 0.034 | 0.009 | -0.002 | 0.021 | 13 | 1200.247 | 0.000 |
| SR40 | Male | -3 | 0.012 | -0.012 | 0.036 | 0.009 | -0.002 | 0.021 | 13 | 1200.247 | 0.000 |
| SR40 | Male | -2 | 0.019 | -0.002 | 0.041 | 0.009 | -0.002 | 0.021 | 13 | 1200.247 | 0.000 |
| SR40 | Male | -1 | 0.000 | 0.000 | 0.000 |  |  |  | 13 | 1200.247 | 0.000 |
| SR40 | Male | 0 | -0.007 | -0.032 | 0.017 | -0.003 | -0.021 | 0.014 | 13 | 1200.247 | 0.000 |
| SR40 | Male | 1 | -0.001 | -0.028 | 0.026 | -0.003 | -0.021 | 0.014 | 13 | 1200.247 | 0.000 |
| SR40 | Male | 2 | -0.012 | -0.039 | 0.015 | -0.003 | -0.021 | 0.014 | 13 | 1200.247 | 0.000 |
| SR40 | Male | 3 | 0.000 | -0.028 | 0.029 | -0.003 | -0.021 | 0.014 | 13 | 1200.247 | 0.000 |
| SR40 | Male | 4 | 0.000 | -0.024 | 0.023 | -0.003 | -0.021 | 0.014 | 13 | 1200.247 | 0.000 |
| SR40 | Male | 5 | -0.001 | -0.029 | 0.027 | -0.003 | -0.021 | 0.014 | 13 | 1200.247 | 0.000 |
| SR40 | Male | 6 | 0.008 | -0.007 | 0.023 | -0.003 | -0.021 | 0.014 | 13 | 1200.247 | 0.000 |
| SR40 | Male | 7 | 0.007 | -0.023 | 0.037 | -0.003 | -0.021 | 0.014 | 13 | 1200.247 | 0.000 |
| SR40 | Male | 8 | -0.013 | -0.030 | 0.004 | -0.003 | -0.021 | 0.014 | 13 | 1200.247 | 0.000 |
| SR40 | Male | 9 | -0.005 | -0.031 | 0.021 | -0.003 | -0.021 | 0.014 | 13 | 1200.247 | 0.000 |
| SR40 | Male | 10 | -0.014 | -0.036 | 0.009 | -0.003 | -0.021 | 0.014 | 13 | 1200.247 | 0.000 |
| SR40 | Female | -5 | 0.026 | -0.008 | 0.060 | 0.010 | -0.017 | 0.037 | 13 | 1674.865 | 0.000 |
| SR40 | Female | -4 | -0.003 | -0.035 | 0.029 | 0.010 | -0.017 | 0.037 | 13 | 1674.865 | 0.000 |
| SR40 | Female | -3 | 0.011 | -0.026 | 0.048 | 0.010 | -0.017 | 0.037 | 13 | 1674.865 | 0.000 |
| SR40 | Female | -2 | 0.005 | -0.017 | 0.028 | 0.010 | -0.017 | 0.037 | 13 | 1674.865 | 0.000 |
| SR40 | Female | -1 | 0.000 | 0.000 | 0.000 |  |  |  | 13 | 1674.865 | 0.000 |
| SR40 | Female | 0 | 0.002 | -0.014 | 0.017 | -0.008 | -0.026 | 0.010 | 13 | 1674.865 | 0.000 |
| SR40 | Female | 1 | 0.002 | -0.029 | 0.032 | -0.008 | -0.026 | 0.010 | 13 | 1674.865 | 0.000 |
| SR40 | Female | 2 | -0.009 | -0.022 | 0.004 | -0.008 | -0.026 | 0.010 | 13 | 1674.865 | 0.000 |
| SR40 | Female | 3 | -0.016 | -0.059 | 0.027 | -0.008 | -0.026 | 0.010 | 13 | 1674.865 | 0.000 |
| SR40 | Female | 4 | -0.030 | -0.059 | -0.001 | -0.008 | -0.026 | 0.010 | 13 | 1674.865 | 0.000 |
| SR40 | Female | 5 | -0.006 | -0.042 | 0.030 | -0.008 | -0.026 | 0.010 | 13 | 1674.865 | 0.000 |
| SR40 | Female | 6 | 0.002 | -0.030 | 0.033 | -0.008 | -0.026 | 0.010 | 13 | 1674.865 | 0.000 |
| SR40 | Female | 7 | -0.001 | -0.024 | 0.023 | -0.008 | -0.026 | 0.010 | 13 | 1674.865 | 0.000 |
| SR40 | Female | 8 | -0.017 | -0.045 | 0.011 | -0.008 | -0.026 | 0.010 | 13 | 1674.865 | 0.000 |
| SR40 | Female | 9 | -0.003 | -0.029 | 0.022 | -0.008 | -0.026 | 0.010 | 13 | 1674.865 | 0.000 |
| SR40 | Female | 10 | -0.012 | -0.034 | 0.011 | -0.008 | -0.026 | 0.010 | 13 | 1674.865 | 0.000 |
|  |  |  |  |  |  |  |  |  |  |  |  |

*Note: TNC = Total Number of Children; CB = Child Before; MB = Married Before; SR = Same Region. DF, Chi2 and P are statistics from pretrend tests.*

***S5: Event study estimates of the effects of local college establishments at age* 18, 22, and 24 *on higher educational attainment, by sex***

**Age 18**


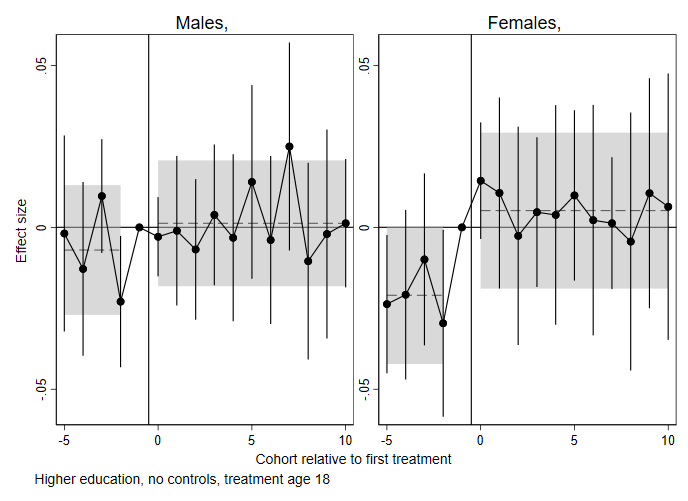


*Note: Points are coefficients for time dummies from difference-in-differences/event study models estimated with csdid, with 95 % confidence intervals. Dashed lines represent the pre- and post-treatment average effects, with 95 % confidence intervals shown as shaded areas.*

**Age 22**


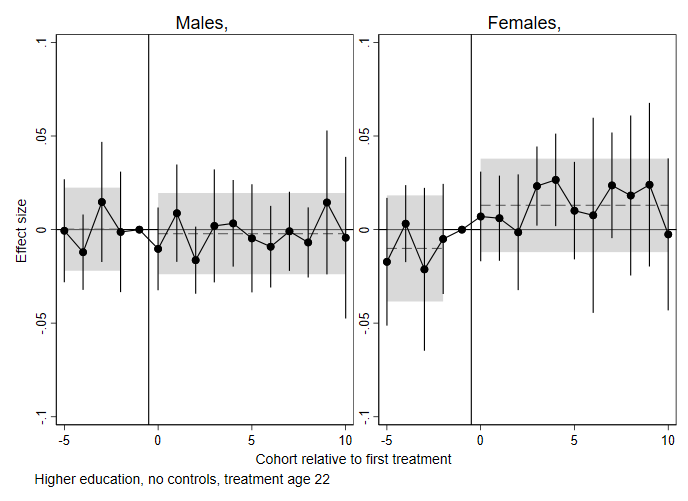


*Note: Points are coefficients for time dummies from difference-in-differences/event study models estimated with csdid, with 95 % confidence intervals. Dashed lines represent the pre- and post-treatment average effects, with 95 % confidence intervals shown as shaded areas.*

**Age 24**


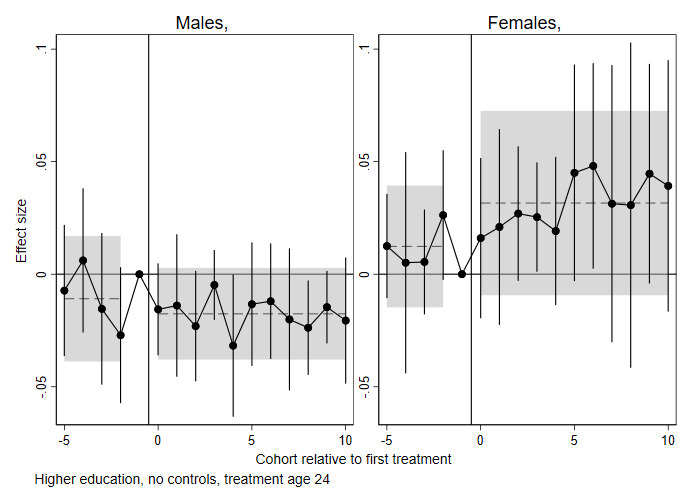


*Note: Points are coefficients for time dummies from difference-in-differences/event study models estimated with csdid, with 95 % confidence intervals. Dashed lines represent the pre- and post-treatment average effects, with 95 % confidence intervals shown as shaded areas.*

***S6: Event study estimates of the effects of local college establishments at age 18, 22, and 24 on the total number of children born before age 40, by sex***

**Age 18**


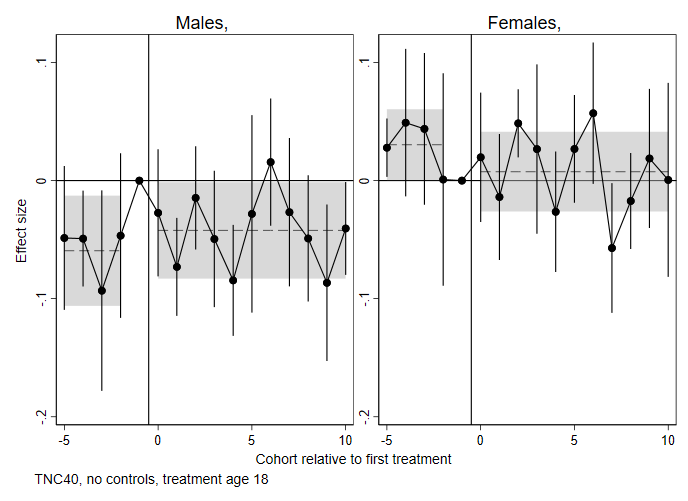


*Note: Points are coefficients for time dummies from difference-in-differences/event study models estimated with csdid, with 95 % confidence intervals. Dashed lines represent the pre- and post-treatment average effects, with 95 % confidence intervals shown as shaded areas.*

**Age 22**


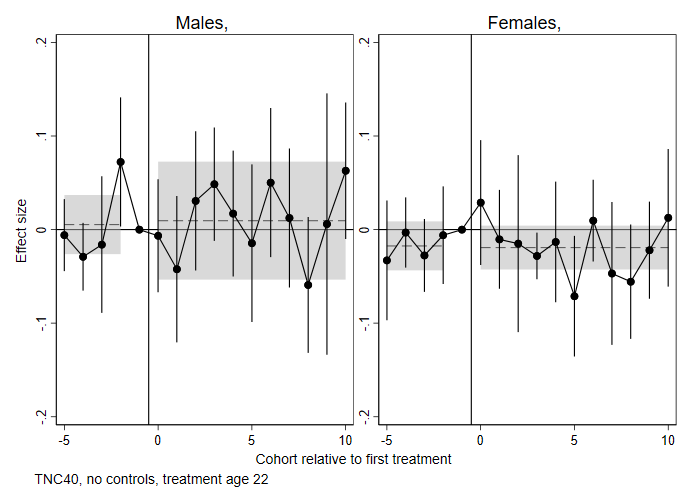


*Note: Points are coefficients for time dummies from difference-in-differences/event study models estimated with csdid, with 95 % confidence intervals. Dashed lines represent the pre- and post-treatment average effects, with 95 % confidence intervals shown as shaded areas.*

**Age 24**


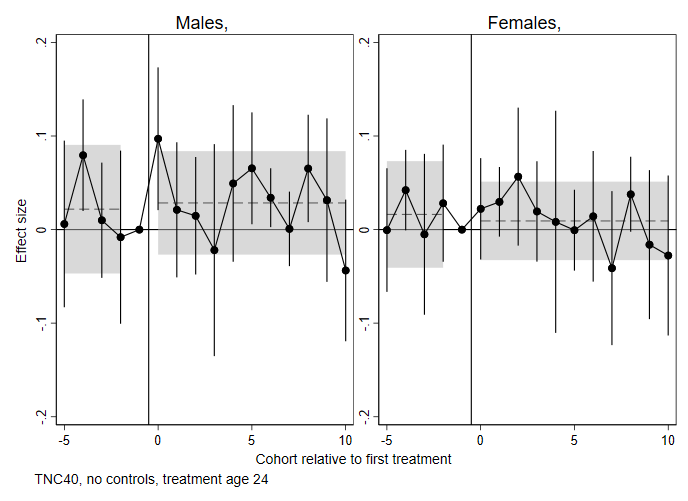


*Note: Points are coefficients for time dummies from difference-in-differences/event study models estimated with csdid, with 95 % confidence intervals. Dashed lines represent the pre- and post-treatment average effects, with 95 % confidence intervals shown as shaded areas.*

***S7: Event study estimates of the effects of local college establishments at age 18, 22, and 24 on having children before age 20-40, by sex***

**Age 18**


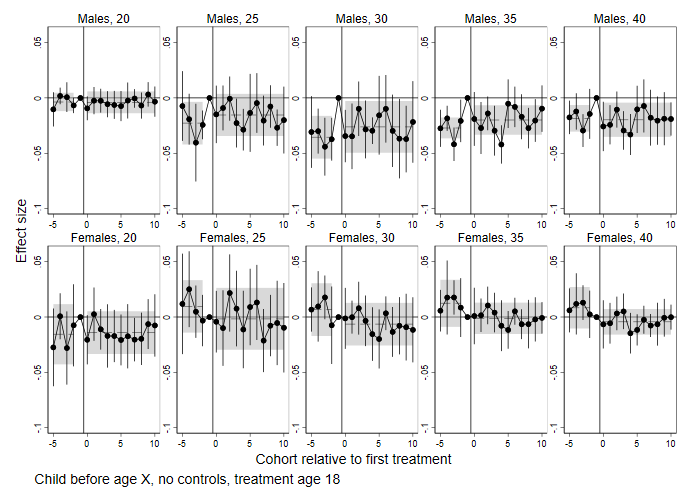


*Note: Points are coefficients for time dummies from difference-in-differences/event study models estimated with csdid, with 95 % confidence intervals. Dashed lines represent the pre- and post-treatment average effects, with 95 % confidence intervals shown as shaded areas.*

**Age 22**


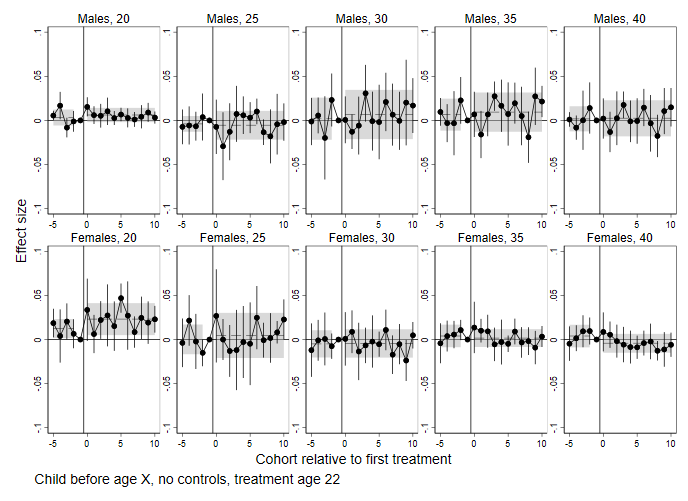


*Note: Points are coefficients for time dummies from difference-in-differences/event study models estimated with csdid, with 95 % confidence intervals. Dashed lines represent the pre- and post-treatment average effects, with 95 % confidence intervals shown as shaded areas.*

**Age 24**


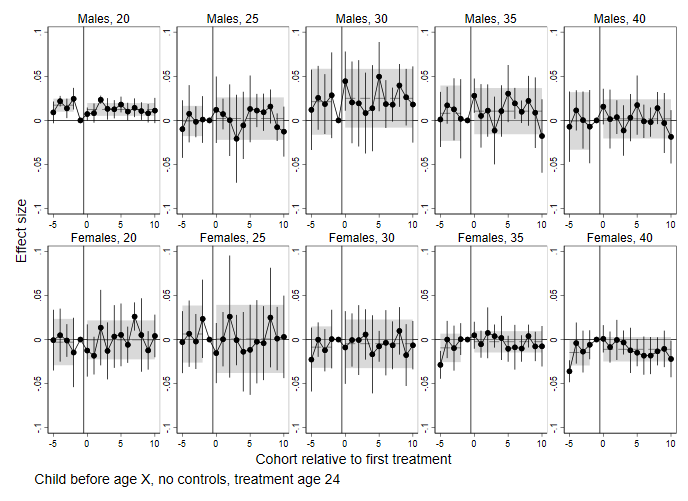


*Note: Points are coefficients for time dummies from difference-in-differences/event study models estimated with csdid, with 95 % confidence intervals. Dashed lines represent the pre- and post-treatment average effects, with 95 % confidence intervals shown as shaded areas.*

***S8: Event study estimates of the effects of local college establishments at age 18, 22, and 24 on being married before age 20-40, by sex***

**Age 18**


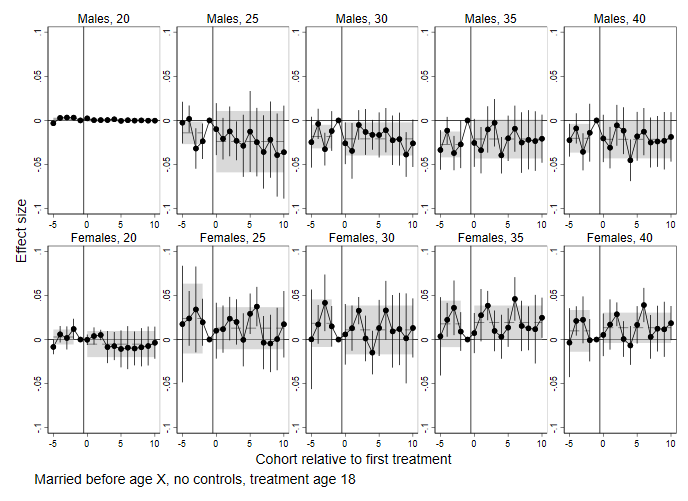


*Note: Points are coefficients for time dummies from difference-in-differences/event study models estimated with csdid, with 95 % confidence intervals. Dashed lines represent the pre- and post-treatment average effects, with 95 % confidence intervals shown as shaded areas.*

**Age 22**


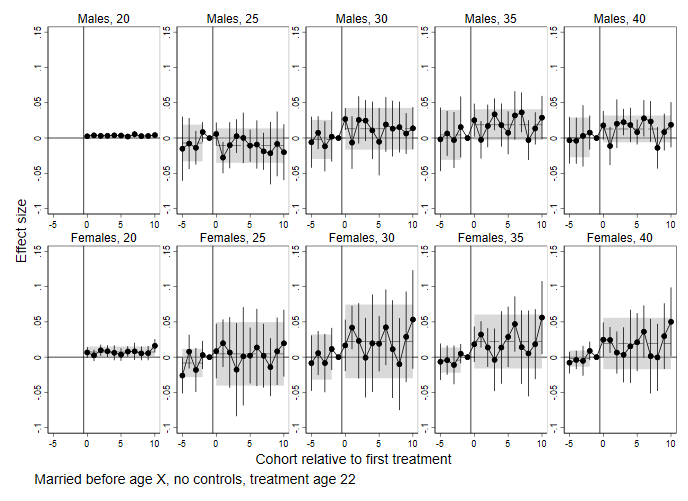


*Note: Points are coefficients for time dummies from difference-in-differences/event study models estimated with csdid, with 95 % confidence intervals. Dashed lines represent the pre- and post-treatment average effects, with 95 % confidence intervals shown as shaded areas. Estimates for the impact of college establishments at age 22 on being married before age 20 are not particularly meaningful.*

**Age 24**


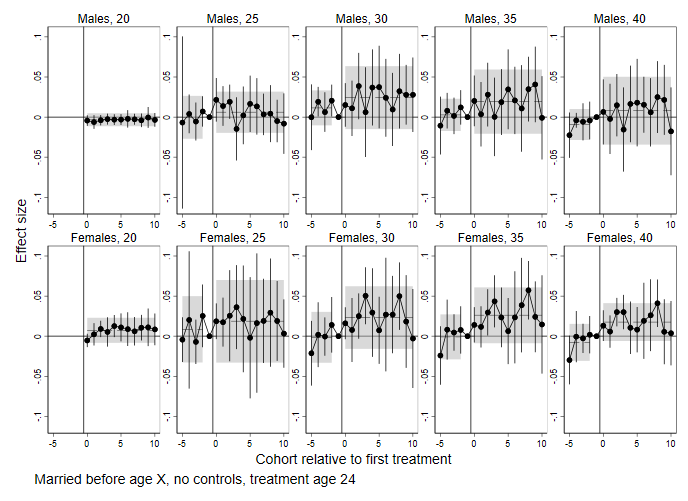


*Note: Points are coefficients for time dummies from difference-in-differences/event study models estimated with csdid, with 95 % confidence intervals. Dashed lines represent the pre- and post-treatment average effects, with 95 % confidence intervals shown as shaded areas. Estimates for the impact of college establishments at age 24 on being married before age 20 are not particularly meaningful.*

***S9: Event study estimates of the effects of local college establishments at age 20 on higher educational attainment, by sex, with control variables***


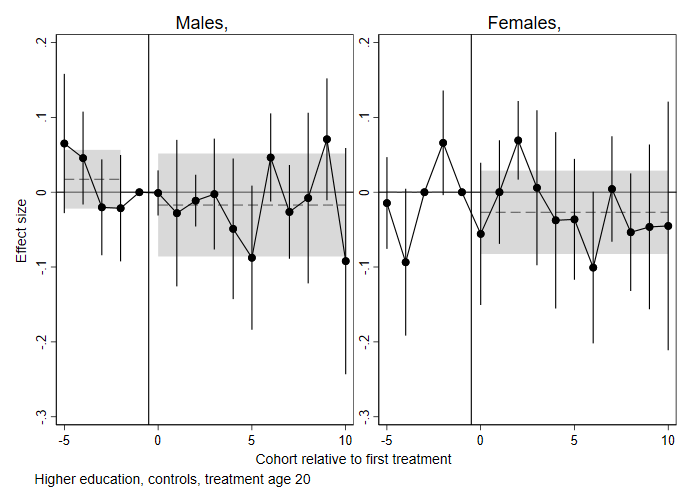


*Note: Points are coefficients for time dummies from difference-in-differences/event study models estimated with csdid, with 95 % confidence intervals. Dashed lines represent the pre- and post-treatment average effects, with 95 % confidence intervals shown as shaded areas. Pre-treatment coefficients that are exactly zero and shown without confidence intervals were omitted from the model estimation due to collinearity. This also prevents the estimation of pre-treatment averages.*

***S10: Event study estimates of the effects of local college establishments at age 20 on the total number of children born before age 40, by sex, with control variables***


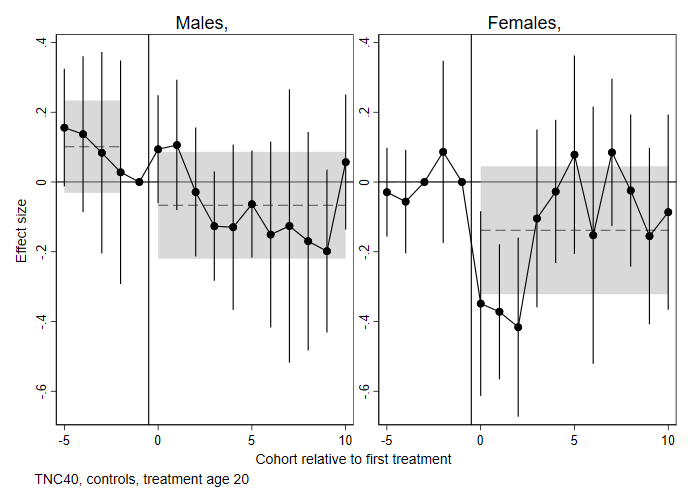


*Note: Points are coefficients for time dummies from difference-in-differences/event study models estimated with csdid, with 95 % confidence intervals. Dashed lines represent the pre- and post-treatment average effects, with 95 % confidence intervals shown as shaded areas. Pre-treatment coefficients that are exactly zero and shown without confidence intervals were omitted from the model estimation due to collinearity. This also prevents the estimation of pre-treatment averages.*

***S11: Event study estimates of the effects of local college establishments at age 20 on having children before age 20-40, by sex, with control variables***


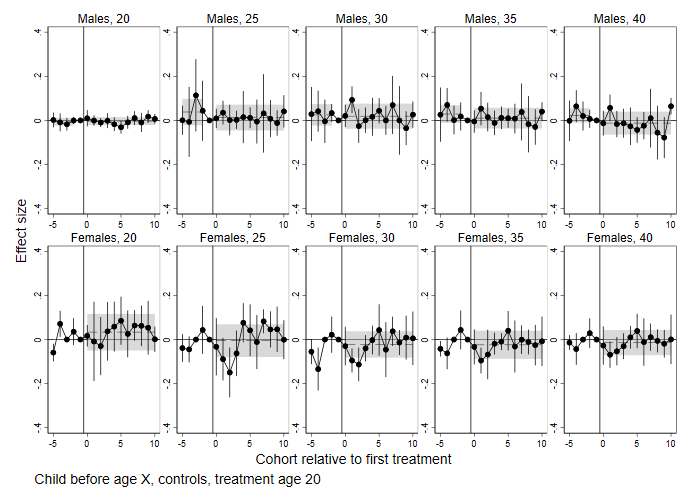


*Note: Points are coefficients for time dummies from difference-in-differences/event study models estimated with csdid, with 95 % confidence intervals. Dashed lines represent the pre- and post-treatment average effects, with 95 % confidence intervals shown as shaded areas. Pre-treatment coefficients that are exactly zero and shown without confidence intervals were omitted from the model estimation due to collinearity. This also prevents the estimation of pre-treatment averages.*

***S12: Event study estimates of the effects of local college establishments at age 20 on being married before age 20-40, by sex, with control variables***


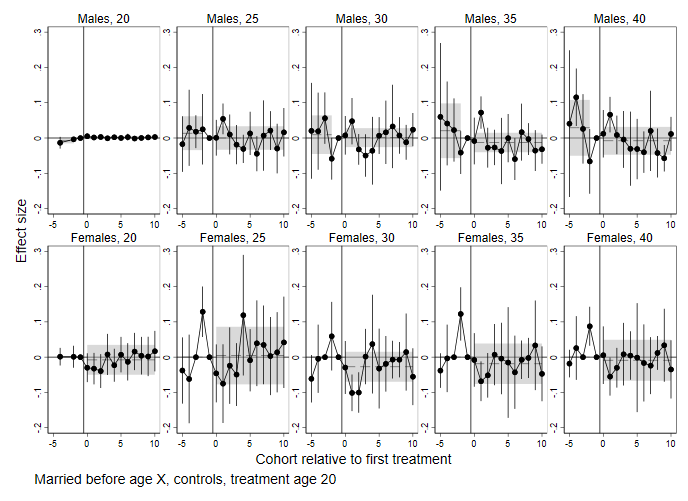


*Note: Points are coefficients for time dummies from difference-in-differences/event study models estimated with csdid, with 95 % confidence intervals. Dashed lines represent the pre- and post-treatment average effects, with 95 % confidence intervals shown as shaded areas. Pre-treatment coefficients that are exactly zero and shown without confidence intervals were omitted from the model estimation due to collinearity. This also prevents the estimation of pre-treatment averages. Some pre-treatment coefficients for marriage before age 20 could not be estimated and were manually omitted.*

***S13: Event study estimates of the effects of local college establishments at age 20 on higher educational attainment, by sex, with municipalities as the geographic unit***


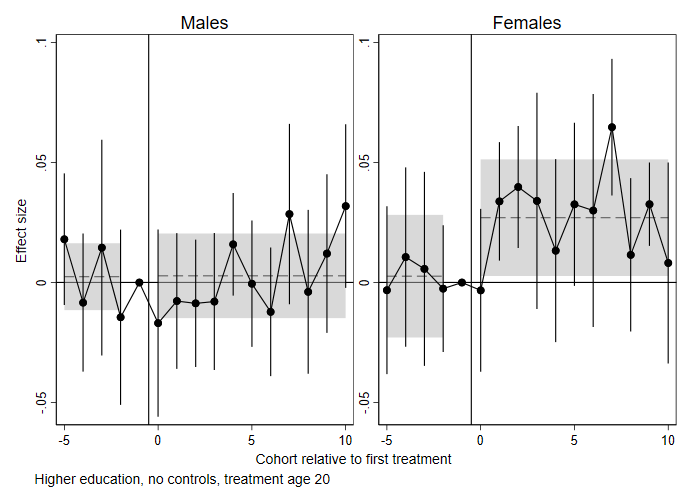


*Note: Points are coefficients for time dummies from difference-in-differences/event study models estimated with csdid, with 95 % confidence intervals. Dashed lines represent the pre- and post-treatment average effects, with 95 % confidence intervals shown as shaded areas.*

***S14: Event study estimates of the effects of local college establishments at age 20 on the total number of children born before age 40, by sex, with municipalities as the geographic unit***


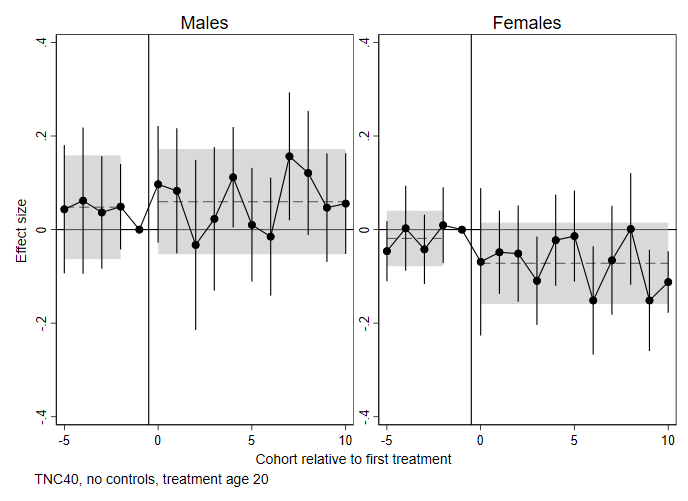


*Note: Points are coefficients for time dummies from difference-in-differences/event study models estimated with csdid, with 95 % confidence intervals. Dashed lines represent the pre- and post-treatment average effects, with 95 % confidence intervals shown as shaded areas.*

***S15: Event study estimates of the effects of local college establishments at age 20 on having children before age 20-40, by sex, with municipalities as the geographic unit***


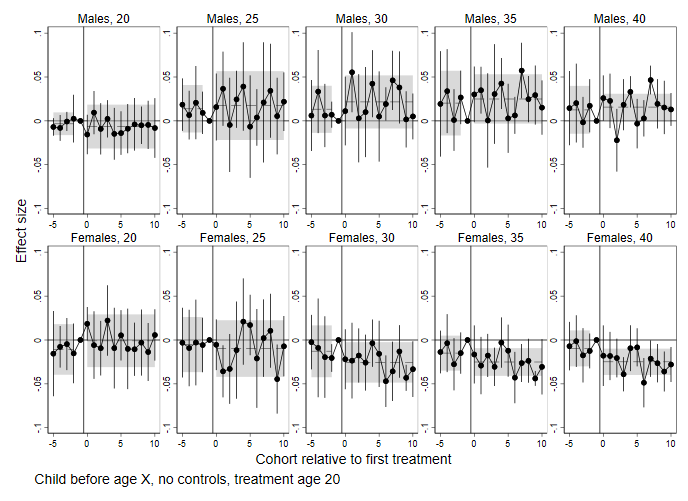


*Note: Points are coefficients for time dummies from difference-in-differences/event study models estimated with csdid, with 95 % confidence intervals. Dashed lines represent the pre- and post-treatment average effects, with 95 % confidence intervals shown as shaded areas.*

***S16: Event study estimates of the effects of local college establishments at age 20 on being married before age 20-40, by sex, with municipalities as the geographic unit***


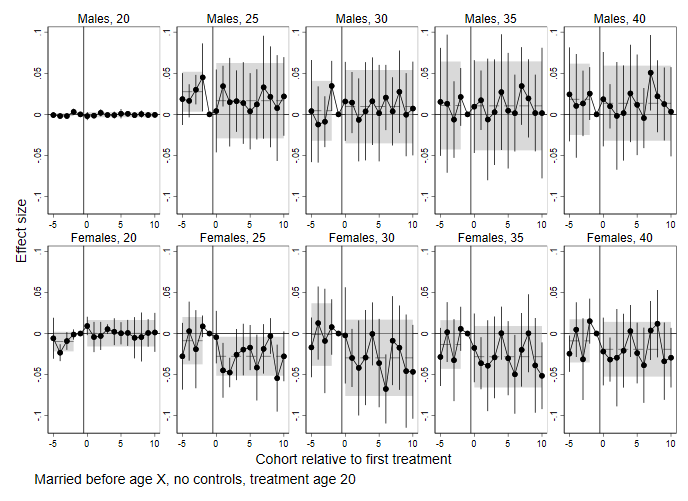


*Note: Points are coefficients for time dummies from difference-in-differences/event study models estimated with csdid, with 95 % confidence intervals. Dashed lines represent the pre- and post-treatment average effects, with 95 % confidence intervals shown as shaded areas.*

***S17: Event study estimates of the effects of local college establishments at age 20 on residing in the same region at ages 25-40, by sex, with municipalities as the geographic unit***


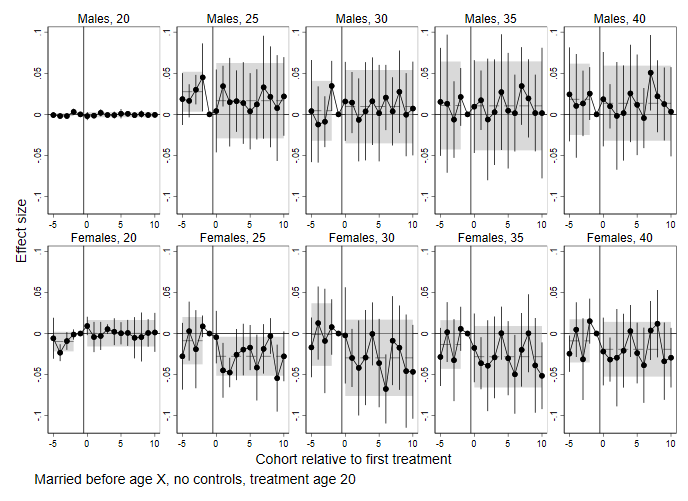


*Note: Points are coefficients for time dummies from difference-in-differences/event study models estimated with csdid, with 95 % confidence intervals. Dashed lines represent the pre- and post-treatment average effects, with 95 % confidence intervals shown as shaded areas.*

***S18: Pre-treatment trend test statistics***

| **Outcome** | **Sex** | **P-value** | **DF** | **Chi2** |
| --- | --- | --- | --- | --- |
| Higher ed. | Female | 0.0000 | 13 | 1588.936 |
| Higher ed. | Male | 0.0000 | 13 | 1042.712 |
| TNC40 | Male | 0.0000 | 13 | 13446.39 |
| TNC40 | Female | 0.0000 | 13 | 1150.662 |
| CB20 | Male | 0.0000 | 13 | 545.2011 |
| CB20 | Female | 0.0000 | 13 | 13684.72 |
| CB25 | Male | 0.0000 | 13 | 12272.93 |
| CB25 | Female | 0.0000 | 13 | 5301.372 |
| CB30 | Male | 0.0000 | 13 | 11545.47 |
| CB30 | Female | 0.0000 | 13 | 613.6784 |
| CB35 | Male | 0.0000 | 13 | 14699.6 |
| CB35 | Female | 0.0000 | 13 | 549.9495 |
| CB40 | Male | 0.0000 | 13 | 2475.891 |
| CB40 | Female | 0.0000 | 13 | 6725.86 |
| MB20 | Male | 0.0000 | 5 | 28.61816 |
| MB20 | Female | 0.0136 | 5 | 14.33543 |
| MB25 | Male | 0.0000 | 12 | 2614.994 |
| MB25 | Female | 0.0000 | 12 | 1951.347 |
| MB30 | Male | 0.0000 | 13 | 13442.01 |
| MB30 | Female | 0.0000 | 13 | 12630.46 |
| MB35 | Male | 0.0000 | 13 | 3115.132 |
| MB35 | Female | 0.0000 | 13 | 1445.061 |
| MB40 | Male | 0.0000 | 13 | 2596.221 |
| MB40 | Female | 0.0000 | 13 | 1335.586 |
| SR25 | Male | 0.0000 | 13 | 3158.898 |
| SR25 | Female | 0.0000 | 13 | 1928.744 |
| SR25 | Male | 0.0000 | 13 | 4987.145 |
| SR25 | Female | 0.0000 | 13 | 1963.111 |
| SR25 | Male | 0.0000 | 13 | 4011.066 |
| SR25 | Female | 0.0000 | 13 | 446.9436 |
| SR25 | Male | 0.0000 | 13 | 1200.247 |
| SR25 | Female | 0.0000 | 13 | 1674.865 |

*Note: TNC = Total Number of Children; CB = Child Before; MB = Married Before; SR = Same Region. Estimated using csdid’s estat pretrend command which estimates the chi2 statistic of the null hypothesis that all pretreatment ATTGT's (Average treatment effects of the treated for group G at time T) are statistically equal to zero.*

***S19:*** ***Event study estimates of the effects of local college establishments at age 20 on higher educational attainment, by sex***


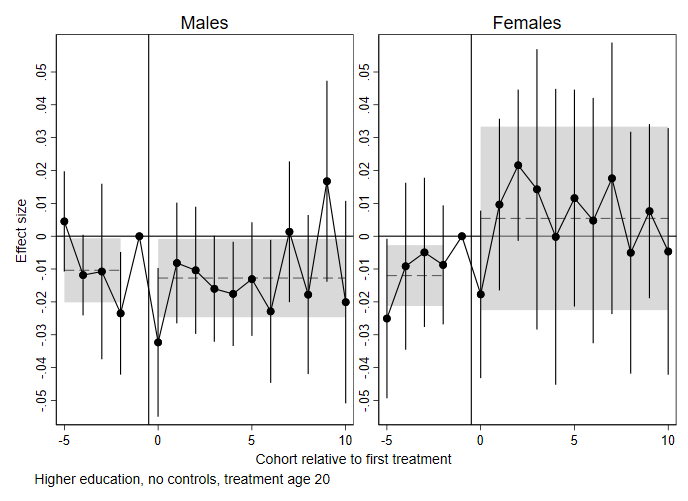


*Note: Points are coefficients for time dummies from difference-in-differences/event study models estimated with csdid, with 95 % confidence intervals. Dashed lines represent the pre- and post-treatment average effects, with 95 % confidence intervals shown as shaded areas.*

**Results from S19 in tabular form:**

| Outcome | Sex | Time | Coef. | Lower CI | Upper CI | Avg. coef. | Avg. lower CI | Avg. upper CI | DF | Chi2 | P |
| --- | --- | --- | --- | --- | --- | --- | --- | --- | --- | --- | --- |
| HE | Male | -5 | 0.005 | -0.011 | 0.020 | -0.010 | -0.020 | -0.001 | 13 | 1042.712 | 0.000 |
| HE | Male | -4 | -0.012 | -0.024 | 0.000 | -0.010 | -0.020 | -0.001 | 13 | 1042.712 | 0.000 |
| HE | Male | -3 | -0.011 | -0.037 | 0.016 | -0.010 | -0.020 | -0.001 | 13 | 1042.712 | 0.000 |
| HE | Male | -2 | -0.023 | -0.042 | -0.005 | -0.010 | -0.020 | -0.001 | 13 | 1042.712 | 0.000 |
| HE | Male | -1 | 0.000 | 0.000 | 0.000 |  |  |  | 13 | 1042.712 | 0.000 |
| HE | Male | 0 | -0.032 | -0.055 | -0.010 | -0.013 | -0.025 | -0.001 | 13 | 1042.712 | 0.000 |
| HE | Male | 1 | -0.008 | -0.027 | 0.010 | -0.013 | -0.025 | -0.001 | 13 | 1042.712 | 0.000 |
| HE | Male | 2 | -0.010 | -0.030 | 0.009 | -0.013 | -0.025 | -0.001 | 13 | 1042.712 | 0.000 |
| HE | Male | 3 | -0.016 | -0.032 | 0.000 | -0.013 | -0.025 | -0.001 | 13 | 1042.712 | 0.000 |
| HE | Male | 4 | -0.018 | -0.033 | -0.002 | -0.013 | -0.025 | -0.001 | 13 | 1042.712 | 0.000 |
| HE | Male | 5 | -0.013 | -0.030 | 0.004 | -0.013 | -0.025 | -0.001 | 13 | 1042.712 | 0.000 |
| HE | Male | 6 | -0.023 | -0.045 | -0.001 | -0.013 | -0.025 | -0.001 | 13 | 1042.712 | 0.000 |
| HE | Male | 7 | 0.001 | -0.020 | 0.023 | -0.013 | -0.025 | -0.001 | 13 | 1042.712 | 0.000 |
| HE | Male | 8 | -0.018 | -0.042 | 0.006 | -0.013 | -0.025 | -0.001 | 13 | 1042.712 | 0.000 |
| HE | Male | 9 | 0.017 | -0.014 | 0.047 | -0.013 | -0.025 | -0.001 | 13 | 1042.712 | 0.000 |
| HE | Male | 10 | -0.020 | -0.051 | 0.011 | -0.013 | -0.025 | -0.001 | 13 | 1042.712 | 0.000 |
| HE | Female | -5 | -0.025 | -0.049 | -0.001 | -0.012 | -0.021 | -0.003 | 13 | 1588.936 | 0.000 |
| HE | Female | -4 | -0.009 | -0.035 | 0.016 | -0.012 | -0.021 | -0.003 | 13 | 1588.936 | 0.000 |
| HE | Female | -3 | -0.005 | -0.028 | 0.018 | -0.012 | -0.021 | -0.003 | 13 | 1588.936 | 0.000 |
| HE | Female | -2 | -0.009 | -0.027 | 0.009 | -0.012 | -0.021 | -0.003 | 13 | 1588.936 | 0.000 |
| HE | Female | -1 | 0.000 | 0.000 | 0.000 |  |  |  | 13 | 1588.936 | 0.000 |
| HE | Female | 0 | -0.018 | -0.043 | 0.008 | 0.005 | -0.023 | 0.033 | 13 | 1588.936 | 0.000 |
| HE | Female | 1 | 0.010 | -0.016 | 0.036 | 0.005 | -0.023 | 0.033 | 13 | 1588.936 | 0.000 |
| HE | Female | 2 | 0.022 | -0.001 | 0.045 | 0.005 | -0.023 | 0.033 | 13 | 1588.936 | 0.000 |
| HE | Female | 3 | 0.014 | -0.028 | 0.057 | 0.005 | -0.023 | 0.033 | 13 | 1588.936 | 0.000 |
| HE | Female | 4 | 0.000 | -0.045 | 0.045 | 0.005 | -0.023 | 0.033 | 13 | 1588.936 | 0.000 |
| HE | Female | 5 | 0.012 | -0.021 | 0.045 | 0.005 | -0.023 | 0.033 | 13 | 1588.936 | 0.000 |
| HE | Female | 6 | 0.005 | -0.033 | 0.042 | 0.005 | -0.023 | 0.033 | 13 | 1588.936 | 0.000 |
| HE | Female | 7 | 0.018 | -0.024 | 0.059 | 0.005 | -0.023 | 0.033 | 13 | 1588.936 | 0.000 |
| HE | Female | 8 | -0.005 | -0.042 | 0.032 | 0.005 | -0.023 | 0.033 | 13 | 1588.936 | 0.000 |
| HE | Female | 9 | 0.008 | -0.019 | 0.034 | 0.005 | -0.023 | 0.033 | 13 | 1588.936 | 0.000 |
| HE | Female | 10 | -0.005 | -0.042 | 0.033 | 0.005 | -0.023 | 0.033 | 13 | 1588.936 | 0.000 |

***S18: Event study estimates of the effects of local college establishments at age 20 on population size in the region***


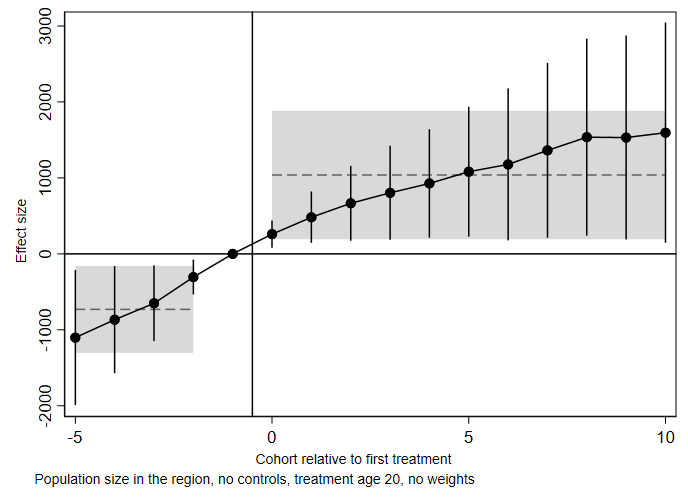


*Note: Points are coefficients for time dummies from difference-in-differences/event study models estimated with csdid, with 95 % confidence intervals. Dashed lines represent the pre- and post-treatment average effects, with 95 % confidence intervals shown as shaded areas. All regions weighted equally.*
